# Genus *Cucumis*: Traditional uses, phytochemistry, pharmacology, clinical application, and toxicology

**DOI:** 10.1016/j.chmed.2026.02.005

**Published:** 2026-02-12

**Authors:** Hesham M. El-Sayed, Engy A. Mahrous, Dalia M. Rasheed, Essam Abdel-Sattar

**Affiliations:** a Pharmacognosy Department, Faculty of Pharmacy, October 6 University, Giza 12585, Egypt; b Pharmacognosy Department, Faculty of Pharmacy, Cairo University, Cairo 11562, Egypt

**Keywords:** acylated flavonoids, *Cucumis*, Cucurbitaceae, cucurbitacins, ethnomedicine, pharmacology, phytochemistry, toxicity

## Abstract

The genus *Cucumis* L. (Cucurbitaceae), flourishes across temperate, tropical, and subtropical regions. Species within the genus have a long-standing history in traditional ethnomedicinal practices throughout Asia and Africa, addressing a wide spectrum of ailments, including gastrointestinal, metabolic, urogenital, hepatic, dermatological, cardiovascular, respiratory, inflammatory, and infectious disorders. Despite this extensive traditional use, the full therapeutic potential of *Cucumis* remains underexplored.

This review comprehensively explores the traditional ethnomedicinal applications, phytochemical composition, pharmacological activities, clinical evidence, and toxicological profiles of *Cucumis* species. A systematic survey of internationally recognized scientific databases and authoritative repositories, including Web of Science, Scopus, PubMed, ScienceDirect, and Google Scholar, and revealed over 428 bioactive compounds spanning through diverse phytochemical classes. These classes include steroids, triterpenoids (predominantly cucurbitacins), flavonoids (particularly *C*,*O*-glycosylflavones), coumarins, other phenolics, and additional secondary metabolites. Collectively, these compounds demonstrate multifaceted bioactivities, encompassing anti-inflammatory, antihypertensive, anti-diabetic, anti-cancer, organ-protective, antimicrobial, and antiviral properties, as validated through *in vitro*, *in vivo*, and limited clinical studies. Nonetheless, the molecular mechanisms underpinning these pharmacological effects remain inadequately elucidated.

By integrating traditional ethnomedicinal knowledge with contemporary research approaches, this review highlights the necessity of advancing scientific insight into the genus *Cucumis* and its bioactive constituents. Future research should focus on comprehensive phytochemical profiling, mechanistic elucidation, clinical evaluations, and rigorous toxicological assessments to ensure the safe and effective application of various *Cucumis* species in modern herbal medicine.

## 1 Introduction

*Cucumis* L., a genus of the Cucurbitaceae family, comprises 304 species of annual or perennial herbaceous plants, of which 60 (20%) are taxonomically accepted, 210 (69%) are considered synonyms, and 34 (11%) remain unplaced. The genus includes about 30 African and 25 Asian and Australian species, distributed across temperate, tropical, and subtropical regions such as South Africa, Ethiopia, and regions of the Himalayas covering China, India, and Pakistan. These data were compiled from the World Flora Online (WFO; https://www.worldfloraonline.org/), WFO Plant List (WFOPL; https://wfoplantlist.org/), and the Global Biodiversity Information Facility (GBIF; https://www.gbif.org/).

Botanically, *Cucumis* species are long, trailing herbs that can be annual or perennial. They have angular or lobed leaves with simple tendrils for climbing or supporting. The stems of these plants are branched and covered in coarse and stiff hairs. They are mostly monoecious, but dioecious and andromonoecious forms also occur. Yellow flowers are fascicled or solitary, usually trimerous, rarely pentamerous, and often borne at every node. The stalk or pedicel that supports the female flower develops into one that supports the fruit. Fruits are usually fleshy, containing many tanned or white seeds of uniform shape and smooth surface (Chen and Zhou, 2011, Lija and Beevy, 2021). More details concerning the botanical description of each organ of the species are provided in WFO and WFOPL databases.

**Fig. 1:**
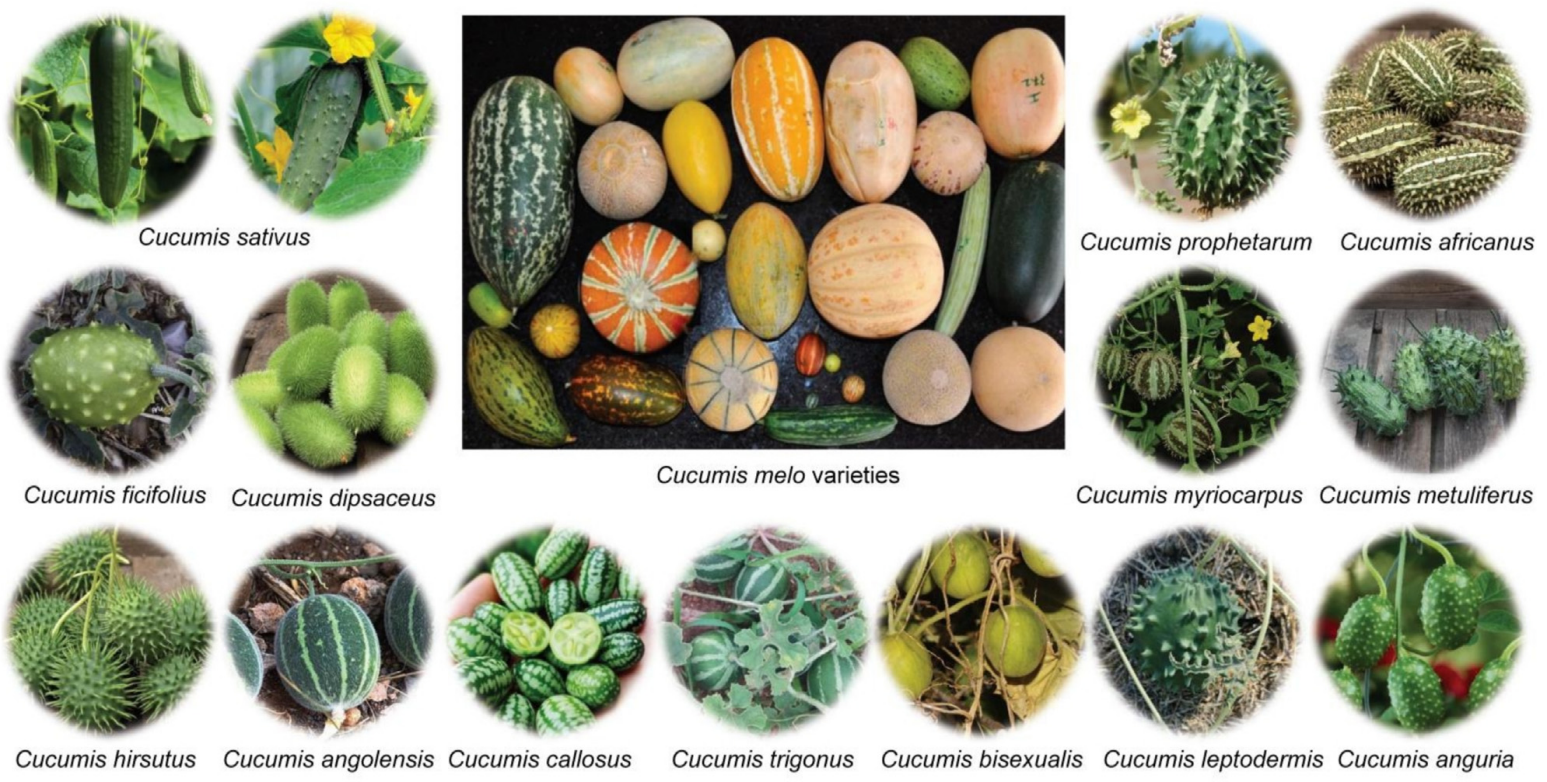
Morphology of representative species of the genus *Cucumis.* These botanical names follow the WFOPL (https://wfoplantlist.org/).

*Cucumis* has significant economic, nutritional, and medicinal value, with species used as food, oils, raw materials, and phytochemical sources. In the traditional Chinese, Indian, and African medicine, they are employed to treat gastrointestinal, metabolic, urogenital, hepatic, dermatological, cardiovascular, respiratory, inflammatory, and infectious disorders (Table 1). Two species dominate cultivation worldwide: *C. sativus* L. “cucumber”, domesticated in Asia, and *C. melo* L. “melon”, whose African versus Asian domestication remains debated. *C. sativus* (Fig. 1) ranks as the fourth most widely cultivated vegetable crop worldwide, consumed fresh, fermented (as pickles), or cooked, and represents the most economically significant species within the genus (Idemudia and Enogieru, 2024, Mukherjee et al., 2013). *C. melo* comprises highly diverse subspecies and varieties that, despite sharing a common genetic ancestry, exhibit marked divergence in agro-morphological and fruit sensory traits. This variability, driven by natural selection and human breeding practices, has resulted in the emergence of distinct cultivar-groups with unique metabolic and phenotypic characteristics. Taxonomically, *C. melo* varieties (Fig. 1) are classified into two subspecies distinguished by ovary hair morphology into *C. melo* subsp. *melo* and *C. melo* subsp. *agrestis*. The former includes *C. melo* var. *cantalupensis* Naudin, *C. melo* var. *reticulatus* Ser., *C. melo* var. *inodorus* H. Jacq., *C. melo* var. *flexuosus* (L.) Naudin, *C. melo* var. *utilissimus* (Roxb.) Duthie & Fuller, *C. melo* var. *dudaim* (L.) Naudin, and *C. melo* var. *chito* (C. Morren) Naudin. Meanwhile, the latter includes *C. melo* var. *agrestis* (Naudin) Pangalo, *C. melo* var. *momordica* (Roxb.) Cogn., *C. melo* var. *conomon* (Thunb.) Makino, *C. melo* var. *makuwa* Makino, *C. melo* var. *chinensis* Pangalo, and *C. melo* var. *acidulus* Naudin (Gómez-García et al., 2020, Lija and Beevy, 2021, Moing et al., 2020, Silva et al., 2020).

**Table 1:** Traditional uses of genus *Cucumis*, including ethnomedicinal applications and regions of use.

Species/Varieties (common names)	Traditional ethnomedicinal uses	Regions of use	References
*C. sativus* (Cucumber or Gherkin)	Fruits: used in culinary, nutritional, cosmetic preparations (soothing, hydrating, skin-healing, burns irritation), alcohol intoxication, sores, wrinkles, hyperpigmentation, and constipation; used as anti-diabetic, hepatoprotective, diuretic, antipyretic, anti-hemorrhoidal, anti-varicose, and gastric anti-inflammatory; used to promote digestion. Fruits/Peels: used for eye, bladder, and kidney disorders; used against scabies and itching. Seeds: used in dysuria, obstructive uropathy, bleeding disorders, debility, insomnia, headaches, vomiting, and fever; also, in spleen and liver enlargement; used as diuretic, cooling, litholytic, and deworming agent. Fruits/Seeds: used in urinary and GIT disorders; promoted heart health; acted as anti-urolithiatic. Leaves/Roots/Stems: used in diarrhea, dysentery; acted as anti-urolithiatic; used for urinary stones. Stems: used in gonorrhea, lupus, hypertension, and inflammation. Roots: used in urinary stones.	Brazil; Catalonia (Iberian Peninsula); China; Fiji; Iran; Mexico; Northern India; Pakistan; Russia	Gras et al., 2019, Kasote et al., 2017, Mukherjee et al., 2013, Mukherjee et al., 2022, Olisova et al., 2018, Parvinroo et al., 2014, Roman-Ramos et al., 1995, Tang et al., 2010, Wahid et al., 2022, Yang and Walters, 1992
*C. melo* (Melon)	Fruits: used in fever, cough, toothache, constipation, diabetes, edema, dysuria, leucorrhea, and food poisoning; also, in urological and urogenital diseases; applied for burns, abrasions, and eczema; used as galactagogue, diaphoretic, digestive, purgative, and diuretic. Pulp: used as diuretic, anthelmintic; also, as lotion for acute and chronic eczema. Seeds: used in infectious hepatitis, appendicitis, pulmonary abscesses, eczema, and hematoma; also, in renal and bladder disorders (stones, dysuria, urolithiasis, ulcers, anuria), jaundice, liver and kidney inflammation, vitiligo, ascites, chronic fevers, and debility; used as digestive, febrifuge, anti-tussive, vermifuge, lithotriptic, laxative, cooling, demulcent, and diuretic; also as anti-diabetic remedy and for fertility regulation. Leaves: used in hematoma and eczema. Stems: used in dysentery, hypertension, dyspepsia, jaundice, liver inflammation, cirrhosis, hematoma, and cancer. Pedicels: used as rectal suppositories for abdominal distention and constipation; orally for expectoration, edema, jaundice, cirrhosis, liver cancer, viral hepatitis, hypertension, anasarca, and indigestion. Roots: used as emetic.	China; Ethiopia; India; Pakistan	Afzal et al., 2021, Feyisa et al., 2022, Gao et al., 2012, Ibrahim et al., 2016, Lal and Lata, 1980, Mukherjee et al., 2022, Piao et al., 2018, Umair et al., 2019, Yang and Walters, 1992, Yuan et al., 2019
*C. melo* var. *cantalupensis* (Cantaloupe)	Fruits/Seeds: used in hepatic inflammation, cough, eczema, and toothache; also, in urinary tract ulcers and kidney disorders; used as diuretic.	China	Ezzat et al., 2019, Gopalasatheeskumar et al., 2020
*C. melo* var. *reticulatus* (Muskmelon)	Fruits/Seeds: used in hepatic inflammation, cough, eczema, and toothache; also, in urinary tract ulcers and kidney disorders; used as diuretic.	China	Ezzat et al., 2019, Gopalasatheeskumar et al., 2020
*C. melo* var. *inodorus* (Honeydew)	Fruits: used as digestive, febrifuge, anti-tussive, demulcent, and vermifuge. Seeds: used in urinary stones.	China	Barghout et al., 2024, Eidi and Ashjazadeh, 2023
*C. melo* var. *flexuosus* (Snake melon, Serpent melon, or Armenian cucumber)	Whole plant: used in diabetes, cough, jaundice, stomach pain, and intestinal inflammation; acted as galactagogue, emmenagogue, lithotriptic, aphrodisiac, emetic, colonic, anti-diabetic, and anti-diarrheal. Seeds: used in hepatitis, appendicitis, and pulmonary abscesses. Stems: used in hepatic inflammation.	Asia; China; Iraq (South Kurdistan)	Ahmed, 2016, Shabab et al., 2021, Yang and Walters, 1992
*C. melo* var. *dudaim* (Queen Anne’s pocket melon)	Fruits: used as ornamental and decorative plant.	China	Yang & Walters, 1992
*C. melo* var. *agrestis* (Wild melon, Native gooseberry, or Kachri)	Fruits/Leaves: used as purgative, emetic, and remedy for indigestion. Leaves: used in diabetic diet.	India; Pakistan	Dwivedi et al., 2010, Gopalasatheeskumar et al., 2020, Yang and Walters, 1992
*C. melo* var. *conomon* (Katsura-uri or Japanese pickling melon)	Fruits: used as antidote and diuretic. Stems: used in hepatic inflammation. Seeds: used in hepatitis, appendicitis, and pulmonary abscesses.	China	Yang & Walters, 1992
*C. bisexualis* (Mapao egg or Muskmelon egg)	Fruits: used in diabetes and hypochondriac pain.	China	Ma et al., 2018, Ma et al., 2021
*C. prophetarum* (Globe cucumber, wild cucumber, Wild gourd, or Shari-al-deeb)	Fruits: used in rabies, labor induction, and liver disorders (anti-hepatotoxic); used as abortifacient. Fruits/Roots/Seeds: used in stomach pain and sexually transmitted diseases (STDs); employed as emetic and purgative. Leaves/Immature fruits: used as emetic and purgative; applied for conception and inflammatory conditions. Roots: used in fever, earache, lung disorders, heart failure, back pain, skin infections, diarrhea, gonorrhea, and other urogenital problems.	Ethiopia (Southern, Central regions); India (Aravalli ranges); Pakistan (Karachi); Saudi Arabia	Al-Rehaily et al., 2002, Ayele, 2018, Bussmann et al., 2020, Dwivedi et al., 2010, Feyisa et al., 2022, Galma et al., 2021, Kavishankar and Lakshmidevi, 2014, Olarewaju et al., 2021, Tamiru et al., 2019
*C. callosus* (Bitter cucumber)	Fruits: used in urinary irritations and dribbling, stomach pain, skin diseases, jaundice, fever, vomiting, constipation, sciatica, edema, cerebral congestion, colic spasms, and vertigo; used as cooling, appetite stimulant, bowel relief, anthelmintic, anti-tussive, anti-diabetic, diuretic, and memory enhancer. Pulp: used as bitter, thermogenic, anthelmintic, hepato-tonic, cardiotonic, expectorant, and intellect promoter. Pericarp: used in epilepsy, diarrhea, and diabetes. Seeds: used in bilious abnormalities, intestinal disorders, epilepsy, and vertigo; used as astringent, cooling, memory enhancer, and hair blackening. Leaves: applied as topical paste for lice, snake bites, and wound-healing; used as purgative and emetic. Roots: used in snake bites, abdominal enlargement, and urinary tract disorders; used as purgative, emetic, anti-inflammatory, anti-asthmatic, anti-rheumatic, anti-cough, and anti-ulcer.	Northwestern India; Sri Lanka	Choudhary et al., 2023, Deepika et al., 2023
*C. africanus* (Wild cucumber or Wild gherkin)	Whole plant: used in obesity, wound healing, skin infections, cancer, viral hepatitis, tuberculosis, gonorrhea, and other STDs. Fruits/Seeds/Leaves/Roots: used as emetic, purgative, cleanser, and laxative; used in parasitic infections and enemas. Leaves: used in animal ailments, malaria, skin infections, pain, and inflammation.	Namibia; South Africa	Abifarin et al., 2019, Afolayan and Mbaebie, 2010, Olarewaju et al., 2021, Omokhua-Uyi and Van Staden, 2020, Stafford et al., 2008
*C. ficifolius* (Wild melon)	Fruits: used in anthrax, toothache, inflammation, tonsillitis, wounds, joint pain, vomiting, asthma, eczema, retained placenta, birth control, eye injuries, tetanus, gonorrhea, malaria, ear infections, tuberculosis, coccidiosis, and abdominal pain; also, in bites of snakes, scorpions, and black spiders. Roots: used in collapse, hepatic and urogenital diseases, meningitis, epistaxis, burns, chest pain, stomachache, diarrhea, rabies, and amoebas.	Ethiopia	Araya et al., 2019, Bizuneh et al., 2023, Bussmann et al., 2020, Demsie et al., 2019, Feyisa et al., 2022, Khanal et al., 2021, Meragiaw et al., 2016, Teklehaymanot and Giday, 2007, Teklehaymanot et al., 2007
*C. metuliferus* (African horned cucumber, Jelly melon, or Kiwano)	Fruits/Pulp: used in peptic ulcers, hepatitis, jaundice, diabetes, hypertension, HIV/AIDS, and male fertility problems; also used during the bird flu (avian influenza) outbreaks. Seeds: used to expel intestinal worms. Roots: used in postpartum pain relief and asthma. Leaves: used in asthma and exhaustion; used as laxative and topical anti-inflammatory.	Nigeria (Plateau State); South Africa (Vhembe district)	Manjunathagowda et al., 2023, Olarewaju et al., 2021, Omokhua-Uyi and Van Staden, 2020, Šeregelj et al., 2022
*C. dipsaceus* (Teasel gourd, Arabian cucumber, or Hedgehog)	Fruits: used in cancer, meningitis, hepatitis, and GIT, reproductive, and urogenital disorders, dandruff, and hair care; used as antidote, purgative, childbirth aid, demulcent; used for weaning infants. Leaves: used in snake and fox bites, cough, diuretic, hemorrhoids, rabies, dandruff; used as childbirth aid and wound poultices; used for weaning infants. Roots: used in hepatitis, snake bites, gallstones, and childbirth; used as wound poultices. Seeds: used as diuretic.	Ethiopia; Kenya; Northern Peru; Tanzania	Asefa and Nedi, 2024, Assefa et al., 2024, Bussmann and Glenn, 2010, Bussmann et al., 2020, Feyisa et al., 2022, Khanal et al., 2021, Olarewaju et al., 2021, Teklehaymanot et al., 2007
*C. myriocarpus* (Gooseberry gourd, Gooseberry cucumber, Paddy melon, or Prickly paddy melon)	Leaves: used in STDs (syphilis, gonorrhea). Tuber/Pulp: used in deworming and for skin boils; used as purgative.	Africa	Omokhua-Uyi and Van Staden, 2020, Semenya et al., 2013, Shaik et al., 2017
*C. leptodermis*	Fruits: used in STDs (syphilis, gonorrhea).	South Africa (Limpopo province)	Olarewaju et al., 2021, Semenya et al., 2013
*C. hirsutus* (Wild cucumber)	Leaves/Roots: used in GIT and respiratory ailments, female infertility, vomiting, fontanel syndrome, and convulsions.	Mozambique; Zimbabwe	Fawole et al., 2009, Olarewaju et al., 2021, Sitoe and Van Wyk, 2024, Stafford et al., 2008
*C. anguria* (West Indian gherkin or Bur gherkin)	Fruits: used in diabetes, cough, edema, stomach problems, wounds, boils, and kidney stones. Fruits/Seeds: used in hemorrhoids, worms, and enemas. Leaves: used for freckles and wounds.	Brazil; Colombia; Cuba; South Africa	Omokhua-Uyi and Van Staden, 2020, Ribeiro et al., 2023
*C. trigonus* (Kachri)	Fruits: used in leprosy, fever, jaundice, diabetes, cough, bronchitis, anemia, constipation, and GIT disorders; used as thermogenic, febrifuge, expectorant, liver tonic, anthelmintic, diuretic, stomachic, appetizer, purgative, intellect promoter, anabolic, analgesic, and anti-inflammatory.	India	Kumar et al., 2020, Salahuddin and Jalalpure, 2010, Wright et al., 2007
*C. zeyheri* (Wild cucumber)	Fruits/Seeds/Roots: used as purgative; used for constipation relief.	South Africa	Omokhua-Uyi & Van Staden, 2020
*C. aculeatus* (Kisawasawa)	Fruits: used in malaria and general weakness. Leaves: used in malaria, diarrhea, leprosy, migraines, wounds, and gonorrhea.	Africa (mountain regions); Himalayas; Kenya (Luhya community)	Bussmann et al., 2020, Khanal et al., 2021, Mukungu et al., 2016
*C. hardwickii*	Roots: used in fever and urinary disorders.	Western Himalayas (foothills of Nepal)	Khanal et al., 2021, Malik et al., 2015
*C. pubescens*	Fruits (ripe/unripe): used as laxative, diuretic, galactagogue, and diaphoretic; used to strengthen heart and brain; used in ophthalmia and urinary discharges.	India	Sundari & Kavitha, 2024

Of the 60 accepted species, 18 are recognized for their traditional ethnomedicinal uses, including *C. sativus* L., *C. melo* L., *C. bisexualis* A. M. Lu & G. C. Wang, *C. prophetarum* L., *C. callosus* (Rottler) Cogn., *C. africanus* L. f., *C. ficifolius* A. Rich., *C. metuliferus* E. Mey. ex Naudin, *C. dipsaceus* Ehrenb. ex Spach, *C. myriocarpus* Naudin, *C. leptodermis* Schweick., *C. hirsutus* Sond., *C. anguria* L., *C. trigonus* Roxb., *C. zeyheri* Sond., *C. aculeatus* Cogn., *C. hardwickii* Royle, and *C. pubescens* Willd. (Table 1, Fig. 1). However, only 15 have been chemically characterized, 12 evaluated inconsistently for biological activities, two subjected to clinical studies, and ten implicated in toxicological aspects.

To date, a total of 428 secondary metabolites (excluding volatiles and fatty acids) from diverse phytochemical classes, including steroids, triterpenoids, flavonoids, coumarins and other phenolics, polysaccharides, and other phytoconstituents, have been isolated from *Cucumis* species (Table S1). Crude extracts, fractions, and isolated metabolites exhibit a wide range of pharmacological activities, such as anti-inflammatory, antihypertensive, anti-diabetic, anti-cancer, organ-protective, antimicrobial, and antiviral properties, mainly attributed to cucurbitacins and phenolics, supporting traditional uses. Although clinical evidence is limited, the genus exhibits potential in managing metabolic disorders, osteoarthritis, and nephrolithiasis, and promoting skin health and cosmetic applications. Despite these promising findings, some species have been reported as toxic, largely due to cucurbitacins, which can cause gastrointestinal, hepatic, and systemic toxicity, underscoring the need for cautious use and rigorous toxicological evaluation.

To the best of our knowledge, only a limited number of reviews have addressed the ethnomedicinal applications, phytochemical profiling, and pharmacological activities of *Cucumis* species, and one of them has been written in Portuguese (Da Silva et al., 2023, Insanu et al., 2022). A few additional reviews have focused on individual species, particularly those of significant agricultural or medicinal value. Although research on these species has expanded over the past decade, comprehensive analyses of their secondary metabolites and biological functions remain sparse. Notably, previous reviews have largely overlooked the clinical applications and toxicological profiles of the genus, highlighting a critical gap in the current literature.

This review, therefore, provides a comprehensive summary of the research progress on the traditional ethnomedicinal uses, phytochemistry, pharmacology, clinical applications, and toxicological profiles of the genus *Cucumis* over recent decades. A primary objective is to identify and critically highlight the existing gaps in the literature, thereby guiding and prioritizing future investigations aimed at unlocking the full medicinal potential of *Cucumis* species.

## 2 Research methodology

A comprehensive literature search on *Cucumis* species was conducted without language restrictions, covering publications from 1933 to December 2025. Data were retrieved from major scientific databases, including Web of Science, Scopus, PubMed, ScienceDirect, Google Scholar, Elsevier, Springer, Wiley, Frontiers, Taylor & Francis, ResearchGate, ClinicalKey, and EKB (Egyptian Knowledge Bank), as well as CNKI (China National Knowledge Infrastructure) for Chinese literature. Keywords included the genus and all species names, along with “traditional uses”, “ethnomedicine”, “phytochemistry”, “pharmacology”, “clinical studies”, “toxicity”, and “poisoning”. Botanical nomenclature was verified using WFO and WFOPL databases. Some data on ethnobotanical uses were sourced from the Useful Tropical Plants database (https://tropical.theferns.info), while information on species’ geographical distribution was obtained from the GBIF website. All chemical structures were drawn using ChemDraw Professional 23.1.1 and validated for stereochemistry and configurations via Reaxys and PubChem databases.

## 3 Traditional uses and geographical distribution of *Cucumis* species

*Cucumis* species are widely distributed across Africa, Asia, Australia, and some islands in the Pacific, with cultivation dating back to Ancient Egypt (Šeregelj et al., 2022). Two major centers of diversity are recognized: (i) Africa, which hosts the largest number of species, and (ii) Asia, particularly south and east of the Himalayas (Table 1). Some species were later introduced to the Americas, Europe, and Oceania, reflecting their broad geographic dissemination (Chen & Zhou, 2011), according to the WFO, WFOPL, and GBIF websites. Although the genus comprises a diverse range of species, with 60 currently accepted, yet traditional ethnomedicinal uses have been documented for only 18 species and several varieties across regions such as China, India (Ayurveda and Unani), Africa, and South America (Table 1). These species have historically been employed for the management of gastrointestinal, metabolic, urogenital, hepatic, dermatological, cardiovascular, respiratory, inflammatory, and infectious disorders. Table 1 provides a concise summary of the ethnomedicinal applications, plant parts used, and regions of documented use for *Cucumis* species in the literature.

Among them, *C. sativus*, one of the most widely distributed and extensively utilized species, has been cultivated for over 3 000 years and features prominently in the Chinese, Indian (Ayurveda and Unani), Iranian, Brazilian, Russian, and Mexican traditional practices. It is employed for the management of fever, jaundice, leprosy, diabetes, and respiratory conditions, and is highly valued in dermatological applications, including skin lightening, anti-aging, and wound healing (Idemudia and Enogieru, 2024, Khan et al., 2022, Wahid et al., 2022, Yang and Walters, 1992). Similarly, *C. melo* holds a prominent role in traditional Chinese medicine, where the dried pedicels (*Pedicellus Melo*, “Tian Gua Di”) are used as decoctions or suppositories for febrile and gastrointestinal tract (GIT) disorders (Gao et al., 2012). In Ayurveda and Unani medicine, its fruits and leaves are employed to treat diabetes, urinary complications, and skin infections. Its ethnomedicinal applications also extend to South Asia (Pakistan, India, Sri Lanka), South Kurdistan, and Saudi Arabia, where *C. melo* is used against digestive, inflammatory, infectious diseases, etc. (Table 1) (Ahmed, 2016, Mukherjee et al., 2022).

In Sub-Saharan Africa, *Cucumis* species are deeply integrated into local healing practices. In Ethiopia, Mozambique, South Africa, Kenya, and Tanzania, decoctions of leaves, roots, or whole plants are used against helminthic infections, malaria, GIT disorders, and even cancer. Topical preparations, including pastes, poultices, crushed leaves, or fruit juice, are applied for wounds, skin infections, and inflammation. In Nigeria and the Limpopo province of South Africa, traditional healers utilize formulations for systemic illnesses, while in Colombia and Peru, the fruits and roots are administered as antidotes to poisoning (Table 1) (Manjunathagowda et al., 2023, Olarewaju et al., 2021, Semenya et al., 2013, Stafford et al., 2008). Collectively, these examples demonstrate that *Cucumis* species are not only widely distributed but also deeply embedded in regional ethnomedicinal systems, reflecting their therapeutic versatility. Despite the wide range of traditional uses, the pharmacological validation is limited. Moreover, the active principles and mechanisms of action remain poorly understood, emphasizing the need for further scientific investigation to bridge traditional knowledge and evidence-based medicine.

## 4 Phytochemistry of *Cucumis* species

**Fig. 2:**
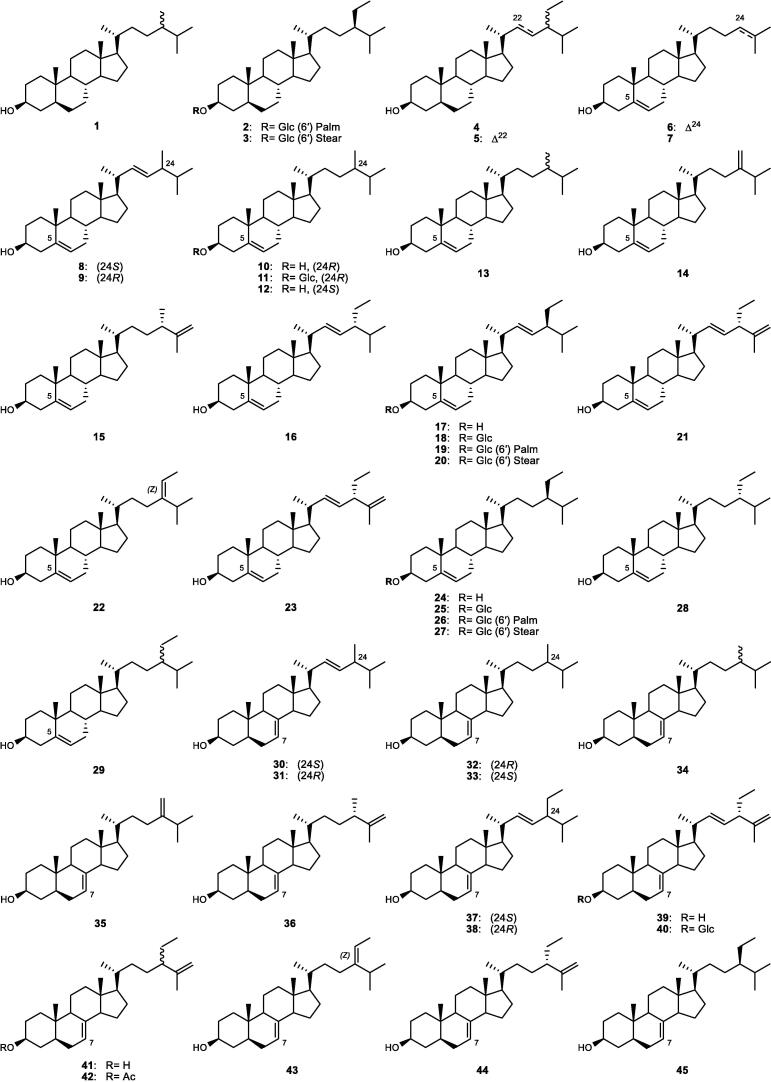

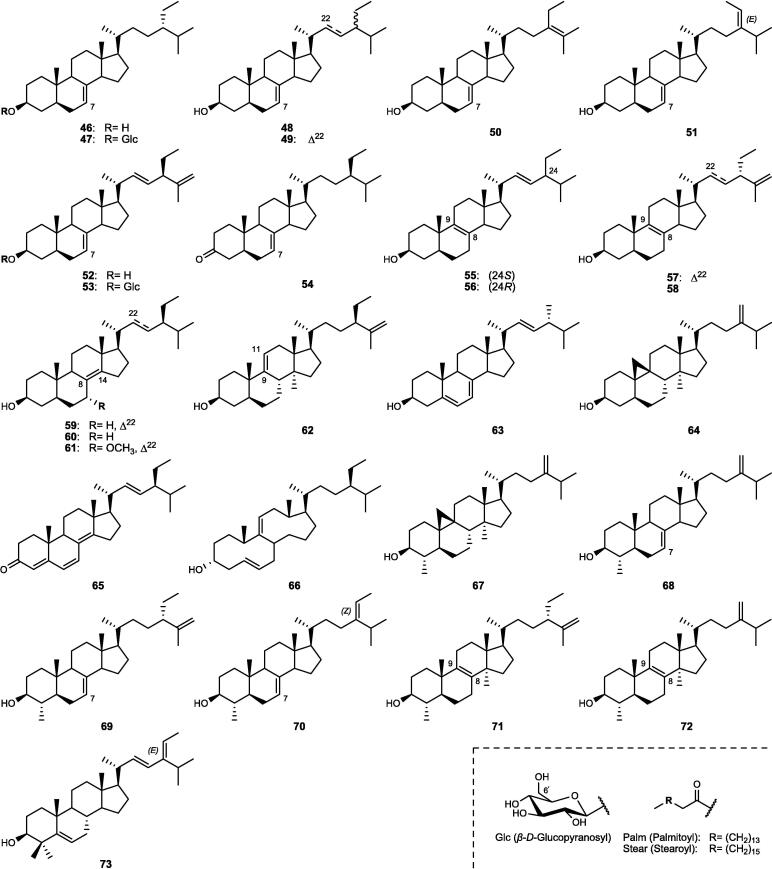
Steroids (**1** − **45**) isolated from genus *Cucumis*.

**Fig. 3:**
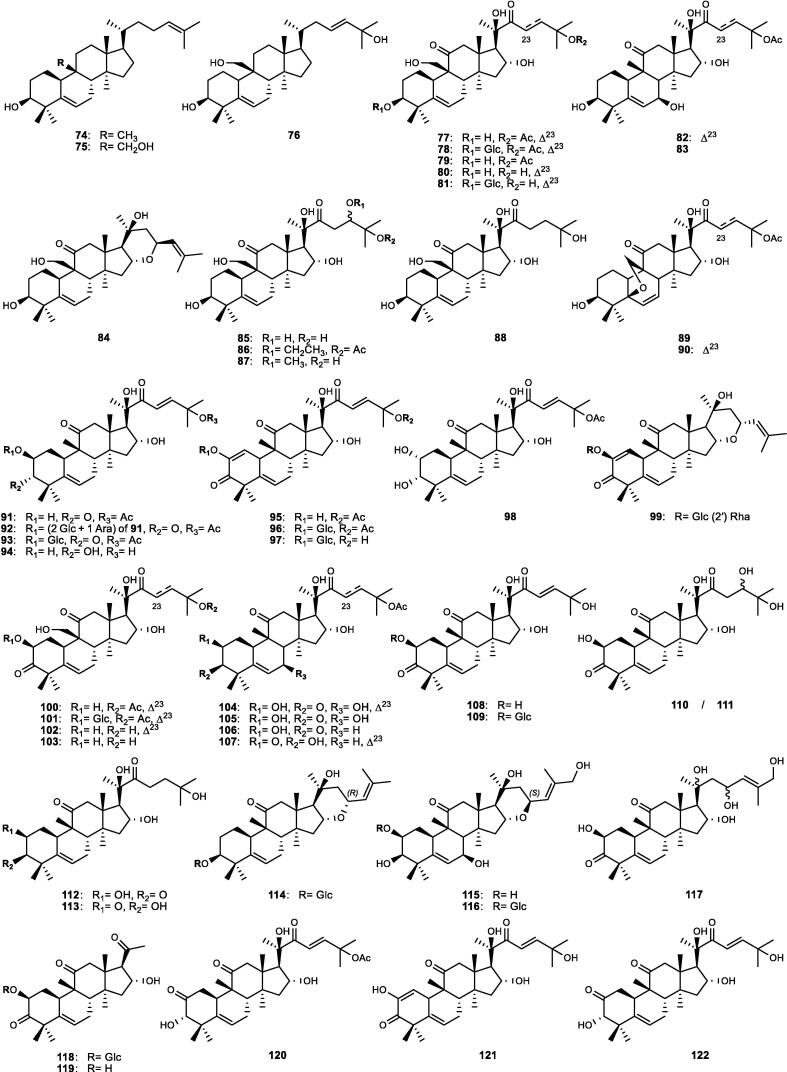

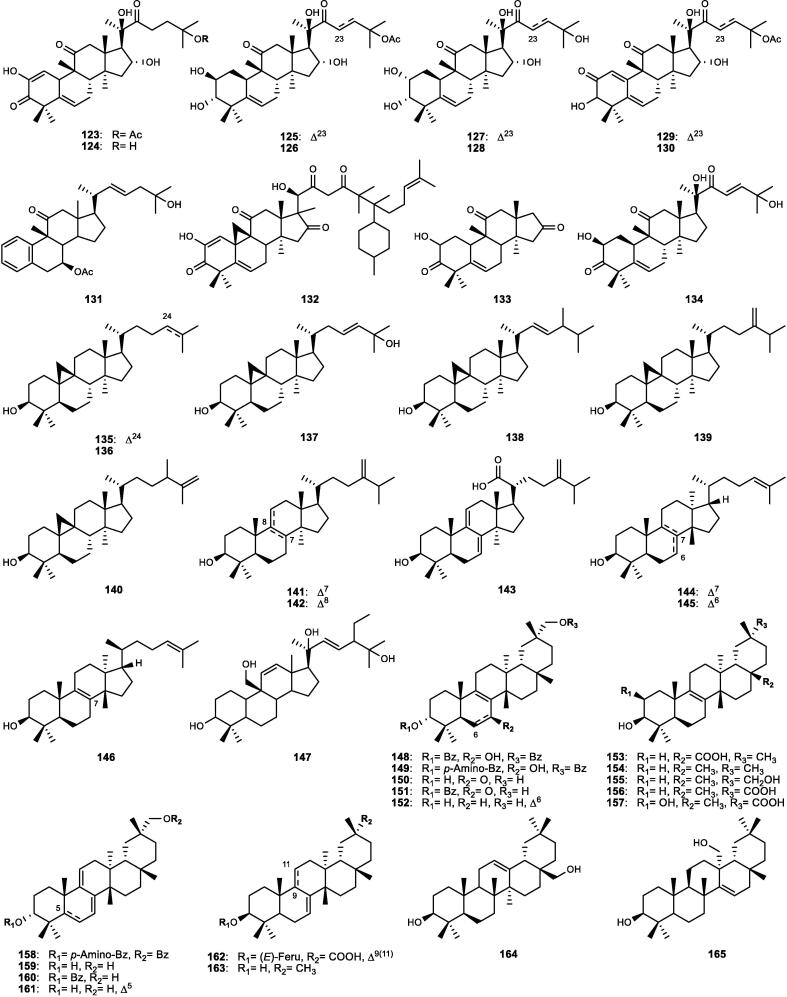

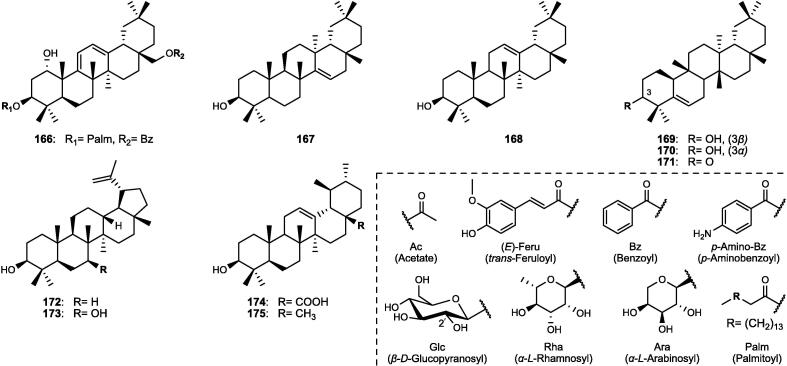
Triterpenoids (**74** − **122**) isolated from genus *Cucumis*.

**Fig. 4:**
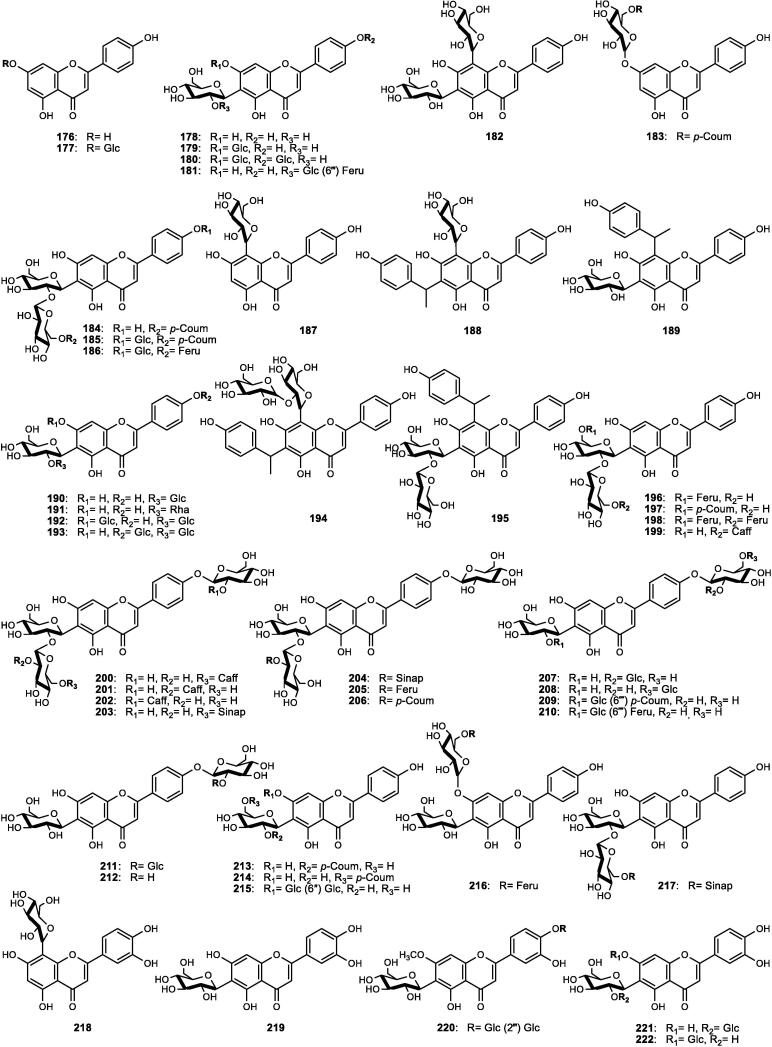

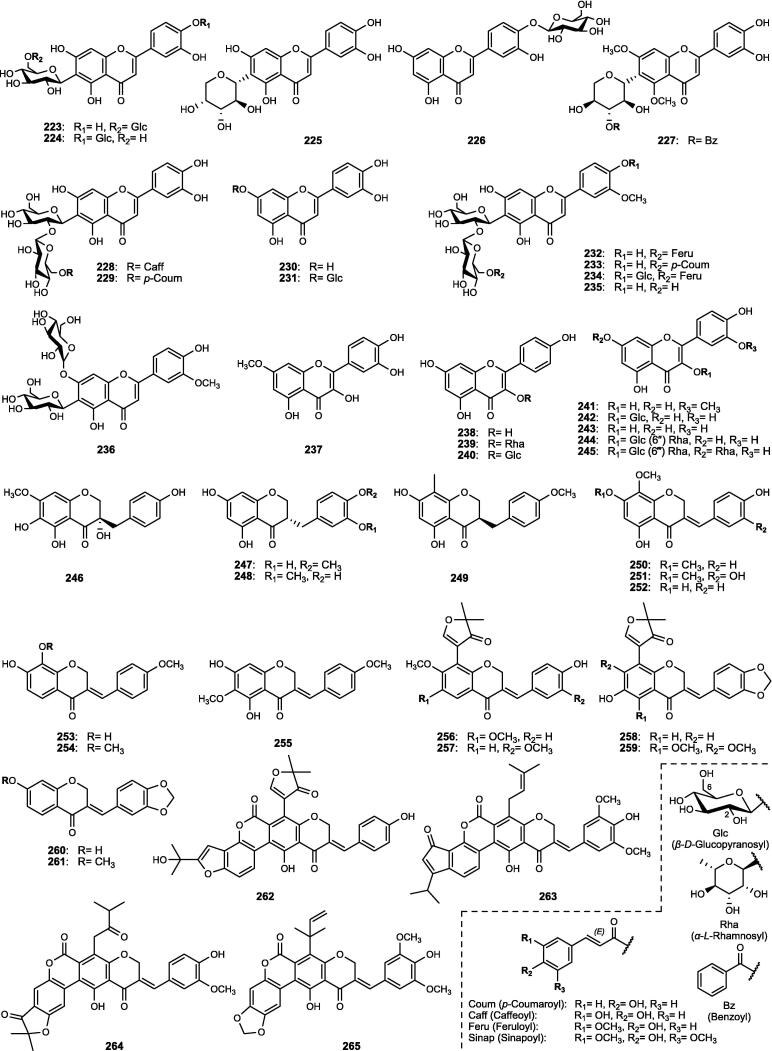
Flavonoids (**176** − **222**) isolated from genus *Cucumis*.

**Fig. 5:**
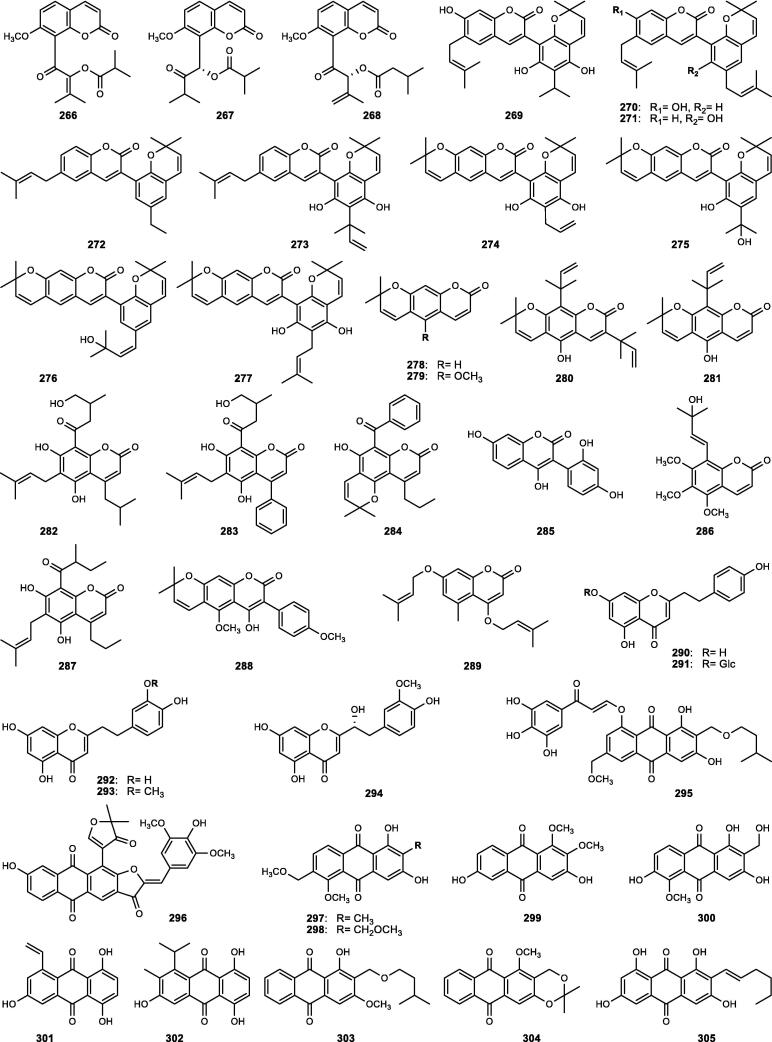

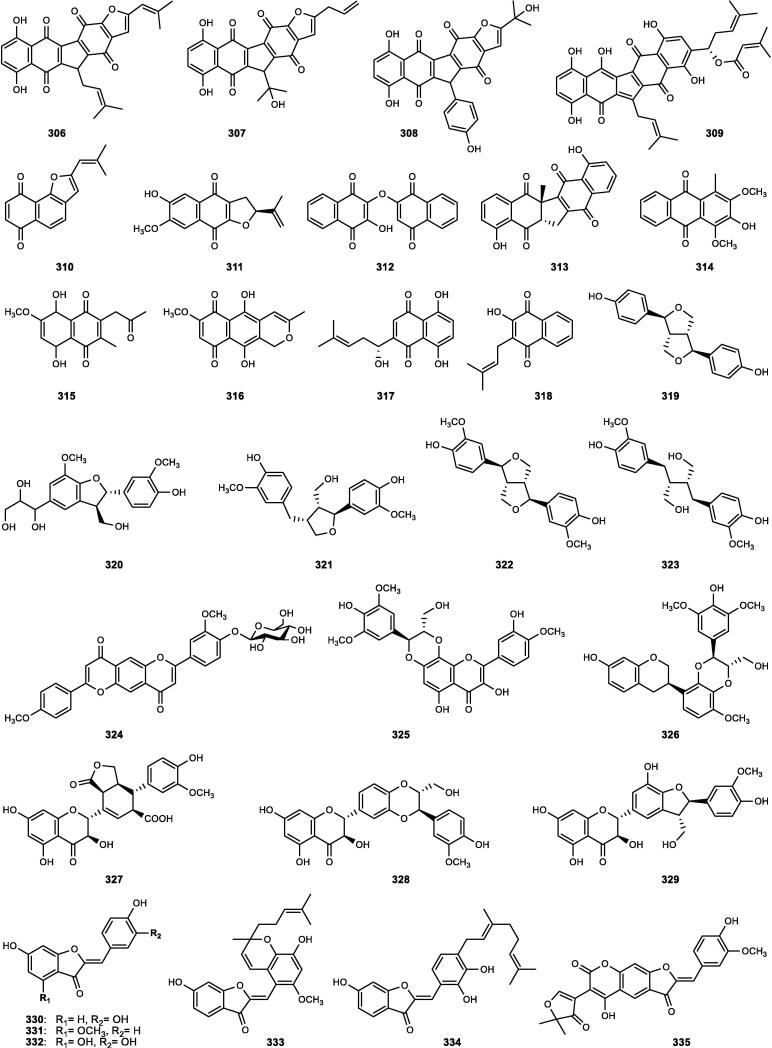

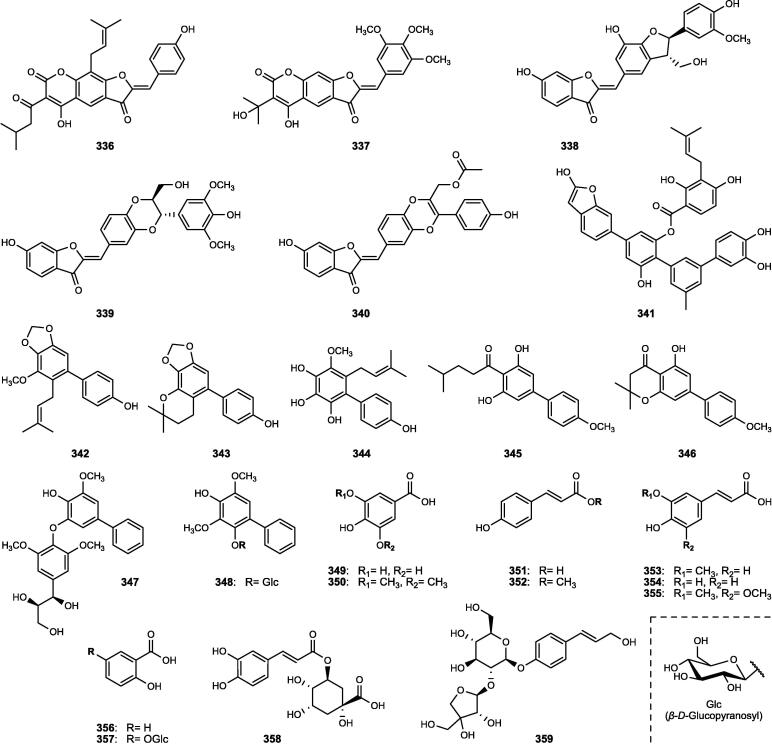
Coumarins and other phenolics (**266** − **305**) isolated from genus *Cucumis*.

**Fig. 6:**
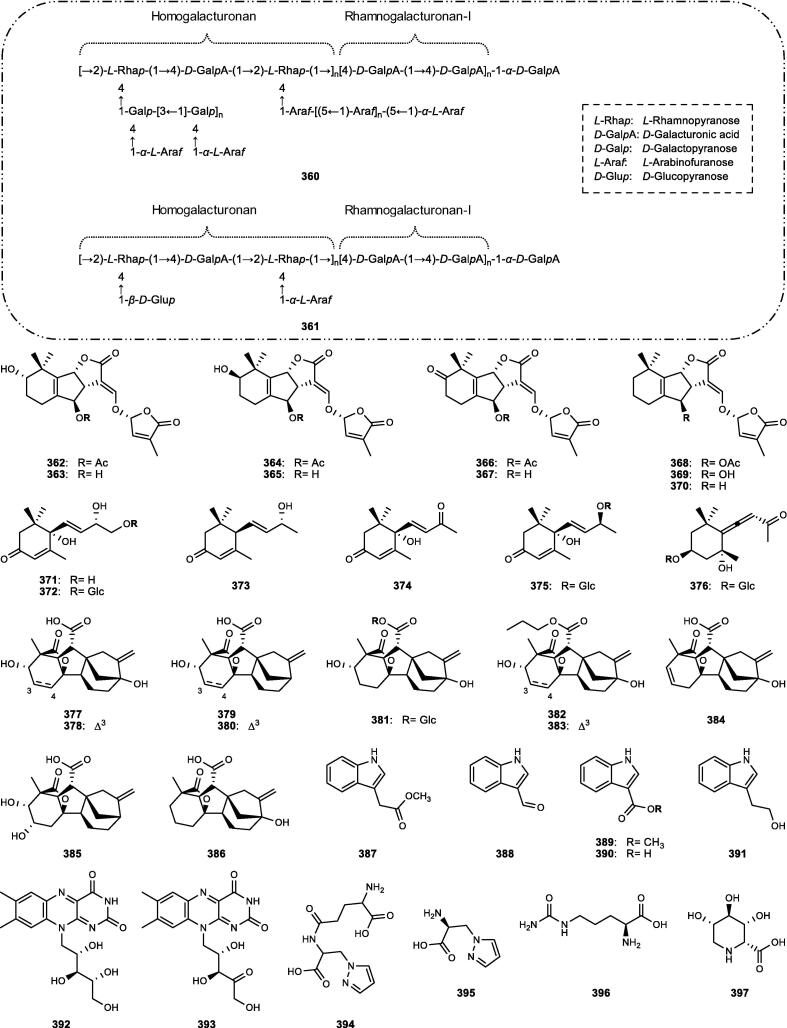

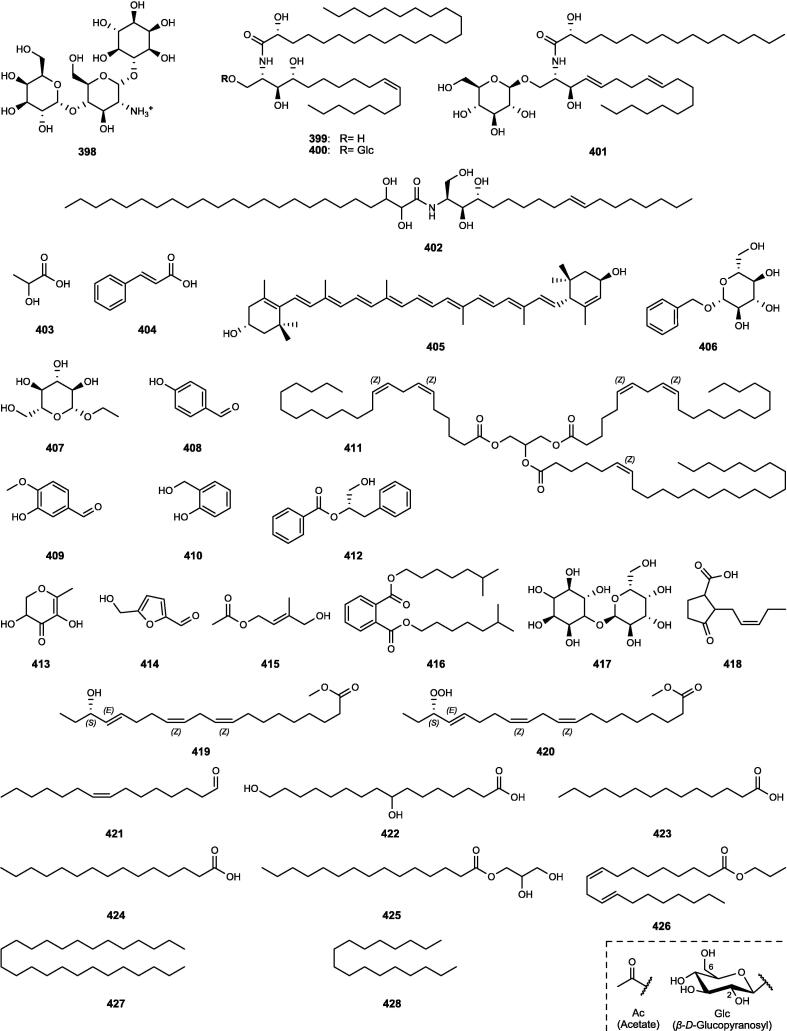
Other compounds (**360** − **397**) isolated from genus *Cucumis*.

Although the genus *Cucumis* includes about 60 accepted species, only 15 have been phytochemically investigated, including *C. sativus*, *C. melo*, *C. bisexualis*, and *C. prophetarum*. To date, 428 secondary metabolites (excluding volatile oils) have been identified using different extraction and spectroscopic techniques. The secondary metabolites identified from the genus can be broadly classified into five major groups: steroids (**1** − **73**, Fig. 2), triterpenoids (**74** − **175**, Fig. 3), flavonoids (**176** − **265**, Fig. 4), coumarins and other phenolics (**266** − **359**, Fig. 5), volatile oils, and other compounds (**360** − **428**, Fig. 6). A comprehensive overview of these metabolites, including their nomenclature, sources, and literature references, is presented in Table S1. Since most species remain unexplored, further research could reveal novel bioactive compounds of therapeutic relevance.

### 4.1 Steroids

Phytochemical studies of *Cucumis* species revealed a diverse array of sterols (**1** − **73**) with C_27_ − C_31_ skeletons, mainly isolated from the seeds and aerial parts of *C. sativus* and *C. melo*, as shown in Table S1 and Fig. 2. They were classified according to the presence of a methyl group at C-4 into desmethylsterols (cholestane group, **1** − **66**) and 4*α*-methylsterols (**67** − **73**). The cholestane group (**1** − **66**) was further subdivided based on double-bond types into saturated (**1** − **5**), Δ^5^- (**6** − **29**), Δ^7^- (**30** − **54**), Δ^8^- (**55** − **58**), Δ^8(14)^- (**59** − **61**), 14*α*-methyl-Δ^9(11)^- (**62**), Δ^5,7^- (**63**), 14*α*-methyl-9*β*,19-cyclo- (**64**), 3-oxo-Δ^4,6,8(14)^- (**65**), and the novel *diseco*-Δ^5,9(11)^- (**66**) skeletons with a variety of side chains (Akjhisa et al., 1987; Akihisa et al., 1986, Akihisa et al., 1986, Garg and Nes, 1986, Matsumoto et al., 1983a, Matsumoto et al., 1983b).

The 24-alkyl-Δ^7^-sterols (e.g., **36**, **38** − **39**, **44** − **46**) were predominant in the seeds and mature tissues of *C. sativus* and *C. melo*, whereas minor or unusual Δ^8^-sterols (**55** − **58**) and rare skeletons (e.g., **59**, **62**, **64**) were detected in the aerial parts (Akjhisa et al., 1987, Akihisa et al., 1986). In addition, the 4*α*-methylcholestane sterols (**67** − **72**) were isolated from *C. sativus* seeds, while the roots of *C. prophetarum* yielded a novel 4,4-dimethyl derivative (**73**) structurally related to stigmasterol (Tamiru, Temesegen, & Demise, 2019). Sterols were also identified in the flowers, fruits, roots, and stems (Knights & Smith, 1977), as summarized in Table S1. Furthermore, distinct sterols were reported across different *Cucumis* species, including glucosylsterols (**11**, **18**, **25**) and chondrillasterol (**38**) in *C. callosus*, *α*-spinasterol (**36**) in *C. ficifolius*, and sterols (**23**, **25**, **36**) along with the novel *diseco*-Δ^5,9(11)^ sterol (**66**) in *C. prophetarum* (Al-Rehaily, Al-Yahya, Mirza, & Ahmed, 2002).

### 4.2 Triterpenoids

Triterpenoids are widely distributed in *Cucumis* species and represent an important class of bioactive metabolites. As listed in Table S1 and Fig. 3, they comprise tetracyclic (**74** − **147**) and pentacyclic (**148** − **175**) derivatives, which have been isolated or detected using advanced spectroscopic techniques. Triterpenoid tetracyclic derivatives (**74** − **147**) were further divided into 61 cucurbitane-type compounds (**74** − **134**) and 13 additional derivatives (**135** − **147**).

#### 4.2.1 Tetracyclic triterpenoids (cucurbitane-type)

Cucurbitane-type compounds (**74** − **134**), commonly known as “cucurbitacins”, are highly oxygenated tetracyclic triterpenoids characterized by the cucurbitane skeleton “19-(10 → 9*β*)-*abeo*-10*α*-lanost-5-ene”, occurring in both free and glycosidic forms in *Cucumis* species (Table S1, Fig. 3). They are regarded as bitter principles and chemotaxonomic markers of the genus (Chen, Chiu, Nie, Cordell, & Qiu, 2005). Several cucurbitacins (A − I and O − R), along with their glycosides and dihydro-/iso-derivatives, have been identified (Rehm, Enslin, Meeuse, & Wessels, 1957). Cucurbitacin C (**77**) is the major bitter compound, detected mainly in the leaves and stems of specific cultivars of *C. sativus* such as Hanzil and SJ6101 (Mukherjee et al., 2013, Shang et al., 2014). Advanced analytical techniques further revealed multiple cucurbitacin C-type derivatives (**78** − **83**) and novel cucurbitacins, including cucurbitacins C_1_ − C_7_ (**84** − **90**) (Chen et al., 2022, Qing et al., 2022). Cucurbitacin B (**91**) dominates in *C. melo*, particularly in the roots, fruits, and pedicels, representing up to 62% of total cucurbitacins, followed by cucurbitacins E (**95**) and D (**108**) (Yuan et al., 2019). Several novel cucurbitane-type derivatives within the range of compounds **100** − **121**, including cucurbitacins A, B, and D derivatives, were isolated from the stems, pedicels, and fruits (Table S1, Fig. 3).

Likewise, *C. prophetarum* and related taxa accumulated cucurbitacins B (**91**), D (**108**), G (**110**), and H (**111**), and other derivatives (Table S1), including newly identified cucurbitacins (**110** − **113**, **119** − **133**) from the fruits and roots (Chen et al., 2005, Galma et al., 2021, Rehm et al., 1957, Tamiru et al., 2019). In other species such as *C. callosus*, *C. dipsaceus*, *C. africanus*, and *C. anguria*, cucurbitacins B (**91**), D (**108**), Q_1_ (**125**), and several minor derivatives (Table S1) were recorded, with the fruits and roots being the richest organs (Abd El-Fattah et al., 1989, Assefa et al., 2024, Maja et al., 2022, Ul Haq et al., 2019). It is worth mentioning that the distribution of cucurbitacins A − H among 17 African *Cucumis* species (listed in Table S2) shows marked interspecific variation, with the fruits, roots, and leaves serving as the principal reservoirs. For example, cucurbitacin A (**100**), restricted to this genus, occurs abundantly in *C. myriocarpus* and *C. leptodermis*, where its co-occurrence with minor cucurbitacins B (**91**) and D (**108**) highlights their close relationship. Conversely, cucurbitacins B (**91**) and D (**108**) dominate in *C. ficifolius*, while cucurbitacins D (**108**) and F (**94**) impart the characteristic bitterness of *C. angolensis* and *C. dinteri* (Miró, 1995, Rehm et al., 1957).

#### 4.2.2 Other tetracyclic triterpenoids

Besides cucurbitacins, other tetracyclic triterpenoids, derived from the lanostane (**135** − **143**), euphane (**144** − **145**), and tirucallane (**146**) types, have been reported (Table S1, Fig. 3). They were mainly isolated from the seeds of *C. sativus* and *C. melo*, while some lanostane derivatives (e.g., **138**, **143**) were also obtained from the stems and roots (Zhou et al., 2012). An unusual tetracyclic triterpenoid (**147**) was further isolated from the fruits of *C. dipsaceus* (Assefa et al., 2024).

#### 4.2.3 Pentacyclic triterpenoids

A total of 28 pentacyclic triterpenoids (**148** − **175**) have been described in *Cucumis* species (Table S1, Fig. 3). The seeds of *C. sativus* and *C. melo* are rich in friedooleanane-type triterpenes as dominant components such as isomultiflorenol (**154**) and multiflorenol (**163**), while mature plant parts contain common triterpene alcohols such as *α*-(**174**) and *β*-(**168**) amyrins (Akihisa et al., 1988, Akihisa et al., 1997, Itoh et al., 1982, Kintia and Wojciechowski, 1975). The roots of *C. sativus* yield unique metabolites including bryonolic acid (**156**), 2*β*-hydroxybryonolic acid (**157**), and 3*β*-bryoferulic acid (**162**), some of which were associated with mycorrhizal symbiosis (Zhou et al., 2012). Bryonolic acid (**156**), an acidic triterpene of friedooleanane-type, was reported in significant amounts in the roots, seedlings, and callus cultures of different *C*. *sativus* and *C*. *melo* varieties (Akiyama & Hayashi, 2002). In addition, the multiflorane-type esters (**148** − **149**) and triterpenoids **165** − **166**, **169**, and **175** were isolated from the seeds of *C. melo* var. *inodorus* and *C. melo* var. *reticulatus* (De Marino et al., 2009, Ibrahim et al., 2016). Other pentacyclic triterpenoids, including erythrodiol (**164**), alnusenol (**170**), alnusenone (**171**), and ursolic acid (**175**), were identified across species such as *C. dipsaceus*, *C. metuliferus*, and *C. trigonus* (Assefa et al., 2024, Busuioc et al., 2023, Ulubelen et al., 1976).

### 4.3 Flavonoids

Flavonoids are ubiquitously distributed throughout the plant kingdom and play key roles as phytoalexins and bioactive metabolites. In the genus *Cucumis*, they hold chemotaxonomic significance, particularly flavone glycosides, which were first reported in the leaves of several species (Krauze-Baranowska & Cisowski, 2001). Within the genus, *C. sativus* constitutes the most abundant source of such compounds, exhibiting considerable variation among 30 cultivars. The leaves and stems, in particular, have been shown to accumulate the highest concentrations, ranging from 793.70 to 971.75 mg/g dry weight (DW) (Olennikov & Kashchenko, 2023b). In this review, flavonoids (**176** − **265**) of genus *Cucumis*, detailed in Table S1 and Fig. 4, are categorized into flavones (**176** − **236**), flavonols (**237** − **245**), and homoisoflavonoid derivatives (**246** − **265**).

#### 4.3.1 Flavone derivatives

A total of 61 flavones (**176** − **236**) have been characterized in *Cucumis* species (Table S1, Fig. 4), primarily derived from apigenin (**176** − **217**), luteolin (**218** − **231**), and chrysoeriol (**232** − **236**) skeletons. These derivatives predominantly occur as *C*-, *O*-, and *C*,*O*-glycosides in the leaves, often in acylated forms with hydroxycinnamic acids such as ferulic, coumaric, caffeic, and sinapic acids. Fungal infection or stress induction enhanced the accumulation of various *C*,*O*-glycosylflavones, including known glycosides (**178** − **183**, **232**) and novel acylated derivatives (**184** − **186**, **233** − **234**). Further investigations revealed cucumerins A − B (**188** − **189**) and C − D (**194** − **195**) as new bioactive products in *C. sativus* cultivars (Mukherjee et al., 2013, Olennikov and Kashchenko, 2023c). Notably, isovitexin 2″-*β*-*O*-glucopyranoside (**190**) emerged as the principal flavone among many cultivars (Krauze-Baranowska & Cisowski, 2001). Additional novel acylated derivatives of isovitexin (**196** − **198**) and cucumosides A − K (**200** − **210**) were later isolated from the leaves of *C. sativus* (Olennikov and Kashchenko, 2023a, Olennikov and Kashchenko, 2024a, Olennikov and Kashchenko, 2024b). More recently, an advanced HPLC-PDA-ESI-tQ-MS/MS profiling technique further expanded this diversity, revealing the presence of acylated and non-acylated cucumerins, diverse *C*-, *O*-, and *C*,*O*-glycosides, and multiple apigenin derivatives in the leaves, stems, and flowers (Olennikov, 2023, Olennikov and Kashchenko, 2023b). Isovitexin (**178**) and isovitexin 2″-*β*-*O*-glucopyranoside (**190**) markedly increased under phosphate deficiency in *C. melo* shoots, reaching levels approximately 100 − 150 times higher than in the roots, although it has not yet been fully characterized. Phytochemical investigations of the leaves of *C. melo* var. *cantalupensis* and *C. melo* var. *reticulatus* revealed that both varieties share the occurrence of melosides A (**190**) and L (**221**), together with their caffeoyl esters (**199**, **228**). Other species, including *C. metuliferus* and *C. myriocarpus*, were also found to contain apigenin- and luteolin-based derivatives (e.g., **178**, **190**, **219**) (Hosoya et al., 2026, Krauze-Baranowska and Cisowski, 2001).

#### 4.3.2 Flavonol derivatives

Nine flavonols (**237** − **245**) have been reported in the literature of genus *Cucumis* (Table S1, Fig. 4). These include the phytoalexin rhamnetin (**237**), which accumulates in *C. sativus* seedlings in response to the fungal infection and silicon treatment (McNally, Wurms, Labbé, & Bélanger, 2003). Other flavonols comprise the aglycones kaempferol (**238**) and quercetin (**243**), together with their corresponding *O*-glycosides (**239** − **241**, **244** − **245**) (Anjani and Mathur, 2023, Ibrahim et al., 2019, Ibrahim and Mohamed, 2015, Mukherjee et al., 2013).

#### 4.3.3 Homoisoflavonoid derivatives

Homoisoflavonoids, a rare flavonoid subclass with one additional carbon atom, have been reported exclusively in *C. bisexualis*, the only cucurbitaceous plant known to produce these compounds. Twenty derivatives (**246** − **265**) of the sappanin-type were identified (Table S1, Fig. 4), classified into 3-benzylchroman-4-one (**246** − **249**) and Δ^3,9^-3-benzylchroman-4-one types (**250** − **265**), including four novel coumarin-homoisoflavonoids (**262** − **265**) (Ma et al., 2020b, Ma et al., 2021).

### 4.4 Coumarins and other phenolic compounds

The class of coumarins and other phenolics in genus *Cucumis* comprises a diverse array of secondary metabolites (Table S1, Fig. 5), including coumarins (**266** − **289**), chromones (**290** − **294**), quinones (**295** − **318**), lignans (**319** − **329**), aurones (**330** − **340**), biphenyls (**341** − **348**), and phenolic acids with phenolic glycosides (**349** − **359**). These metabolites are notable for their structural diversity, biological activities, and chemotaxonomic relevance within the genus.

#### 4.4.1 Coumarins

Coumarins (2*H*-1-benzopyran-2-one), structurally characterized as benzo-*α*-pyrone heterocycles, constitute a key class of plant phenolics within the genus. Phytochemical analysis of an ethanolic extract of *C. bisexualis* revealed 24 coumarin derivatives (**266** − **289**), including nine novel compounds (**269** − **277**) reported for the first time in nature (Table S1, Fig. 5) (Ma et al., 2021, Ma et al., 2018).

#### 4.4.2 Chromones

Chromones (benzo-*γ*-pyrones) are a rare subclass of phenolic compounds, differing from coumarins only in the position of the carbonyl group within the heterocyclic ring. Although rare in Cucurbitaceae, they have been exclusively reported from *C. melo* var. *reticulatus* seeds (Table S1, Fig. 5), where phytochemical studies yielded five new phenylethyl chromone derivatives (**290** − **294**) (Ibrahim, 2010, Ibrahim, 2014, Ibrahim and Mohamed, 2015).

#### 4.4.3 Quinones

Quinones represent a relatively minor phytochemical class in the genus, predominantly reported from *C. bisexualis*, with a total of 24 distinct anthraquinone and naphthoquinone derivatives identified to date (Table S1, Fig. 5). Bioassay-guided studies of ethanolic fruit extracts led to the isolation of 11 anthraquinones (**295** − **305**), including one novel anthraquinone-aurone adduct (**296**), and 13 naphthoquinones (**306** − **318**), with three newly identified compounds (**306** − **308**) (Ma and Wei, 2021a, Ma and Wei, 2021b, Ma et al., 2020c).

#### 4.4.4 Lignans

Lignans constitute a class of polyphenolic metabolites derived from the oxidative dimerization of coniferyl alcohol. In *Cucumis*, only a few representatives (Table S1, Fig. 5) have been reported, including the tetrahydrofurofuranoid- (**319**) and benzofuran- (**320**) lignan types from *C. sativus*, and three common lignans (**321** − **323**), which occur at higher levels in *C. melo* (0.71 μg/g DW) (Milder, Arts, van de Putte, Venema, & Hollman, 2005). In addition to classical lignans, *C. bisexualis* is the only species within the Cucurbitaceae reported to produce flavonolignans (non-conventional lignans). Six derivatives (**324** − **329**) have been identified from its fruits, among which stachyol C (**326**) is distinguished by a unique isoflavane-phenylpropanoid linkage (Ma, Liu, & Wei, 2024).

#### 4.4.5 Aurones

Aurones, a minor subclass of bioactive phenolics with a 2-benzylidene-3(2*H*)-benzofuranone scaffold, are biosynthesized in plants via oxidative cyclization of 2′-hydroxychalcones. In the Cucurbitaceae family, aurones have been documented solely in the fruits of *C. bisexualis*, from which 11 derivatives (**330** − **340**) have been characterized (Table S1, Fig. 5). These compounds are divided into three simple aurones (**330** − **332**), two geranyl-aurone derivatives (**333** − **334**), three novel coumarin-aurone heterodimers (**335** − **337**), and three auronolignans (**338** − **340**) (Ma and Wei, 2023, Ma et al., 2020a).

#### 4.4.6 Biphenyls

Biphenyls consist of two benzene rings linked at their 1,1′-positions. Within the Cucurbitaceae family, biphenyls have been reported exclusively from the fruits of *C*. *bisexualis*, from which eight derivatives (**341** − **348**) have been characterized (Table S1, Fig. 5). These include one new tetraphenylene (**341**), together with seven previously known biphenyl derivatives (**342** − **348**) (Ma, Liu, Sang, & Wei, 2025).

#### 4.4.7 Phenolic acids and phenolic glycosides

Phenolic acids and phenolic glycosides are key constituents of *Cucumis* phytochemistry with potential chemotaxonomic significance (Ibrahim, El-Hefnawy, & El-Hela, 2010). To date, ten phenolic acid derivatives (**349** − **358**) and a structurally unique phenolic glycoside (**359**) have been identified, thereby broadening the phenolic profile of the genus (Table S1, Fig. 5).

### 4.5 Volatile oils

Volatile oils are key contributors to the characteristic aroma of the genus *Cucumis*, with a diverse composition of esters, alcohols, aldehydes, and sulfur-containing compounds varying among species. In *C. sativus*, the aroma is derived mainly from fatty acid precursors, such as oleic, linoleic, linolenic, and palmitic acids, which give rise to aldehydes, alcohols, esters, alkanes, furfurans, and ketones, with the aldehydes and alcohols serving as the primary contributors to the cucumber’s distinct scent (Chen, Zhang, Hao, Chen, & Cheng, 2015). Unsaturated aldehydes, such as (*E*,*Z*)-2,6-nonadienal, (*E*)-2-nonenal, and (*E*)-2-hexenal, have been identified as the key determinants of the cucumber’s aroma, with (*E*,*Z*)-2,6-nonadienal recognized as the key marker compound, commonly referred to as “cucumber aldehyde” (Forss, Dunstone, Ramshaw, & Stark, 1962). Later analyses of essential oils from three *C. sativus* cultivars revealed 21 volatile compounds, with (*E*,*Z*)-2,6-nonadienal, (*Z*)-6-nonenol, and (*E*)-2-nonenal representing 88.5% of the total volatile content (Mukherjee, Nema, Maity, & Sarkar, 2013). Complementary studies have further expanded the volatile profile of the genus through the identification of several novel long-chain aldehydes in *C. sativus*, such as (*Z*)-8-pentadecenal, (*Z*)-7-hexadecenal, and (*Z*,*Z*,*Z*)-8,11,14-heptadecatrienal. In addition, volatile organic compounds from the peel and flesh tissues, including (*E*,*Z*)-2,6-nonadienal, (*Z*)-6-nonenol, (*E*)-2-nonenal, nonanal, 1-nonanol, and hexanal, were shown to constitute the majority of the essential oil content (80%−85%) (Kemp, 1977).

The aroma profile of *C. melo* is more complex, combining fruity esters with a characteristic musky undertone largely attributable to sulfur-containing compounds. In *C. melo* var. *reticulatus*, sulfur volatiles such as methyl-(methylthio) acetate, ethyl-(methylthio) acetate, 3-(methylthio) propanitrile, and 3-(methylthio) propanol were identified as key contributors (Jordán, Shaw, & Goodner, 2001). Other reports identified (*Z*)-6-nonen-1-ol with a cucumber-like scent, together with nine-carbon alcohols and aldehydes, such as (*Z*)-6-nonenal and (*Z*,*Z*)-3,6-nonadien-1-ol. Methyl and ethyl esters derived from fatty acids further shaped the aroma profile, with the former being the dominant (Kemp, Stoltz, & Knavel, 1972). In *C. melo* var. *inodorus*, unique compounds such as (*Z*)-3-nonenyl acetate, (*Z*,*Z*)-3,6-nonadienyl acetate, 3-methyl-2-butenyl acetate, and ethyl 2-(methylthio) acetate were characterized, alongside 25 additional aroma constituents, including five newly reported volatiles (Buttery et al., 1982). Similarly, in *C. melo* var. *cantalupensis*, sulfur-containing volatiles including 2-(methylthio) ethanol were prominent, while 55 volatiles, mostly esters, were detected in cantaloupe and honeydew melons, including dimethyl disulfide, the first sulfur-containing volatile reported in the genus *Cucumis* (Yabumoto & Jennings, 1977). A distinct profile was observed in *C. melo* var. *dudaim*, characterized by hexanol (11.52%), chavicol (11.33%), (*Z*)-3-hexenol (6.84%), benzyl alcohol (3.54%), and eugenol (3.48%), with chavicol being the dominant contributor to its unique scent (Shu, Chung, & Lawrence, 1995). Notably, 3-methylthiopropionic acid ethyl ester (MTPE) is a common volatile across *C. melo* varieties, including *C. melo* var. *conomon*, *C. melo* var. *cantalupensis*, *C. melo* var. *reticulatus*, and *C. melo* var. *dudaim* (Kamimura et al., 2023).

### 4.6 Other compounds

Besides the major classes of secondary metabolites described above, several additional groups of compounds have been reported in *Cucumis* species (Table S1, Fig. 6). These include polysaccharides (**360** − **361**), strigolactones (**362** − **370**), C_13_-norisoprenoid megastigmanes (**371** − **376**), gibberellins (**377** − **386**), nitrogenous compounds (**387** − **402**), and miscellaneous metabolites (**403** − **428**).

#### 4.6.1 Polysaccharides

Two novel pectic polysaccharides, CMPP-1 (**360**) and CMPP-2 (**361**), were isolated from the fruit peels of *C. metuliferus* (Table S1, Fig. 6). Both are hetero-galacturonans with molecular weights of 7.35 and 6.90 kDa, predominantly composed of glucuronic acid and exhibiting highly branched pectic structures as confirmed by FT-IR and NMR analyses (Zhu et al., 2021).

#### 4.6.2 Strigolactones

Strigolactones represent a distinct group of carotenoid-derived metabolites identified only in *C. sativus*. Nine orobanchol-type analogues (**362** − **370**) have been identified, including the novel metabolites 7*α*-hydroxyorobanchol (**363**) and 7*β*-hydroxyorobanchyl acetate (**364**) isolated from its root exudates (Table S1, Fig. 6) (Khetkam et al., 2014).

#### 4.6.3 C_13_-Norisoprenoid megastigmanes

Six C_13_-norisoprenoid megastigmanes (**371** − **376**) belonging to a class of monocyclic terpenoids biosynthetically derived from carotenoids and characterized by diverse substitution patterns, have been isolated from *Cucumis* species (Table S1, Fig. 6) (El-Sayed, Rasheed, Mahrous, & Abdel-Sattar, 2025). These include the *α*-ionol (**371** − **373**), *α*-ionone (**374**), and allenic megastigmane derivative (**376**), including the novel cucumegastigmanes I − II (**371** − **372**) isolated from both *C. sativus* and *C. melo* leaves (Hosoya et al., 2026, Mukherjee et al., 2013).

#### 4.6.4 Gibberellins

Gibberellins are tetracyclic diterpenoidal plant hormones with an *ent*-gibberellane skeleton, identified in both the seeds and aerial parts of *Cucumis* species. A total of ten gibberellins (**377** − **386**) have been reported (Table S1, Fig. 6), with gibberellin A_1_ (**377**) being the most abundant in *C. sativus* seeds. Additionally, three novel conjugated gibberellins (**381** − **383**) were characterized, and the 13-deoxygibberellins (**379**, **385**) were found to predominate over the 13-hydroxygibberellins (**377**, **384**, **386**) in the aerial tissues (Hemphill et al., 1972, Hemphill et al., 1973, Smith et al., 1991).

#### 4.6.5 Nitrogenous compounds

Sixteen nitrogenous metabolites (**387** − **402**) have been reported from *Cucumis* plants, mainly *C. sativus* (Table S1, Fig. 6). These include the indole derivatives (**387** − **391**) from various organs (Mukherjee et al., 2013, Zhou et al., 2012), flavins (**392** − **393**) under iron deficiency (Satoh et al., 2016), and non-protein amino acids (**394** − **396**) serving as chemotaxonomic markers in *C. sativus*, *C. melo*, and *C. ficifolius* (Dunnill & Fowden, 1965). Additional metabolites comprise the iminosugar idoBR1 (**397**) and sphingolipids (**399** − **402**) isolated from *C. sativus* (Tang et al., 2010), whereas a novel *N*-trisaccharide (**398**) has been reported from *C. prophetarum* (Kavishankar and Lakshmidevi, 2014, Nash et al., 2020).

#### 4.6.6 Miscellaneous metabolites

In addition to the aforementioned compounds, miscellaneous metabolites identified from *Cucumis* species comprise organic acids (**403** − **404**), the carotenoid lutein (**405**), simple glycosides (**406** − **407**), simple phenols (**408** − **410**), a triglyceride (**411**), a series of other metabolites (**412** − **418**), and a range of diverse lipid molecules (**419** − **428**) (Table S1, Fig. 6).

## 5 Pharmacological activities of *Cucumis* species

**Fig. 7:**
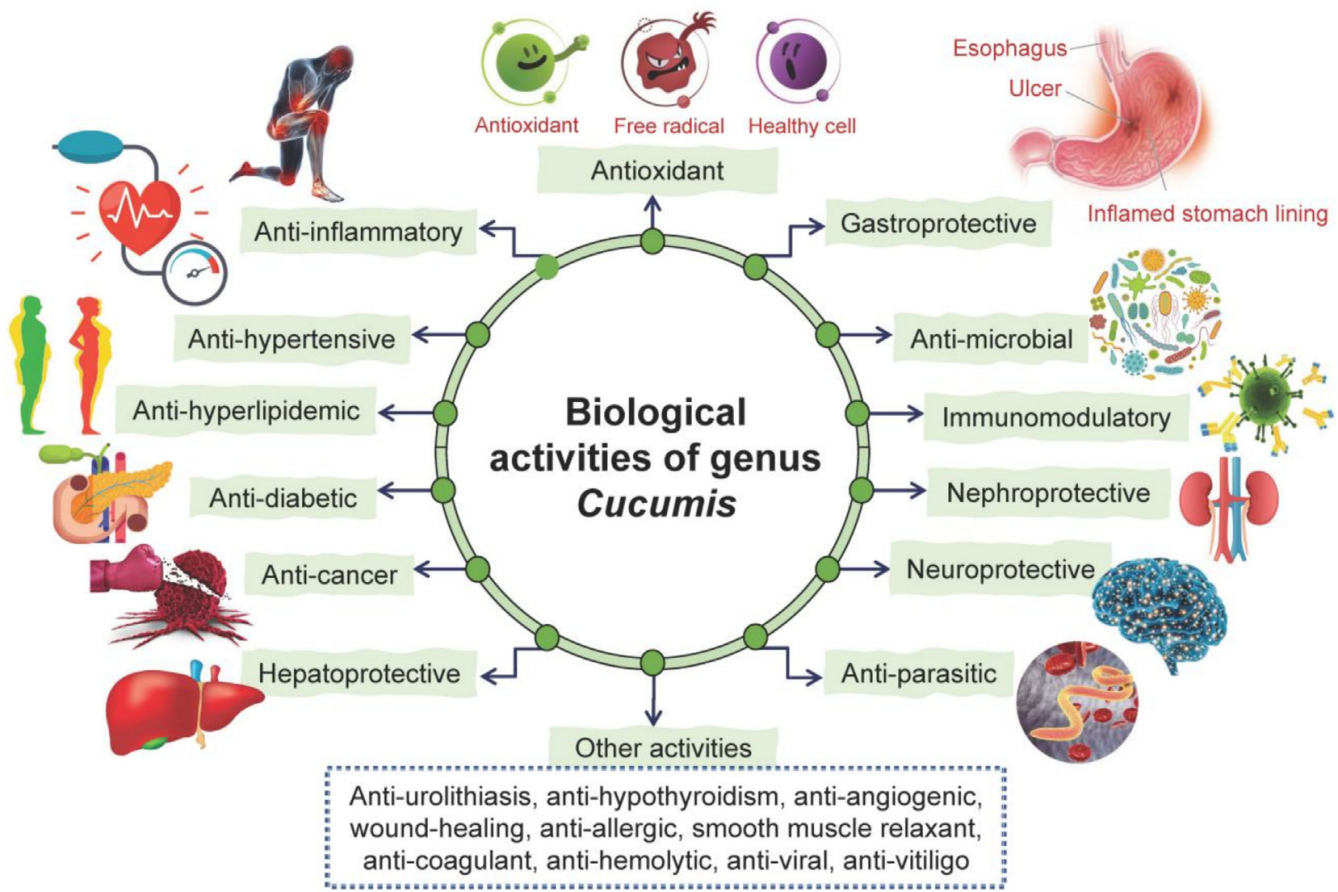
Reported pharmacological activities of genus *Cucumis*.

Integrating the ethnomedicinal knowledge with phytochemical and pharmacological research is essential to evaluate the therapeutic potential of *Cucumis* plants and support novel medical applications (Wahid et al., 2022, Wahid et al., 2024). Of the 60 recognized species, only 12 have been investigated pharmacologically, with *C. sativus* and *C. melo* being the most studied. Other species, such as *C. metuliferus*, *C. dipsaceus*, *C. callosus*, *C. prophetarum*, and *C. ficifolius*, remain underexplored. The pharmacological activities of the remaining species, particularly less accessible ones, are still largely unknown, highlighting the need for further research. The identified phytochemicals (Table S1) likely contribute synergistically to the pharmacological effects summarized in Fig. 7.

### 5.1 Antioxidant activities

Oxidative stress arises from an imbalance between reactive oxygen species (ROS) generation and antioxidant defenses, leading to cellular, protein, and DNA damage implicated in cancer, inflammation, and aging (Paul et al., 2024). *Cucumis* species exhibit strong antioxidant potential, mainly due to flavonoids and phenolics, supporting their ethnomedicinal use (Section 3). In *C. sativus*, the ethanolic peel extract demonstrated the highest antioxidant activity, with ferric reducing antioxidant power (FRAP) values ranging from 0.03 to 0.12 mmol Fe^2+^/g and up to 71% FRAP and 54% 2,2-diphenyl-1-picrylhydrazyl (DPPH) radical scavenging at 300 µg/mL, which is consistent with its elevated phenolic [23.08 mg gallic acid equivalents (GE)/g] and flavonoid [14.02 mg quercetin equivalents (QE)/g] contents. In contrast, the aqueous fruit flesh extract exhibited the lowest antioxidant capacity; however, at 500 µg/mL, it still demonstrated moderate scavenging effects in the DPPH (56%) and nitric oxide (NO) (53%) assays (Anjani, Srivastava, & Mathur, 2023). The methanolic seed extract showed over 76% scavenging activity, while its 9*β*-methyl-19-norlanosta-5-ene-type glycoside showed 73% (Gill & Bali, 2012).

*C. melo* varieties also demonstrated high superoxide dismutase (SOD) activity where the aqueous fruit pulp extracts of *C. melo* var. *cantalupensis* suppressed ROS generation, while the methanolic extracts of *C. melo* var. *reticulatus* leaves and stems showed IC_50_ values of 1.52 − 2.16 mg/mL in DPPH assays (Gómez-García et al., 2020, Vouldoukis et al., 2004). The flavonoids (**181**, **184**, **190**, **199**, **217**, **221**, **228** − **230**, **243**) and chromone derivatives (**290** − **294**) isolated from *C. melo* var. *reticulatus* displayed potent radical scavenging activity, with acylated flavones (**199**, **217**) being the most active (Hosoya et al., 2026, Ibrahim, 2014, Ibrahim and Mohamed, 2015). The ethanolic pulp extracts of *C. melo* var. *inodorus* exhibited strong antioxidant activity in multiple *in vitro* assays, with the 2,2′-azino-bis(3-ethylbenzothiazoline-6-sulfonic acid) (ABTS) assay showing the highest potency (IC_50_ = 0.64 mg/mL). *In vivo*, the aqueous fruit pulp extracts inhibited malondialdehyde (MDA) release, reduced lipid peroxidation, and enhanced antioxidant enzymes such as SOD and catalase (CAT) (Adebayo-Gege et al., 2023, Barghout et al., 2024). *C. melo* var. *utilissimus* seed extracts achieved inhibition of 73% (DPPH, 300 µg/mL) and 57% (H_2_O_2_, 200 µg/mL), while *C. melo* var. *momordica* seed extracts showed IC_50_ values of 60.70 and 62.15 µg/mL in DPPH and ABTS, respectively (Gill et al., 2010, Gómez-García et al., 2020, Silva et al., 2020, Yadav et al., 2022).

Other species displayed similar activities where the methanolic fruit extract of *C. prophetarum* and its cucurbitacin B (**91**) showed strong antioxidant effects against carrageenan (CGN)-induced prostatic damage, while the aqueous extracts demonstrated DPPH, superoxide, and metal chelation activities with IC_50_ values of 78 − 194 µg/mL. The leaves, rich in flavonoids and phenolics, exhibited considerable FRAP and H_2_O_2_ scavenging activity, while the root extracts, rich in sterols (**23** and **36**) and cucurbitacins (**132** − **133**), revealed 70% DPPH scavenging and 53% anti-lipid peroxidation activities (Aljohani, 2022, Galma et al., 2021, Gawli and Lakshmidevi, 2015). Similarly, the extracts of *C. africanus* fruits, leaves, and roots, particularly the acetone fruit extract, displayed potent activities in DPPH, ABTS, and phosphomolybdenum assays, correlated with their polyphenolic contents (Abifarin, Afolayan, & Otunola, 2019). The seed extracts of *C. callosus* also showed strong antioxidant potential (DPPH, IC_50_ = 24.27 µg/mL; H_2_O_2_, IC_50_ = 153.35 µg/mL) supported by high phenolic (110.4 mg GE/g) and flavonoid (92.4 mg QE/g) contents (Panda, Jena, Kalyani, & Panigrahy, 2018). In *C. ficifolius*, the methanolic root extract and its chloroform fraction exhibited strong DPPH scavenging (IC_50_ = 9.10 − 19.40 µg/mL) (Araya, Adamu, Periasamy, Sintayehu, & Gebrelibanos Hiben, 2019). The hydroethanolic fruit extract of *C. metuliferus* and its ursolic acid (**175**) displayed stronger antioxidant effects than ascorbic acid and trolox, with IC_50_ = 32.74 µg/mL in DPPH and 11.37 µg/mL in ABTS assays (Busuioc, Costea, Botezatu, Furdui, & Dinica, 2023). The ethanolic extracts of *C. anguria* leaves showed strong DPPH scavenging linked to flavonoids and saponins, while the hairy root extract and the non-transformed root extract also demonstrated radical scavenging of 61% and 57%, respectively (Yoon, Chung, & Thiruvengadam, 2015). *In vivo*, the flavonoid-rich extracts of *C. dipsaceus* fruits exhibited strong radical scavenging and anti-lipid peroxidation activities by restoring SOD, CAT, and glutathione (GSH) while reducing thiobarbituric acid reactive substances (TBARS)/MDA levels (Lata and Mittal, 2017b, Lata and Mittal, 2017). It’s worth mentioning that their phenolic, flavonoid, and tannin contents remained stable under gastric pH, and thermally processed extracts showed higher ROS inhibition and FRAP activity than rutin (Paul et al., 2024). Likewise, the ethanolic fruit extracts of *C. pubescens*, rich in phenolics, flavonoids, and tannins, exhibited dose-dependent antioxidant activity in DPPH, H_2_O_2_, and NO assays, with DPPH radical scavenging reaching 95% at 125 µg/mL (Sundari & Kavitha, 2024).

### 5.2 Anti-inflammatory and analgesic activities

Although inflammation is a natural defense mechanism, it contributes to chronic diseases such as asthma, diabetes, arthritis, neurodegenerative and cardiovascular disorders, autoimmune conditions, and cancer. Plant-derived extracts may provide effective anti-inflammatory benefits with fewer side effects than conventional drugs (Ezzat, Raslan, Salama, Menze, & El Hawary, 2019). For example, the aqueous fruit extract of *C. sativus* showed significant analgesic activity in mice at 500 mg/kg in both tail immersion and acetic acid-induced writhing tests. The fruit homogenate (2.0 − 4.0 mL/kg) also produced notable anti-inflammatory effects with reduced paw edema (Idemudia and Enogieru, 2024, Khan et al., 2022). The iminosugar idoBR1 (**397**) reduced LPS-induced TNF-*α* in THP-1 cells *in vitro* and blood *ex vivo* by over 50% at 10 and 0.1 µmol/L, respectively, and inhibited bacterial and human sialidase (IC_50_ = 0.5 mmol/L, 60% inhibition) compared with oseltamivir (IC_50_ = 0.25 mmol/L) (Nash et al., 2020). Additionally, an aqueous fraction of ethanolic aerial extracts (10 µg/mL) suppressed angiotensin II (Ang II)-induced inflammation in HMEC-1 cells, modulating TNF-*α*, IL-1, IL-6, IL-17, COX-II, and NF-*κ*B, likely through amino acids (e.g., glycine, *L*-arginine) and polysaccharides (Trejo-Moreno et al., 2018).

Due to the high SOD activity (100 IU-NBT/mg), the fruit extracts of *C. melo* varieties exhibit strong anti-inflammatory properties by inhibiting TNF-*α* and promoting IL-10 production in both macrophages and C57BL/6 mice (Vouldoukis et al., 2004). At 50 mg/kg, the ethanolic extracts of *C. melo* var. *cantalupensis* or *C. melo* var. *reticulatus* reduced CGN-induced edema by 37.90%−69.41%, comparable to indomethacin (68.49% at 10 mg/kg). They also inhibited key inflammatory mediators (including TNF-*α*, IL-1*β*, prostaglandin E_2_ (PGE_2_), and IL-6), with the pulp extracts being the most effective (Ezzat, Raslan, Salama, Menze, & El Hawary, 2019). The ethanolic pulp extract of *C. melo* var. *inodorus* (600 mg/kg) also showed analgesic and anti-inflammatory effects in rodents (Barghout et al., 2024). The methanolic extracts of *C. melo* var. *flexuosus* organs downregulated COX-II, NF-*κ*B, and TNF-*α* in RAW 264.7 macrophages, with the leaves and seeds extracts being the most active due to their rich bioactive contents (El-Sayed et al., 2025). Likewise, *C. melo* var. *utilissimus* seed extract reduced CGN-induced edema (39% at 200 mg/kg; 54% at 300 mg/kg) and produced central analgesic effects in mice in the tail immersion and tail flick assays (Gill et al., 2010, Silva et al., 2020).

Other *Cucumis* species also exhibit notable analgesic and anti-inflammatory activities. The daily administration of *C. prophetarum* fruit methanolic extract and its different fractions protected against the pathological tissue alterations and attenuated the inflammatory mediators (TNF-*α*, IL-1*β*, iNOS, COX-II) in CGN-induced prostatitis in rats. Its chloroform fraction (100 mg/kg) and cucurbitacin B (**91**; 5.0 mg/kg) showed superior efficacy over other extracts (Aljohani, 2022). Similarly, *C. ficifolius* root methanolic extract (800 mg/kg) and its *n*-butanol and aqueous fractions (200 mg/kg) produced strong peripheral analgesic effects (62%−72%) in the writhing test and central anti-nociceptive activity (82%−89%) in the hot plate test, alongside significant anti-inflammatory effects (56%−71%) in CGN-induced models, supporting its ethnomedicinal use against pain and inflammation (Demsie, Yimer, Berhe, Altaye, & Berhe, 2019). The ethanolic extract of *C. metuliferus* fruit demonstrated potent *in vitro* anti-proteinase (IC_50_ = 16.34 µg/mL) and anti-lipoxygenase (IC_50_ = 32.90 µg/mL) activities, mainly due to ursolic acid (**175**; IC_50_ = 12.53 and 18.61 µg/mL, respectively) (Busuioc, Costea, Botezatu, Furdui, & Dinica, 2023). In mice, *C. anguria* fruit extract (100 mg/kg) also caused significant anti-nociceptive and analgesic effects and reduced acetic acid-induced writhing by 85%, exceeding aspirin’s 68% reduction at 200 mg/kg (Salama & Solano, 2001). Likewise, *C. trigonus* fruit extract showed remarkable analgesic potency in the tail clip and writhing tests with ED_50_ = 2.5 mg/kg, compared to sodium salicylate (ED_50_ = 240 mg/kg), and exerted anti-inflammatory effects in CGN-induced edema and cotton pellet-induced granuloma with ED_50_ values (17 − 20 mg/kg) superior to phenylbutazone (Naik, Agshikar, & Abraham, 1980).

### 5.3 Cardiovascular protective activities

Cardiovascular diseases remain the leading cause of global mortality, with hypertension, thrombosis, oxidative stress, and impaired cardiac and renal function as key risk factors. Herbal medicines are gaining increasing attention for their ability to modulate vascular tone, prevent clot formation, improve myocardial function, and regulate fluid-electrolyte balance (Wahid et al., 2022, Wahid et al., 2024). Consistently, *Cucumis* species have demonstrated a wide spectrum of cardiovascular protective activities, including antihypertensive, cardioprotective, diuretic, as well as anticoagulant and anti-hemolytic effects.

A concentrated *C. sativus* fruit juice (36 mg/kg) significantly reduced Ang II-mediated vasoconstriction in rats, while its co-administration with losartan (2.25 mg/kg) at a lower dose (18 mg/kg) achieved superior blood pressure reduction compared to either treatment alone. These effects may be attributed to flavonoids, saponins, terpenoids, and minerals such as Ca, K, Mg, and Zn (Hendrayana, Yoana, Adnyana, & Sukandar, 2023). Similarly, the aqueous fraction of *C. sativus* aerial parts, rich in glycine, *L*-arginine, and polysaccharides, exerted vasodilatory activity and protected against Ang II-induced vascular damage (Trejo-Moreno et al., 2018). Kaempferol (**238**) isolated from the fruits improved lipid profile and antioxidant status, suggesting preventive potential against cardiovascular disorders (Ibitoye, Uwazie, & Ajiboye, 2018). Moreover, the crude seed extract (5.0 − 10 mg/mL) induced endothelium-dependent vasorelaxation via calcium channel antagonism and NO signaling, reduced contractile force, and increased heart rate in rats. Intravenous administration of the extract also lowered arterial pressure and heart rate in normotensive rats at 0.3 − 1.0 mg/kg, alleviated *L*-NAME-induced acute hypertension, and protected against isoproterenol (ISO)-induced chronic cardiac injury through antioxidant and anti-inflammatory mechanisms. These actions could explain the traditional use of the seeds as cardiovascular protective agents. *C. melo* seed extract exhibited similar vasorelaxant, negative inotropic, and positive chronotropic activities *in vitro*, and antihypertensive and cardioprotective effects *in vivo*. These activities could be attributed mainly to vitexin (**187**), orientin (**218**), and gallic acid (**349**) (Wahid et al., 2022, Wahid et al., 2024). In high-fat diet (HFD)-induced obesity, the methanolic seed extract of *C. melo* var. *inodorus* (100 mg/kg) reduced plasma atherogenic index (AIP), Castelli index, adiposity index, and MDA levels via antioxidant and NO-dependent pathways (Adebayo-Gege et al., 2022). The extracts enriched with cucurbitacins B (**91**), E (**95**), and D (**108**) significantly attenuated Ang II-induced hypertension in mice at 1.0 mg/kg without toxicity, an effect explained by enhanced acetylcholine-mediated vasodilation and inhibition of phenylephrine-mediated vasoconstriction. Pedicels (or *Pedicellus Melo*), rich in cucurbitacin B (**91**), further validates its ethnomedicinal use in the traditional Chinese medicine for lowering blood pressure (Yuan et al., 2019).

*Cucumis* species also exhibit notable diuretic activity, promoting urine and electrolyte excretion. For example, the ether extract of *C. melo* seeds (300 − 400 mg/kg, i.v.) markedly increased the urine volume (151 mL *vs* 96 mL in control) and chloride excretion (114 mEq/L *vs* 95 mEq/L in control) in dogs, comparable to mercurial and xanthine diuretics (Wright, Van-Buren, Kroner, & Koning, 2007). Moreover, the aqueous and methanolic extracts of *C. dipsaceus* leaves (200 − 400 mg/kg) induced dose-dependent diuresis and elevated the Na^+^/K^+^ ratio in rats, confirming its safe ethnomedicinal use (Asefa & Nedi, 2024). Likewise, the alcoholic fruit extract of *C. trigonus* (25 − 50 mg/kg, p.o.) enhanced Na^+^ and Cl^−^ excretion without affecting K^+^, showing stronger diuretic and antihypertensive effects than hydrochlorothiazide (25 mg/kg), likely due to its glycoside contents, thereby validating its ethnomedicinal application (Naik et al., 1981, Wright et al., 2007).

Beyond their cardiovascular protective roles, *Cucumis* species also display anticoagulant and anti-hemolytic activities. For example, the different *C. melo* rind extracts and fractions (30 µL) exhibited potent heparin-like anticoagulant effects *in vitro*, significantly prolonging cephalin-kaolin clotting time and prothrombin time, with the *n*-butanol fractions showing the strongest influence on the extrinsic pathway (Derradji & Aoun, 2022). Moreover, the ethanolic pulp extract of *C. melo* var. *inodorus* (1.0 mg/mL) provided 75% inhibition of protein denaturation and conferred 70% protection against AAPH-induced red blood cell lysis at 4.0 mg/mL, confirming its anti-hemolytic and cytoprotective potential (Barghout et al., 2024).

### 5.4 Metabolic-related disorders (anti-obesity and anti-diabetic activities)

Hyperlipidemia and diabetes mellitus are major metabolic disorders strongly associated with obesity, atherosclerosis, and cardiovascular complications. Regulation of body weight, body mass index (BMI), lipid profile parameters, including total cholesterol (TC), triglycerides (TG), low-density lipoprotein (LDL), very low-density lipoprotein (VLDL), and high-density lipoprotein (HDL), and glucose homeostasis is therefore critical in preventing these pathologies. Nutrient-rich *Cucumis* fruits and vegetables, such as cucumber and melon, aid in obesity and diabetes management owing to their high water, fiber, and antioxidant contents, which collectively help regulate lipid metabolism, reduce oxidative stress, and improve insulin sensitivity (Kavishankar et al., 2014, Marisol et al., 2019).

The methanolic extracts of *C. sativus* fruits yielded triterpenoidal saponins that inhibited the pancreatic lipase (PL) more effectively than orlistat, while kaempferol (**238**) reversed alloxan-induced lipid profile disturbances (Ibitoye et al., 2018, Subandi et al., 2018). Cucumerins A − D (**188** − **189**, **194** − **195**) and other flavonoids isolated from the leaves and flowers inhibited PL, with IC_50_ values of 41.70 − 77.60 mmol/L, close to orlistat. Notably, cucumerins B (**189**) and D (**195**) showed greater potency, with IC_50_ values of 25.63/12.53 µmol/L against porcine PL (PPL), and 20.89/10.35 µmol/L against human PL (HPL), respectively (Olennikov and Kashchenko, 2023a, Olennikov and Kashchenko, 2023c). Similarly, the luteolin derivatives, such as isoorientin (**219**) and its glucoside (**221**), demonstrated potent activity with IC_50_ values of 22.63/12.68 µmol/L (PPL) and 15.32/10.06 µmol/L (HPL), comparable to orlistat (IC_50_ = 10.18 µmol/L, PPL; 15.83 µmol/L, HPL) (Olennikov, 2023). In addition, isovitexin derivatives (e.g., **181**, **184** − **186**, **196** − **198**) inhibited the hydrolysis of long-, medium-, and short-chain fatty acids, with IC_50_ values of 1.62 − 14.83 µg/mL (Olennikov & Kashchenko, 2023a). *In vivo*, the flavonoid-rich extracts (100 mg/kg/d) obtained from the stems and leaves improved serum lipid profiles in hyperlipidemic hamsters, with isovitexin 2″-*O*-glucoside-6″-*O*-*p*-coumarate (**197**) normalizing the lipid metabolism over six months at 20 − 50 mg/kg/d. Unlike simvastatin (a reference antihyperlipidemic drug), these extracts demonstrated strong antioxidant effects, implying dual benefits against dyslipidemia and oxidative stress (Olennikov & Kashchenko, 2023b). Supporting these findings, the aqueous subfractions of *C. sativus* aerial parts (20 − 40 µg/mL) significantly improved adipocyte function by effectively controlling dexamethasone- and IL-1*β*-induced dysfunction in 3 T3-L1 adipocytes *in vitro*, enhancing glucose utilization by 57%−87% and glycerol secretion by 10.6%−18.9%, with glycine, asparagine, and *L*-arginine identified as the key bioactive components (Marisol et al., 2019).

Complementary evidence highlights the anti-diabetic potential of *C. sativus*. The lyophilized fruit juice (100 − 400 mg/kg) and kaempferol (**238**) inhibited *α*-amylase and *α*-glucosidase (IC_50_ = 652.43 and 51.24 µg/mL, respectively), improving glycemic control *in vitro* and in the alloxan-induced diabetic rats (Ibitoye, Uwazie, & Ajiboye, 2018). The ethanolic fruit extracts (300 mg/kg) significantly reduced fasting blood glucose and enhanced cardiac antioxidant enzymes in the STZ-induced diabetic rats, while its iminosugar idoBR1 (**397**) was identified as a selective human glucosidase inhibitor, further supporting the fruit’s anti-diabetic potential (Nash et al., 2020, Roman-Ramos et al., 1995). Furthermore, fruit extracts modulated oxidative stress and inflammatory pathways associated with metabolic syndrome, underscoring their therapeutic promise in managing obesity-linked diabetes and related complications (Marisol et al., 2019).

Similarly, *C. melo* fruits and leaves demonstrated strong anti-hyperlipidemic and anti-obesity activities. The methanolic and aqueous fruit peel extracts (500 mg/kg/d) improved the lipid parameters, body weight, creatinine kinase-MB, and AIP in cholesterol-fed rats, with effects equipotent to atorvastatin. At a lower dose (100 mg/kg/d), the peel extract significantly reduced tissue lipid peroxidation (LPO) and modestly lowered TC and LDL levels (Parmar & Kar, 2009). The ethanolic extracts of *C. melo* var. *agrestis* fruits and fractions (50 mg/kg/d) ameliorated HFD-induced dyslipidemia in hamsters, with the *n*-hexane fraction suppressing adipogenesis and enhancing lipid metabolism, compared to fenofibrate (100 mg/kg). In contrast, the methanolic extracts of its leaves reduced hyperlipidemia and hyperglycemia in streptozotocin (STZ)- and nicotinamide (NIC)-induced diabetic rats, likely due to cucurbitacins (**96** − **99**), flavonols (**243** − **244**), and gallic acid (**349**) (Gopalasatheeskumar et al., 2020, Ul Haq et al., 2019). Likewise, the ethanolic extracts of *C. melo* var. *momordica* fruits (400 mg/kg) and its toluene fraction (50 mg/kg) showed significant anti-hyperlipidemic activity in diabetes-associated dyslipidemia in STZ-diabetic models. Both the extract and fraction reduced the blood glucose from 15.51 mmol/L to 8.89 mmol/L and 6.77 mmol/L respectively after 28 d (Srivastava et al., 2020, Yadav et al., 2022).

Other *Cucumis* species also exhibit notable anti-obesity and anti-diabetic effects. The aqueous fruit extract of *C. prophetarum* and its fractions exhibited notable anti-diabetic activity by inhibiting *α*-amylase and *α*-glucosidase (IC_50_ = 20.6 µg/mL and 59.9 µg/mL), thereby supporting glycemic control. Complementarily, its *N*-trisaccharide (**398**) reduced the blood glucose levels by 47%−69%, restored antioxidant status, improved lipid profiles, and significantly enhanced plasma insulin without inducing hypoglycemia in STZ- and NIC-diabetic models at 25−50 mg/kg (Gawli and Lakshmidevi, 2015, Kavishankar and Lakshmidevi, 2014, Kavishankar et al., 2014). In STZ-induced diabetic rats, *C. trigonus* aqueous fruit extract (500 mg/kg/d) markedly reduced serum glucose (by 56%, compared to glibenclamide’s 28% inhibition), glycated hemoglobin (HbA_1C_, by 41%), TC, TG, LDL, and VLDL, while concomitantly increasing HDL, serum insulin, and liver glycogen after 21 d of treatment (Salahuddin & Jalalpure, 2010). In STZ-diabetic rats, the methanolic extract of *C. callosus* fruit (400 mg/kg), along with cucurbitacin B (**91**) (80 µg/kg), similarly regulated lipid parameters and serum glucose (Deepika et al., 2023). *C. metuliferus* ethanolic fruit extract and its ursolic acid (**175**)-rich fraction effectively inhibited *α*-amylase and *α*-glucosidase, comparable to acarbose, and demonstrated dose-dependent hypoglycemic activity (500−1 500 mg/kg) in the alloxan-induced hyperglycemia (Busuioc et al., 2023, Šeregelj et al., 2022). Similarly, *C. dipsaceus* methanolic fruit extracts inhibited *α*-amylase (69%) and *α*-glucosidase (88%), slightly exceeding acarbose (Paul et al., 2024). Collectively, these findings highlight the significant anti-hyperlipidemic and anti-obesity potential of *Cucumis* species, supporting their ethnomedicinal uses.

### 5.5 Anti-cancer and immunomodulatory activities

*Cucumis* species are rich in cucurbitacins, flavonoids, and phenolics that exert dual immunomodulatory and anti-cancer activities. Their immunomodulatory effects are mediated through macrophage activation and stimulation of cytokines (NO, IL-6, TNF-*α*). For instance, the ethanolic leaf extract of *C. sativus* (10 µg/mL) inhibited encephalitogenic T cells, reduced IFN-*γ* and IL-17 *in vivo*, and decreased NO and TNF-*α* in LPS-activated macrophages, with cucurbitacins B (**91**), E (**95**), flavonoids (**176** − **177**, **187**), and phenolics (**350** − **351**) as the main contributors (Jevtić et al., 2017). Likewise, pectin polysaccharides CMPP-1 (**360**) and CMPP-2 (**361**) from *C. metuliferus* fruit peels enhanced NO and cytokine production in RAW 264.7 cells, with CMPP-1 (**360**) showing activity at 0.78 µg/mL and CMPP-2 (**361**) at 6.25 µg/mL, confirming their immune-boosting role (Zhu et al., 2021).

These immunostimulatory mechanisms are strongly linked to the anti-cancer potential of *Cucumis* species, as multiple extracts and cucurbitacins have shown efficacy against diverse cancer cells, including liver, lung, ovary, colon, breast, melanoma, and leukemia (Miró, 1995, Raja Soh et al., 2024). For example, cucurbitacin C (**77**) and its derivatives (**79** − **80**) isolated from *C. sativus* leaves suppressed the proliferation of prostate (LNCaP, PC-3, DU145), lung (A549), colon (HCT116), bladder (T24), and liver (HepG2) cancer cells by 40%−60%. Cucurbitacin C (**77**) has also demonstrated anti-cancer potential in both *in vitro* and *in vivo* studies, primarily through modulation of the PI3K-Akt signaling pathway leading to cell growth arrest and apoptosis. Similarly, cucurbitacins C_1_ − C_7_ (**84** − **90**) exhibited strong cytotoxicity against DU145, A549, HCT116, and HepG2 cells, with C_6_ (**89**; IC_50_ = 10.06 µmol/L) and C_7_ (**90**; IC_50_ = 4.16 µmol/L) showing activity close to taxol (Chen et al., 2005, Qing et al., 2022). The seed oil (100 µg/mL) also inhibited DU145 cell proliferation, induced apoptosis, and suppressed migration and invasion. In the benzo(*α*)pyrene-induced prostate cancer rat model, the oil (85 − 170 mg/kg) reduced cancer incidence from 75% to 12.5%, comparable to casodex, while enhancing antioxidant (SOD, CAT, GSH) and anti-inflammatory (IL-10) markers, and lowering MDA, TNF-*α*, IL-1, and IL-6 (Bakam et al., 2023).

*C. melo* var. *reticulatus* peel and seed extracts showed dose-dependent anti-proliferative effects against cervical (HeLa, SiHa), kidney (786-O), and colon (HT-29) cancer cells, with the seed extracts achieving 65%−87% inhibition (IC_50_ = 0.3 mg/mL) (Raja Soh, Hapidin, & Kasiram, 2024). Consistently, the methanolic seed extract also displayed strong cytotoxicity in brine shrimp bioassay (80% mortality at 200 µg/mL), likely due to cucumols A − B (**165** − **166**) and chromone derivative (**293**). Cucumol A (**165**) exhibited cytotoxicity against lymphoma (L5178Y, ED_50_ = 1.30 µg/mL) and cervical (Hela, ED_50_ = 5.40 µg/mL) cancer cells, whereas cucumol B (**166**) was active against ovarian (SKOV-3, IC_50_ = 2.05 µmol/L) and breast (MCF-7, IC_50_ = 0.41 µmol/L) adenocarcinomas. The chromone derivative (**293**) showed cytotoxic activity only against L5178Y cells (ED_50_ = 5.00 µmol/L) (Gómez-García et al., 2020, Ibrahim et al., 2016, Ibrahim, 2010, Ibrahim, 2014, Ibrahim et al., 2019). The aqueous seed extract of *C. melo*, enriched in trypsin inhibitors, exhibited strong anti-angiogenic activity by suppressing the human umbilical vein endothelial cells (HUVEC) proliferation and motility in a dose-dependent manner (IC_50_ = 20 µg/mL). It also exhibited complete inhibition of the tube formation at 40 µg/mL, accompanied by downregulation of MMP-2, MMP-9, and vascular endothelial growth factor (VEGF) secretion, further supported by molecular docking with *α*V*β*3 integrin and VEGFR1 (Rasouli et al., 2017). The breast cancer cells (MC4-L2) implanted in mice demonstrated reduced angiogenesis, tumor necrosis, and significant dose-dependent decreases in tumor size following treatment with trypsin inhibitor (0.3 mg/mL) or the seed extract (0.4 mg/mL). The most pronounced effect was observed when combined with tamoxifen (Raja Soh, Hapidin, & Kasiram, 2024). Cucurbitacin B (**91**), abundant in the pedicels, also suppressed VEGF-induced HUVEC migration and tubulogenesis *in vitro* and controlled angiogenesis *in vivo* (Piao et al., 2018, Silva et al., 2020). Additional evidence showed that *C. melo* var. *conomon* fruit extracts (0.125 − 2.0 mmol/L) exerted anti-carcinogenic, anti-mutagenic, and antioxidant effects on colorectal cancer cells (RCM-1), largely attributed to sulfur-containing volatiles, particularly methylthioacetic acid (MTA). In addition, at ≥ 2 mmol/L, MTA induced apoptosis markers including DNA fragmentation, Caspase-3/7 activation, and poly (ADP-ribose) polymerase cleavage (Kamimura et al., 2023). Moreover, the cucurbitacins isolated from the stems (Table S1) exhibited strong cytotoxicity against lung (A549/ATCC) and liver (BEL-7402) cancer cells, with cucurbitacin B (**91**) showing the highest potency (IC_50_ = 0.01 − 0.008 µmol/L), followed by cucurbitacin A (**100**) and 7*β*-hydroxycucurbitacin B (**104**) (Chen, Qiang, Lou, & Zhao, 2009).

Other *Cucumis* species also demonstrated notable anti-cancer effects. The methanolic fruit extract of *C. prophetarum* and its fractions (*n*-hexane, ethyl acetate, and subfractions) exhibited potent cytotoxicity against breast (MCF-7, MDA-MB-231), colon (HCT-116), ovarian (A2780, A2780CP), and liver (HepG2) carcinoma cell lines, with IC_50_ values ranging from 0.35 µg/mL (MDA-MB-231) to 55.4 µg/mL (HepG2). The ethyl acetate subfractions showed even stronger activity (IC_50_ = 0.12 − 20.5 µg/mL), largely attributed to the cucurbitane-type triterpenes such as cucurbitacins B (**91**), E (**95**), D (**108**), hexanorcucurbitacin D (**119**), and cucurbitacin F 25-*O*-acetate (**125**). Likewise, cucurbitacin B (**91**) and its dihydro-derivative (**106**) displayed anti-proliferative activity against leukemia (KA3IT) and embryonic fibroblast (NIH3T3) cells (Alsayari et al., 2018, Ayyad et al., 2011). In addition, the methanolic fruit extract of *C. callosus* exerted a dose-dependent anti-tumor effect in Ehrlich ascites carcinoma (EAC)-bearing mice, with the pericarp and seed extracts showing stronger activity (IG_50_ = 235.08 and 273.17 µg/mL) than the whole fruit extract (Deepika et al., 2023, Panda et al., 2018). Similarly, the ethyl acetate fruit extract of *C. dipsaceus* displayed cytotoxicity against MCF-7 cells (IC_50_ = 16.05 µg/mL, comparable to doxorubicin), attributed to cucurbitacin D (**108**) and its dehydroxycucurbitacin D (**134**), which showed high binding affinities to human topoisomerase II*β* than etoposide *in silico* (Assefa et al., 2024).

### 5.6 Organ-protective activities

*Cucumis* species exhibit broad organ-protective effects, including neuroprotection against oxidative stress, hepatoprotection, nephroprotection, and gastroprotection (anti-ulcer, anti-diarrheal, and laxative activities), highlighting their potential as natural therapeutic agents.

#### 5.6.1 Neuroprotective activities

Neuroprotective activities of *Cucumis* plants have been reported through enzymatic inhibition, antioxidant effects, and cognitive enhancement. For example, *C. sativus* aqueous extracts inhibited key neuroenzymes, such as acetylcholinesterase, butyrylcholinesterase, and monoamine oxidase enzymes, and exhibited strong brain antioxidant activity (Oboh et al., 2017). In addition, the ethanolic extracts of *C. melo* var. *reticulatus* and *C. melo* var. *flexuosus* seeds (100 mg/kg, 60 d) exhibited sedative and anxiolytic effects without impairing motor coordination, while the leaf extract (60 − 120 mg/kg) ameliorated STZ-induced oxidative stress in the rat brains by lowering blood glucose, HbA_1C_, TNF-*α*, ILs, MDA, and Caspase-3, and increasing dopamine, melatonin, VEGF-A, SOD, and CAT activities (Ibrahim, 2017, Tuseef Sayyar et al., 2023). *C. trigonus* ethanolic extract (150 − 300 mg/kg) improved memory in scopolamine-induced amnesia, increased hippocampal acetylcholine, and showed antioxidant effects (Bharti & Bora, 2024). The coumarins (**269**, **271**, **273** − **275**, **277**) isolated from *C. bisexualis* fruits exhibited anti-acetylcholinesterase activity with IC_50_ values ranging from 11.23 to 89.69 µmol/L (Ma et al., 2018). Collectively, these findings support the neuroprotective potential of the genus, particularly in the management of cognitive impairment and Alzheimer’s disease.

#### 5.6.2 Hepatoprotective activities

*Cucumis* species have been traditionally used for liver disorders (Table 1), and some pharmacological studies confirm their hepatoprotective effects, largely attributed to triterpenoids, flavonoids, phenolics, and vitamins. The ethanolic fruit and aqueous seed extracts of *C. sativus* (500 mg/kg) protected against paracetamol- and arsenic-induced hepatotoxicity in rodents by normalizing liver biomarkers (SGOT, SGPT, GGTP, LPO, AST, ALT, ALP, SOD, CAT, GSH, GSH-Px, GR, and bilirubin) and improving histopathology (Idemudia and Enogieru, 2024, Khan et al., 2022). In traditional Chinese medicine, the pedicels of *C. melo* (*Pedicellus Melo*, Tian Gua Di), known for their bitter taste and strong emetic properties, are applied for chronic hepatitis and alcoholism. Clinical reports indicated its effectiveness in inducing alcohol aversion in 97% of patients. Moreover, cucurbitacins B (**91**) and E (**95**) (0.2 mg/kg, i.v.) normalized liver protein levels, prevented hepatic damage, and enhanced liver function through modulation of the AMPc/GMPc (cyclic adenosine monophosphate/cyclic guanosine monophosphate) ratio (Du et al., 1995, Miró, 1995). In addition, a significant activity has been reported for the fruits of *C. bisexualis*, which contain a wide array of hepatoprotective metabolites (Table S1). These include homoisoflavonoids (**258** − **262**, **264**), coumarins (**266** − **267**, **271** − **272**, **280**, **283**, **286** − **288**), quinones (**295** − **296**, **298**, **303**, **306**, **309**, **313**), flavonolignans (**324**, **326** − **327**), aurones (**335** − **336**, **339** − **340**), and biphenyls (**341** − **342**, **344**). These compounds demonstrated protective effects in HepG2, HL-7702, and L-O2 liver cell models against hepatotoxic agents such as H_2_O_2_, paracetamol, *D*-galactosamine, and CCl_4_. The hepatoprotective activities included lowering AST and ALT levels, enhancing cell survival (60%−71% *vs* 51% in paracetamol-injured HepG2), and repairing hepatotoxicity with moderate to significant inhibition (22%−66%), comparable to positive controls such as bicyclol and quercetin (Ma et al., 2024, Ma and Wei, 2021a, Ma and Wei, 2021b, Ma and Wei, 2023, Ma et al., 2020a, Ma et al., 2020b, Ma et al., 2020c, Ma et al., 2021, Ma et al., 2018).

Other *Cucumis* species have also demonstrated notable hepatoprotective effects. Oral administration of *N*-trisaccharide (**398**), at 25 − 50 mg/kg for 28 d, protected against CCl_4_-induced hepatotoxicity in rats by reducing DNA fragmentation, restoring hepatic architecture, suppressing apoptosis, and normalizing liver enzymes (Kavishankar, Moree, & Lakshmidevi, 2014). *C. ficifolius* methanolic root extracts (500 mg/kg) and the alkaloidal fraction of *C. metuliferus* fruits (200 mg/kg) demonstrated comparable hepatoprotective effects in CCl_4_-induced hepatitis in rats, as evidenced by reductions in ALT, AST, and ALP levels, along with histopathological confirmation of diminished lesions and necrosis. These protective effects were attributed to strong radical scavenging activity, thereby providing pharmacological support for their traditional hepatoprotective use (Araya et al., 2019, Šeregelj et al., 2022). Additionally, the flavonoid-rich methanolic and aqueous extracts of *C. dipsaceus* fruits protected HepG2 cells against H_2_O_2_-induced injury at 500 µg/mL, while *in vivo* administration (200 mg/kg) improved SGOT, SGPT, SOD, CAT, and GSH levels, further supported by histopathological evidence (Lata and Mittal, 2017b, Lata and Mittal, 2017).

#### 5.6.3 Nephroprotective activities

*Cucumis* species also demonstrate nephroprotective potential. For instance, the aqueous seed and ethanolic pulp extracts of *C. sativus* (500 mg/kg) ameliorated arsenic-, alloxan-, and cadmium-induced nephrotoxicity and diabetic nephropathy by normalizing renal biomarkers (urea, uric acid, and creatinine) and antioxidant status in rats (Ofoego et al., 2019). Similarly, the ethanolic leaf extract of *C. melo* var. *flexuosus* (120 mg/kg, 30 d) alleviated STZ-induced renal injury in diabetic rats by reducing oxidative stress, inflammation, and apoptosis, markedly lowering kidney injury molecule-1 (KIM-1), TBARS, TNF-*α*, IL-6, and Caspase-3, while enhancing VEGF and antioxidant enzymes (Abd El-Maksoud, 2019). *N*-Trisaccharide (**398**) isolated from *C. prophetarum* fruits (25 − 50 mg/kg) protected diabetic rats from STZ-, NIC-, and CCl_4_-induced nephrotoxicity, reducing apoptosis and restoring renal function markers (Kavishankar, Moree, & Lakshmidevi, 2014).

*Cucumis* species also exhibit promising anti-urolithiatic and anti-nephrolithiatic properties that complement their nephroprotective effects, supporting their traditional use in managing calcium oxalate stones. In the ethylene glycol-induced nephrolithiasis models, the methanolic and ethanolic seed extracts of *C. melo* var. *inodorus* (100 − 600 mg/kg) significantly reduced hyperoxaluria, normalized urinary calcium and oxalate levels, and aided in the dissolution and excretion of calcium oxalate crystals. These effects were comparable to, or in some cases exceeded, those of standard therapies such as cystone and potassium citrate (Afzal et al., 2021, Eidi and Ashjazadeh, 2023). Similarly, *C. callosus* ethanolic fruit extract (250 mg/kg) alleviated stone formation, improved renal parameters (kidney index, crystal deposition, histopathological damage, and inflammation scores), and restored oxidant/antioxidant balance (Choudhary et al., 2023). Collectively, these findings, together with the antioxidant, anti-inflammatory, analgesic, and diuretic effects of both plants, highlight their potential as natural therapeutic agents for nephrolithiasis and related renal dysfunctions.

#### 5.6.4 Gastroprotective activities

*Cucumis* species have traditionally been used in the management of GIT disorders, with ethnomedicinal records and limited pharmacological studies providing supportive evidence of their efficacy. Notably, some species demonstrated significant anti-ulcer activity. For example, the aqueous extracts of *C. sativus* fruits (500 − 1 000 mg/kg) exhibited strong anti-ulcer activity in acetic acid-, indomethacin-, and pyloric ligation-induced models, reducing lesion index (by 25.8%−95.5%), gastric acidity, and LPO, while enhancing pH, SOD, and CAT levels. At 500 mg/kg, the extract also alleviated ulcerative colitis by lowering ulcer area, MPO activity, and inflammatory mediators. Moreover, the methanolic seed extracts and their active metabolite “9*β*-methyl-19-norlanosta-5-ene-type glycoside” demonstrated potent gastroprotective and antioxidant effects in stress- and non-steroidal anti-inflammatory drugs (NSAIDs)-induced ulcer models (Gill and Bali, 2012, Idemudia and Enogieru, 2024, Khan et al., 2022). *C. melo* extracts also demonstrated notable gastroprotective effects, providing up to 74% protection against NSAID-induced ulcers by reducing gastric acidity and mucosal damage, potentially through angiogenic mechanisms (CD31 upregulation). Among them, the aqueous pulp and methanolic seed extracts of *C. melo* var. *inodorus* promoted gastric mucosal repair. This effect could be attributed to phytosterols such as codisterol (**15**) and *β*-sitosterol (**24**), which demonstrated strong binding affinities to PGE_2_ receptors in docking studies, thereby confirming their gastroprotective role (Adebayo-Gege et al., 2022, Adebayo-Gege et al., 2023, Gill et al., 2010, Silva et al., 2020).

In addition to the anti-ulcer activity, *Cucumis* species also exhibit potent anti-diarrheal effects. The ethanolic seed extracts of *C. sativus* and *C. melo* (150 − 300 mg/kg) significantly reduced charcoal meal transit and inhibited castor oil-induced diarrhea in mice, showing anti-peristaltic and anti-secretory effects comparable to loperamide and verapamil. The methanolic leaf extracts (250 − 500 mg/kg) further supported this activity through anti-secretory mechanisms (Wahid et al., 2022, Wahid et al., 2024). Likewise, the aqueous and methanolic extracts of *C. dipsaceus* leaves and fruits (200 − 400 mg/kg) inhibited diarrhea in a dose-dependent manner and displayed additional anti-inflammatory actions through prostaglandin suppression (Kimathi, Maitho, Mbaria, & Moriasi, 2022).

The genus is also recognized for its traditional laxative and purgative properties, which have been validated by limited pharmacological evidence. The rectal administration of the ethanolic extracts of *C. melo* pedicels (6.5 − 26 mg/kg) enhanced fecal output, accelerated gastric emptying, and improved intestinal transit in rodents in a dose-dependent manner. In rats, doses of 8 − 16 mg/kg increased proximal colon contractility, while *ex vivo* assays confirmed muscarinic agonist-mediated prokinetic effects. These findings, together with the wide therapeutic window (4 − 400 mg/kg), support the traditional use of *Pedicellus Melo* as a rectal suppository for constipation and abdominal distension in the Chinese medicine (Gao et al., 2012).

### 5.7 Antimicrobial and antiparasitic activities

Microbial and parasitic infections remain major global health concerns, particularly with the increasing of drug resistance and treatment failure (Assefa et al., 2024, Yoon et al., 2015). *Cucumis* species exhibit antimicrobial and antiparasitic activities due to diverse phytochemicals that synergistically disrupt microbial membranes, inhibit nucleic acid synthesis, and suppress parasite growth, supporting their ethnomedicinal relevance. The ethanolic extract of *C. sativus* fruit peels exhibited antibacterial activity against *Escherichia coli*, *Streptococcus mutans*, and *Pseudomonas aeruginosa*, with inhibition zone diameters (IZDs) ranging from 6.70 to 11.36 mm (Anjani, Srivastava, & Mathur, 2023). However, the ethanolic and chloroform leaf and stem extracts (80 µg/disc) showed moderate antifungal activity (IZDs = 1.50 − 4.40 mm) against *Aspergillus niger*, *Blastomyces dermatitides*, *Candida albicans*, and *Pityrosporum ovale*. Among them, *A. niger* was the most susceptible (Idemudia and Enogieru, 2024, Khan et al., 2022). The chloroform fraction obtained from the methanolic stem extract inhibited Gram-positive and negative bacteria. This effect is attributed to sphingolipids (**399** − **401**), which blocked mycelial growth by 23%−100% at 100 µg/mL, with sphingolipid (**399**) being the most active, particularly against *Pythium aphanidermatum* (IC_50_ = 15.30 µg/mL) (Tang et al., 2010). The methanolic extract of *C. melo* var. *reticulatus* seeds showed strong antibacterial activity against *P. aeruginosa*, and moderate effects against *E*. *coli* and *Geotrichum candidum* (Ibrahim, 2010). Both *C. melo* var. *agrestis* and *C. melo* var. *momordica* methanolic extracts exhibited varying antibacterial effects against *Staphylococcus aureus*, *Pseudomonas fluorescens*, *Bacillus coagulans*, and *Klebsiella pneumoniae*. Notably, *C. melo* var. *agrestis* demonstrated the strongest activity, particularly against *S. aureus* (IZD = 32.30 mm) (Singh, Kaur, Devashree, Kaur, & Gupta, 2022).

Other *Cucumis* species have also demonstrated notable antimicrobial activities. The methanolic and *n*-hexane extracts of *C. prophetarum* roots, along with phytoconstituents (**23**, **36**, **132** − **133**), exhibited antibacterial activity against *E. coli*, *S. aureus*, *Bacillus subtilis*, and *Salmonella typhimurium* (IZDs = 13.60 − 15.00 mm), reinforcing the roots’ traditional use against bacterial infections. Cucurbitacin-1 (**132**) exhibited higher activity particularly against *E. coli*, further supported by *in silico* docking to bacterial DNA gyrase (Galma et al., 2021). The crude methanolic root extract of *C. ficifolius* and cucurbitacin B (**91**) were active against Gram-positive bacteria, particularly *B. subtilis* and *S. aureus* (Nigussie & Ashenef, 2020). The extracts of *C. anguria* hairy roots and non-transformed roots (100 mg/disc) also displayed broad antibacterial activity against *E*. *coli*, *P*. *aeruginosa*, and *S*. *aureus* (IZDs = 19.50 − 25.80 mm) and antifungal effects against *A. niger* and *Fusarium oxysporum* (IZDs = 16.50 − 22.00 mm) (Yoon, Chung, & Thiruvengadam, 2015). Moreover, *C. dipsaceus* fruit and leaf extracts (100 µg/mL) exhibited broad antibacterial activity against *B. subtilis*, *E. coli*, *P. aeruginosa*, and *Salmonella enteritidis*, with IZDs of 7.10 − 20.33 mm. The petroleum ether fruit extract (400 µg/mL) was most effective against *P*. *aeruginosa* and *Streptococcus pyogenes*, while the methanolic and ethyl acetate extracts demonstrated strong activity against *E*. *coli* and *S*. *aureus* only. The aqueous leaf extract also showed potent antifungal activity against *C. albicans* (IZD = 15.00 mm) (Kimathi, Maitho, Mbaria, & Moriasi, 2022). In addition, the ethanolic fruit extract of *C. pubescens* (100 µL) exhibited antibacterial activity (IZDs = 9.00 − 23.00 mm) against ampicillin-resistant strains, including *E*. *coli*, *S*. *aureus*, *Proteus vulgaris*, and *Serratia marcescens*, with the strongest effect observed against *P. aeruginosa* (Sundari & Kavitha, 2024). The isolated metabolites, including *α*-spinasterol (**36**) and triterpenoids (**108**, **113**, **134**, **147**, **164**), also displayed IZDs of 6.10 − 15.00 mm (Assefa et al., 2024).

*Cucumis* species have also been traditionally used as anthelmintic and anti-malarial agents, mainly due to cucurbitacins (Gómez-García, Campos, Aguilar, Madureira, & Pintado, 2020). For example, the ethanolic seed extracts of *C. sativus* and *C. melo* exhibited 100% and 75% anthelmintic efficacy, respectively, in mice infected with *Hymenolepis nana*, surpassing the efficacy of piperazine (80%). The underlying mechanism was suggested to involve lectins from fruit exudates, which may act as defense proteins against chitin-containing parasites (Khan et al., 2022, Mukherjee et al., 2013). Interestingly, the methanolic root extract of *C*. *ficifolius* and its fractions (400 mg/kg) suppressed *Plasmodium berghei* parasitemia in mice (40%−65%) better than standard drug chloroquine (25 mg/(kg·d)). Specifically, the crude extract and chloroform fraction were found to have the longest longevity (15.4 and 13.4 d). Collectively, these findings support the ethnomedicinal use of its roots against malaria (Bizuneh et al., 2023).

*C. africanus*-derived nemafric-BL, a cucurbitacin B (**91**)-enriched phytonematicide from the fermented fruit extracts, effectively suppressed root-knot nematodes across cropping systems. Similarly, *C*. *myriocarpus*-based nemarioc-AL, rich in cucurbitacin A (**100**), reduced gall formation and nematode populations by inhibiting egg hatching (Maja et al., 2022, Shaik et al., 2017). These findings support the genus’s nematicidal potential, though further studies are needed to validate applications in animals and humans.

### 5.8 Other activities

Beyond the major pharmacological effects, *Cucumis* species have also been reported to exert several minor biological activities, as summarized in Fig. 7. These effects, however, are mostly limited to one or two species and are supported by a small number of studies, highlighting the necessity for more comprehensive investigations to establish their therapeutic significance.

#### 5.8.1 Anti-viral activities

The alkaloidal components of *C. metuliferus* fruits administered intraperitoneally at 600 mg/kg showed marked antiviral activity, reducing hemorrhagic lesions and Newcastle disease virus (NDV-K and NDV-I) symptoms in chicks. In hepatitis B virus (HBV)-infected rats, the oral doses of 50 − 200 mg/kg significantly decreased serum liver biomarkers (ALP, ALT, AST). Moreover, they demonstrated a favorable safety profile in chicken fibroblast cells and exhibited effective antiviral activity against the infectious bursal disease virus (IBDV) at concentrations ranging from 6.125 mg/mL to 100 mg/mL. These outcomes, therefore, support the traditional use of the fruits against HBV, HIV/AIDS, and other viral infections (Šeregelj et al., 2022).

#### 5.8.2 Wound-healing effects

Topical oil application of *C. melo* var. *inodorus* and *C. melo* var. *cantalupensis* seeds (0.52 µL/mm^2^, twice daily for 14 d) accelerated excisional wound healing in rats, with *C. melo* var. *inodorus* showing superior efficacy compared to pumpkin seeds oil. The effect was linked to modulation of the AGE/RAGE pathway, activation of Nrf2/HO-1, suppression of pro-inflammatory mediators (TNF-*α*, NF-*κ*B, NLRP3), and downregulation of connexin-43 (CX-43), supported by its high linoleic acid (65.9%), squalene, and plant sterols (Emad et al., 2024).

#### 5.8.3 Cosmetic uses

The fresh fruits and seeds of *C. sativus* have been traditionally used for treating hyperpigmentation and cleansing. Nowadays, the fruits are widely included in skin-care formulations for soothing irritation, reducing swelling, and providing a cooling effect. The fruits are mainly composed of water (96.4%) but also contains calcium, phosphorus, iron, and vitamins B and C. Its lyophilized juice, rich in vitamin C (3.5% *w/w*), showed potent anti-hyaluronidase and anti-elastase (IC_50_ = 20.98 and 6.14 µg/mL, respectively) activities and demonstrated moderate photoprotection, with a sun protection factor (SPF) of (0.67 ± 0.54) at 200 µg/mL, supporting its cosmetic potential. The pulp contains high lactic acid (**403**), widely used in dermatology to thin the stratum corneum and treat conditions such as dry skin, ichthyosis, follicular and seborrheic keratosis, and solar keratosis. Moreover, (*E*,*Z*)-2,6-nonadienal, a major volatile compound, acts as a non-competitive tyrosinase inhibitor. The methanolic extracts of *C. sativus* leaves and stems inhibited melanogenesis in B16 melanoma cells through tyrosinase downregulation. These effects could be attributed to the lignan (**319**) and carotenoid (**405**), which significantly suppressed melanin synthesis (IC_50_ = 270.8 and 170.7 µmol/L, respectively). Toxicological evaluations confirmed safety of the fruits and seeds regarding genotoxicity, carcinogenicity, irritation, sensitization, phototoxicity, cross-allergenicity, and ocular effects. Collectively, these properties highlight *C. sativus* fruits as promising anti-wrinkle agents (Fiume et al., 2014, Mukherjee et al., 2013).

Parallel evidence highlighted *C. melo* as a source of antioxidant molecules, particularly vitamins A and C, gallic acid (**349**), and caffeic acid (**354**), which neutralize free radicals, alleviate oxidative stress, and inhibit inflammatory pathways, thereby protecting the skin from aging (Vouldoukis et al., 2004). *In vivo*, oral administration of a multi-plant extract containing *C. melo* (125 − 500 mg/kg, eight weeks) to mice alleviated UV-induced photoaging, enhanced collagen architecture, and reduced skin damage (Xie et al., 2022). Moreover, cucurbitacins B (**91**), D (**108**), and dihydrocucurbitacin D (**112**) isolated from *C. melo* demonstrated skin-whitening potential via tyrosinase inhibition, supporting its cosmetic potential as a natural skin-whitening agent (Chen, Chiu, Nie, Cordell, & Qiu, 2005).

#### 5.8.4 Anti-allergic activities

Bryonolic acid (**156**), isolated from *C. sativus* and *C. melo*, exhibited strong anti-allergic potential in rodents, with 600 mg/kg producing pronounced effects. It inhibited homologous passive cutaneous anaphylaxis by 23%−80% at 300 − 600 mg/kg, and significantly suppressed type IV hypersensitivity reactions, including contact dermatitis and autoimmune inflammation (Akiyama & Hayashi, 2002).

#### 5.8.5 Smooth muscle relaxant effects

The seed extracts of *C. sativus* and *C. melo* (1.0 − 3.0 mg/mL) showed strong anti-spasmodic activity by reversing spontaneous jejunal contractions and completely inhibiting K^+^-induced contractions in rabbit tracheal and bladder smooth muscles, comparable to verapamil (1.0 µmol/L). Similar bronchodilatory effects were observed in human, rat, and pig tissues, likely mediated by high levels of secondary metabolites (263.53 − 783.02 µg/g), including flavonoids (**176**, **230**, **238**, **243** − **244**) and phenolic acids (**353** − **354**). These findings highlight their potential therapeutic applications in asthma and enuresis (Wahid et al., 2022, Wahid et al., 2024).

#### 5.8.6 Anti-hypothyroidism activity

In healthy and hypothyroid rat models, the methanolic extract of *C. melo* fruit peel (100 mg/kg, for 10 d) significantly increased or restored serum triiodothyronine (T3) and thyroxin (T4) levels, while also reducing hepatic, cardiac, and renal lipid peroxidation. These findings suggest its potential to stimulate thyroid function and protect vital organs from oxidative stress (Parmar and Kar, 2009, Silva et al., 2020).

## 6 Clinical studies and applications of *Cucumis* species

*Cucumis* species showed considerable pharmacological promise, yet clinical validation is inadequate, with evidence largely confined to preclinical studies. The absence of human trials leaves fundamental gaps regarding efficacy, safety, mechanisms, and dosage, highlighting the need for rigorous clinical research. Nonetheless, their incorporation into pharmaceutical and cosmetic products reflects their growing therapeutic and commercial relevance despite limited clinical evidence.

### 6.1 Clinical applications in cosmetics and skin health

*Cucumis* species demonstrate promising dermatological and cosmetic potential owing to their antioxidant and anti-inflammatory activities. Several cucumber- and melon-based formulations are already available commercially; however, further research is required to explore additional applications, particularly in wound-healing and anti-inflammatory skin care. A pilot comparative study in 25 aged subjects, *C. sativus* supplementation enhanced plasma vitamin C and phenolics levels, while reducing oxidative stress, hemolysis, and DNA damage, supporting its role in delaying skin aging and deterioration (Ji et al., 2015). Topically, a well-tolerated 20% cucumber-based cream prevented severe radiation-induced dermatitis and promoted skin recovery in 30 breast cancer patients receiving radiotherapy. By one-month follow-up, these patients showed improved skin healing, with no cases of dermatitis (Thanthong et al., 2020). Clinically, a case report described a 42-year-old male vitiligo patient who achieved gradual re-pigmentation after applying sulfur powder with cucumber fruit slices. After 12 months, lesions showed marked recovery, and complete remission was sustained for 21 years without relapse (Liu, Wang, Zhang, Guo, & Chen, 2019).

*C. melo* extracts have also been investigated for pigmentation disorders. In a randomized, triple-blind clinical trial, a well-tolerated 10% herbal cream containing *C*. *melo* var. *inodorus* seed extract significantly reduced melasma severity in 32 women, matching the efficacy of 4% hydroquinone with minimal side effects (Mahjour et al., 2020). Similarly, a 12-week clinical study in 55 healthy adults (45 − 60 years) reported that a multi-plant formulation containing 70 mg of *C. melo* extract improved skin hydration, elasticity, and barrier function, while reducing pigmentation and enhancing brightness, with participants noting smoother and healthier skin (Xie et al., 2022). In addition, double-blind clinical studies demonstrated that SOD-rich melon formulations provided strong photoprotective and anti-aging effects. Participants receiving 1 000 IU-NBT of SOD daily for two weeks showed reduced oxidative markers and DNA damage. In 88 healthy adults, a SOD-rich concentrate, applied topically (12 U SOD/cm^2^) or orally (280 U SOD/d), enhanced photoprotection by increasing minimal erythema dose, boosting antioxidant enzymes, reducing sunburn cells (by 72.5%), and normalizing UV-induced melanin overproduction (Egoumenides et al., 2018, Muth et al., 2004). Advanced products such as GliSODin®, a commercially available formulation combining melon extract with wheat protein, have demonstrated the ability to elevate SOD, CAT, and GSH-Px activities in human cells, thereby conferring potent antioxidant, anti-inflammatory, and anti-aging effects.

### 6.2 Treatment of osteoarthritis

A randomized, double-blind, controlled trial in 122 patients with moderate knee osteoarthritis (aged 40 − 75 years) demonstrated that oral administration of Q-Actin™, an aqueous *C. sativus* extract standardized to contain ≥ 1% idoBR1 (**397**), at 10 mg twice daily significantly improved clinical outcomes compared to glucosamine-chondroitin (1.35 g, twice daily). The Western Ontario and McMaster Universities Osteoarthritis Index (WOMAC) decreased by 29% and 70% at days 30 and 180 in the Q-Actin™ group, versus 14% and 33% in the comparator group, confirming its superior efficacy in reducing pain, stiffness, and functional impairment (Nash, Azantsa, Sharp, & Shanmugham, 2018). Similar findings were reported in a randomized controlled trial involving 55 osteoarthritis patients, where treatment with *C. sativus* extract (20 mg/d, eight weeks) significantly reduced plasma levels of pro-inflammatory and cartilage-degrading biomarkers, including IL-1*β* and MMP-3 (Pérez-Piñero et al., 2023). The mechanistic studies suggest that idoBR1 (**397**) exerts anti-inflammatory and chondroprotective effects by inhibiting 5-lipoxygenase (5-LOX) activity, TNF-*α* and IL-1*β* generation, enhancing chondrocyte proliferation and extracellular matrix synthesis, and downregulating MMP-3 expression (Nash et al., 2020). Safety assessments revealed no adverse effects, with negative mutagenicity and genotoxicity tests supporting its tolerability (Kothari, Saravana, Muthusamy, Mozingo, & Soni, 2018).

### 6.3 Treatment of metabolic disorders

The therapeutic potential of *Cucumis* species in metabolic disorders, particularly diabetes mellitus and hyperlipidemia, has been clinically supported. In a trial involving ten hospitalized diabetic patients (aged 46 − 75 years), administration of 240 g of fresh juice from *C. melo* var. *reticulatus* or *C. melo* var. *conomon* significantly lowered blood glucose levels, with the most pronounced effect observed in fully ripe *C. melo* var. *conomon* (Sasaki et al., 2020). In another randomized, double-blind, placebo-controlled trial, 24 hyperlipidemic patients received *C. sativus* seed extract (500 mg/d) for six weeks. This safe treatment significantly reduced TC, LDL, and TG, accompanied by a slight decrease in BMI, while increasing HDL. The lipid-lowering effects of the extract are thought to be mediated by linoleic acid and phytosterols that regulate lipid metabolism, suggesting its potential as a functional food supplement for cardiovascular health (Soltani et al., 2017).

### 6.4 Treatment of hypertension

*C. sativus* has been evaluated for its antihypertensive potential in elderly patients. In a study conducted on 35 subjects, the daily intake of 200 g fruit juice mixed with 100 mL water twice for three days significantly reduced systolic (149.68 mmHg *vs* 136.65 mmHg) and diastolic (95.99 mmHg *vs* 80.09 mmHg) blood pressure. These effects are likely attributable to the high potassium, magnesium, and phosphorus contents, which promote vasodilation, reduce peripheral resistance, and enhance diuresis, thereby lowering blood pressure (Negara, Erna, & Anna, 2018).

### 6.5 Treatment of nephrolithiasis

In a single-blind, randomized, active-controlled trial involving 44 patients with renal calculi measuring 5 − 10 mm, *C. melo* seed decoction administered twice daily for 45 d achieved 65% complete stone clearance, significantly improved symptoms, and showed no adverse effects. These outcomes are likely due to the seeds’ diuretic, anti-inflammatory, and lithotriptic properties, supporting their traditional use (Rashid, Rather, & Bhat, 2024).

### 6.6 Treatment of oxidative stress and fatigue

In a randomized, double-blind, placebo-controlled trial, 41 healthy men have received *C. melo* concentrate (40 mg/d, equivalent to 560 U SOD/d) for eight weeks during moderate exercise. This overall well-being supplementation enhanced erythrocyte resistance to oxidative damage, reduced inflammation (lower C-reactive protein levels), and improved fatigue resistance, and recovery, as assessed by the SF-36® survey and Prevost fatigue scale (Saby, Gauthier, Barial, Egoumenides, & Jover, 2020).

## 7 Toxicology of *Cucumis* species

Human toxicities associated with *Cucumis* species are relatively uncommon. However, a fatal case was reported in Kenya following consumption of a decoction of *C. dipsaceus* fruits, explained by its high levels of cucurbitacins B (**91**) and D (**108**) (Njoroge and Newton, 1994, Olarewaju et al., 2021). Another manifestation involves *C. melo* allergy, which is less prevalent than previously assumed, with only 36% of suspected cases confirmed by double-blind placebo-controlled food challenge. This allergy is often linked to pollen cross-reactivity and typically presents as oral allergy syndrome, characterized by itching, tingling, and swelling of the lips, mouth, and throat. Less frequently (19.7% of cases), the patients experience skin, gastrointestinal, or respiratory symptoms, while severe reactions such as anaphylaxis remain rare (Figueredo et al., 2003).

The genus is notable for its content of cytotoxic bitter compounds known as cucurbitacins, the primary toxic principles, whose extreme bitterness generally prevents excessive ingestion (Enslin, 1954). Cucurbitacins exhibited purgative, emetic, cytotoxic, insect antifeedant, and antifertility effects in female mice (Afifi et al., 1999, Miró, 1995). They possess narrow safety margins (2.0 − 12.5 mg/kg), with cucurbitacins A (**100**), B (**91**), C (**77**), D (**108**), E (**95**), and I (**123**) being the most toxicologically implicated (Enslin, 1954, Omokhua-Uyi and Van Staden, 2020). Specifically, cucurbitacins A (**100**) and B (**91**) showed intraperitoneal LD_50_ values of 1.0 − 2.0 mg/kg in mice and rats, while in cats, fatal outcomes occurred at 0.3 − 0.7 mg/kg. In contrast, cucurbitacin C (**77**) recorded an LD_50_ of 0.8 mg/kg in mice but was nonlethal at up to 4.0 mg/kg. These compounds consistently caused blood vessel engorgement, respiratory distress, and acute pulmonary congestion with edema (David & Vallance, 1955).

In South Africa and Australia, the ripe fruits, leaves, and roots of *C. africanus*, *C. myriocarpus*, and *C. leptodermis*, although traditionally used as emetics and purgatives, have been associated with poisoning and death in livestock, including cattle and horses. For instance, the ingestion of *C. myriocarpus* fruits caused pulmonary congestion, edema, gastrointestinal hemorrhage, hepatomegaly, hepatocellular vacuolation, and patches of myocardial degeneration and necrosis, leading to death within six hours, with lethal doses as low as 20 − 60 g/kg. Toxicity is confined to the bitter pulp, which at lower dose (20 − 40 g/kg) caused persistent diarrhea, neutrophilia, and elevated liver enzymes in cattle, while poisoning cases have also been reported in children, underscoring the need for extreme caution (Enslin, 1954, Mukherjee et al., 2022, Shaik et al., 2017).

Experimental studies further confirm the toxicity of cucurbitacins and related bitter principles. For example, the trilactones “cucumin (molecular formula: C_27_H_40_O_9_)” and “leptodermin (molecular formula: C_27_H_38_O_8_)”, isolated from *C. africanus*, *C. myriocarpus*, and *C*. *leptodermis*, caused nasal, throat, and eye irritation upon inhalation. In rabbits, a minimum lethal dose (MLD) of 1.0 − 2.0 mg/kg caused death within two hours, associated with pulmonary edema and gastrointestinal inflammation, while in guinea pigs, similar effects with hyperemia were observed at an oral dose of 25 mg/kg (Rimington, 1933, Rimington, 1935). These species are also packed with higher concentrations of cucurbitacins A (**100**) and B (**91**), with intravenous MLDs of 0.5 and 0.7 mg/kg in rabbits, respectively (Enslin, 1954).

Furthermore, several toxicological investigations have been conducted on other *Cucumis* species. The i.v. administration of *C. melo* pedicel extract (2.0 mg/kg), rich in cucurbitacins B (**91**), E (**95**), and D (**108**), induced rapid mortality in animals, with 94% of mice dying within 12 h due to vascular leakage leading to pleural effusion, ascites, and severe cerebral edema (Miró, 1995, Yuan et al., 2019). The methanolic extract of *C. callosus* fruit pericarp, rich in cucurbitacin B (**91**), caused teratogenicity in zebrafish embryos at 240 − 360 µg/mL, producing dose-dependent edema, depigmentation, and abnormal development through CYP_450_-mediated apoptosis (Panda, Jena, Kalyani, & Panigrahy, 2018). *C. metuliferus* fruits are toxic in the unripe state, where daily oral exposure to 500 − 1 000 mg/kg for 28 d caused hepatotoxicity and nephrotoxicity in rats, as evidenced by altered biochemical and hematological markers (Olarewaju et al., 2021, Šeregelj et al., 2022). Similarly, a combined extract of *C. metuliferus* and *C. zeyheri* fruits induced liver and kidney toxicity in rats at 1 000 mg/kg, although no adverse effects were observed in chickens (Mukherjee et al., 2022). Acute oral toxicity and death were also reported for *C. dipsaceus* fruits at 500 mg/kg in rats (Lata and Mittal, 2017b, Lata and Mittal, 2017). Toxicity has also been noted from *C. anguria* fresh juice, which exhibited higher lethality in rats (LD_50_ = 1.6 mg/kg) in its unprocessed form, whereas boiling markedly reduced its toxicity (Mukherjee et al., 2022, Omokhua-Uyi and Van Staden, 2020).

## 8 Conclusions and future perspectives

This review provides an integrated overview of the traditional ethnomedicinal uses, phytochemistry, pharmacological activities, clinical applications, and toxicity of genus *Cucumis*. Despite its long history of medicinal and nutritional applications, significant challenges hinder the scientific validation and therapeutic exploitation of this genus. Taxonomic ambiguity, due to the close similarity between conspecific species and intraspecific varieties, continues to compromise data reliability. Accurate taxonomy, supported by chemotaxonomic markers, is therefore essential for strengthening future phytochemical and pharmacological investigations.

Although more than 428 phytochemicals have been reported up to December 2025, the biological relevance of many remains poorly understood. Research has largely focused on cucurbitacins and phenolic compounds, yet the mechanisms of action underlying their pharmacological effects are still insufficiently understood and require further elucidation. Moreover, while the traditional uses of *Cucumis* species are often based on multi-component interactions, the modern studies typically examine isolated constituents. Future investigations should therefore explore synergistic effects among steroids, triterpenoids, flavonoids, phenolics, and other secondary metabolites, as well as their collective contribution to the bioactivity.

Despite of expanding current researches, several key gaps remain unaddressed. For instance, the essential oils from aromatic *Cucumis* species are underexplored, particularly the biosynthetic pathways of sulfur-containing volatiles, the influence of environmental factors on aroma profiles, and the poorly understood individual and combined roles of volatile compounds, which could be harnessed to develop novel aromatic cultivars with enhanced qualities. Clinical studies are critically lacking, and well-controlled trials are urgently needed to assess both the efficacy and safety. Establishing standardized authentication protocols, validated analytical methods, and robust quality control criteria for extracts and formulations is also necessary to ensure reproducibility and comparability across studies. Furthermore, rigorous toxicological assessments, including chronic toxicity, genotoxicity, carcinogenicity, and reproductive toxicity, are vital, especially when high doses or enriched phytoconstituents are used medicinally.

Looking forward, the multidisciplinary approaches integrating taxonomy, metabolomics, molecular biology, and pharmacology will be essential to advance the field. Research focus should be directed to the underutilized *Cucumis* species, which represent untapped sources of novel bioactive compounds. Strengthening mechanistic studies, implementing standardized quality measures, and conducting clinical research will collectively bridge the gap between traditional knowledge and modern evidence, paving the way for the rational development of Cucumis-based therapeutic agents.

## CRediT authorship contribution statement

**Hesham M. El-Sayed:** Conceptualization, Methodology, Data curation, Investigation, Visualization, Writing – original draft, Writing – review & editing. **Engy A. Mahrous:** Conceptualization, Supervision, Investigation, Visualization, Writing – review & editing. **Dalia M. Rasheed:** Conceptualization, Supervision, Investigation, Visualization, Writing – review & editing. **Essam Abdel-Sattar:** Conceptualization, Supervision, Investigation, Visualization, Project administration, Writing – review & editing.

## Declaration of Competing Interest

The authors declare that they have no known competing financial interests or personal relationships that could have appeared to influence the work reported in this paper.

## References

[b0005] Abd El-Fattah H., Zaghloul A.M., Halim A.F., Waight E.S. (1989). Cucurbitacins and steroids from *Cucumis callosus* (Rottl) Cong. Acta Pharmaceutica.

[b0010] Abd El-Maksoud M. (2019). Effect of *Cucumis melo* var. *flexuosus* leaves extract on renal oxidative injury and inflammation in diabetic male albino rats. *Egyptian*. Journal of Zoology.

[b0015] Abifarin T.O., Afolayan A.J., Otunola G.A. (2019). Phytochemical and antioxidant activities of *Cucumis africanus* L. f.: A wild vegetable of South Africa. Journal of Evidence-Based Integrative Medicine.

[b0020] Adebayo-Gege G., Alicha V., Omayone T.O., Nzekwe S.C., Irozuoke C.A., Ojo O.A. (2022). Anti-atherogenic and cardio-protective properties of sweet melon (*Cucumis melo*. L. *Inodorus*) seed extract on high fat diet induced obesity in male wistar rats. BMC Complementary Medicine and Therapies.

[b0025] Adebayo-Gege G., Uthman Z.S., Adams M.D., Florence T., Haruna D.U., Audu N.M. (2023). Molecular docking and anti-ulcerative potential of *Cucumis* (L. *Inodorous*) on ibuprofen induced gastric ulceration in male wistar animals. Biomedicine & Pharmacotherapy.

[b0030] Afifi M.S., Ross S.A., Elsohly M.A., Naeem Z.E., Halaweish F.T. (1999). Cucurbitacins of *Cucumis prophetarum* and *Cucumis prophetarum*. Journal of Chemical Ecology.

[b0035] Afolayan A.J., Mbaebie B.O. (2010). Ethnobotanical study of medicinal plants used as anti-obesity remedies in Nkonkobe Municipality of South Africa. Pharmacognosy Journal.

[b0040] Afzal M., Alharbi K.S., Alzarea S.I., Quazi A.M., Zafar A., Patel D.M. (2021). Methanolic extract of *Cucumis melo* attenuates ethylene glycol-induced nephrolithiasis in Wistar rats. Urolithiasis.

[b0045] Ahmed H.M. (2016). Ethnopharmacobotanical study on the medicinal plants used by herbalists in Sulaymaniyah Province, Kurdistan. Iraq. Journal of Ethnobiology and Ethnomedicine.

[b0050] Akihisa (ne Itoh) T., Ghosh P., Thakur S., Rosentein F.U., Matsumoto T. (1986). Sterol compositions of seeds and mature plants of family Cucurbitaceae. Journal of the American Oil Chemists’ Society.

[b0055] Akihisa T., Inada Y., Ghosh P., Thakur S., Rosenstein F.U., Tamura T. (1988). Compositions of triterpene alcohols of seeds and mature plants of family Cucurbitaceae. Journal of the American Oil Chemists’ Society.

[b0060] Akihisa T., Kimura Y., Kasahara Y., Kumaki K., Thakur S., Tamura T. (1997). 7-Oxodihydrokarounidol-3-benzoate and other triterpenes from the seeds of Cucurbitaceae. Phytochemistry.

[b0065] Akihisa T., Thakur S., Rosenstein F.U., Matsumoto T. (1986). Sterols of Cucurbitaceae: The configurations at C‐24 of 24‐alkyl‐Δ^5^‐, Δ^7^‐ and Δ^8^‐ sterols. Lipids.

[b0070] Akiyama K., Hayashi H. (2002). Arbuscular mycorrhizal fungus-promoted accumulation of two new triterpenoids in cucumber roots. Bioscience, Biotechnology, and Biochemistry.

[b0075] Akjhisa T., Shimizu N., Ghosh P., Thakur S., Rosenstein F.U., Tamura T. (1987). Sterols of the Cucurbitaceae. Phytochemistry.

[b0080] Aljohani O.S. (2022). Phytochemical evaluation of *Cucumis prophetarum*: Protective effects against carrageenan-induced prostatitis in rats. Drug and Chemical Toxicology.

[b0085] Al-Rehaily A.J., Al-Yahya M.A., Mirza H.H., Ahmed B. (2002). Cucumidisecosterol: A new diseco-sterol from *Cucumis prophetarum*. Pharmaceutical Biology.

[b0090] Alsayari A., Kopel L., Ahmed M.S., Soliman H.S.M., Annadurai S., Halaweish F.T. (2018). Isolation of anticancer constituents from *Cucumis prophetarum* var. *prophetarum* through bioassay-guided fractionation. BMC Complementary and Alternative Medicine.

[b0095] Anjani S.N., Mathur J. (2023). Isolation, purification and characterization of quercetin from *Cucumis sativus* peels; its antimicrobial, antioxidant and cytotoxicity evaluations. 3 Biotech.

[b0100] Araya E.M., Adamu B.A., Periasamy G., Sintayehu B., Gebrelibanos Hiben M. (2019). *In vivo* hepatoprotective and *in vitro* radical scavenging activities of *Cucumis ficifolius* A. Rich root extract. Journal of Ethnopharmacology.

[b0105] Asefa L., Nedi T. (2024). Assessment of the diuretic effect of the leaves of *Cucumis dipsaceus* Ehrenb (Cucurbitaceae) in rats: Using aqueous and 80% methanol extracts. Journal of Experimental Pharmacology.

[b0110] Assefa T., Tesso H., Ramachandran V.P., Guta L., Demissie T.B., Ombito J.O. (2024). *In silico* molecular docking analysis, cytotoxicity, and antibacterial activities of constituents of fruits of *Cucumis dipsaceus*. ACS Omega.

[b0115] Ayele T.T. (2018). A review on traditionally used medicinal plants/herbs for cancer therapy in Ethiopia: Current status, challenge and future perspectives. Organic Chemistry: Current Research.

[b0120] Ayyad S.N., Abdel-Lateff A., Basaif S.A., Shier T. (2011). Cucurbitacins-type triterpene with potent activity on mouse embryonic fibroblast from *Cucumis prophetarum*. Cucurbitaceae. Pharmacognosy Research.

[b0125] Bakam B.Y., Pambe J.C.N., Grey T., Maxeiner S., Rutz J., Njamen D. (2023). *Cucumis sativus* (Cucurbitaceae) seed oil prevents benzo(a)pyrene-induced prostate cancer *in vitro* and *in vivo*. Environmental Toxicology.

[b0130] Barghout N., Djidel S., Bouaziz A., Bentahar A., Dahamna S., Khennouf S. (2024). RP-HPLC analysis of phenolic compounds, quantitative assessment of phytochemicals, antioxidant, analgesic, and anti-inflammatory potential of *Cucumis melo* var. *inodorus* fruit growing in Algeria. Journal of Food Measurement and Characterization.

[b0135] Bharti A., Bora K.S. (2024). Biological evaluation of *Cucumis trigonus* (Roxb.) for memory enhancing activity in scopolamine-induced amnesia in mice. *The*. Natural Products Journal.

[b0140] Bizuneh G.K., Tadege G., Sirak B., Gurmu A.E., Adamu B.A., Tefera A.M. (2023). Antimalarial activity of the 80% methanol extract and solvent fractions of *Cucumis ficifolius* A. rich roots against *Plasmodium berghei* in mice. Heliyon.

[b0145] Bussmann R.W., Glenn A. (2010). Medicinal plants used in Northern Peru for reproductive problems and female health. Journal of Ethnobiology and Ethnomedicine.

[b0150] Bussmann R.W., Paniagua-Zambrana N.Y., Njoroge G.N. (2020).

[b0155] Busuioc A.C., Costea G.V., Botezatu A.V.D., Furdui B., Dinica R.M. (2023). *Cucumis metuliferus* L. fruits extract with antioxidant, anti-inflammatory, and antidiabetic properties as source of ursolic acid. Separations.

[b0160] Buttery R.G., Seifert R.M., Ling L.C., Soderstrom E.L., Ogawa J.M., Turnbaugh J.G. (1982). Additional aroma components of honeydew melon. Journal of Agricultural and Food Chemistry.

[b0165] Chen C., Qiang S.G., Lou L.G., Zhao W.M. (2009). Cucurbitane-type triterpenoids from the stems of *Cucumis melo*. Journal of Natural Products.

[b0170] Chen J.C., Chiu M.H., Nie R.L., Cordell G.A., Qiu S.X. (2005). Cucurbitacins and cucurbitane glycosides: Structures and biological activities. Natural Product Reports.

[b0175] Chen J.F., Zhou X.H. (2011).

[b0180] Chen S.X., Zhang R.R., Hao L.N., Chen W.F., Cheng S.Q. (2015). Profiling of volatile compounds and associated gene expression and enzyme activity during fruit development in two cucumber cultivars. PLoS One.

[b0185] Chen S.Y., Zhou Q.Y., Chen L., Li J.Y., Xie T., Zhang S.H. (2022). Screening and identifying cucurbitacins and cucurbitacin glycosides in *Cucumis sativus* using high-performance liquid chromatography/quadrupole-time-of-flight mass spectrometry combined with in-source fragmentation and alkali adduct ions. Rapid Communications in Mass Spectrometry.

[b0190] Choudhary S.S., Panigrahi P.N., Dhara S.K., Sahoo M., Dan A., Thakur N. (2023). *Cucumis callosus* (Rottl.) Cogn. fruit extract ameliorates calcium oxalate urolithiasis in ethylene glycol induced hyperoxaluric rat model. Heliyon.

[b0195] Da Silva E.M., Dos Santos Magalhães C., Randau K.P. (2023). Descrição botânica, usos etnomedicinais, fitoquímica e atividades farmacológicas de espécies do gênero *Cucumis*: Uma revisão. Diversitas Journal.

[b0200] David A., Vallance D.K. (1955). Bitter principles of Cucurbitaceae. Journal of Pharmacy and Pharmacology.

[b0205] De Marino S., Festa C., Zollo F., Iorizzi M. (2009). Phenolic glycosides from *Cucumis melo* var. *inodorus* seeds. Phytochemistry Letters.

[b0210] Deepika K.A., Prajapati P., Sarita K.S., Aluko R.E. (2023). Pharmacological and therapeutic potential of *Cucumis callosus*: A novel nutritional powerhouse for the management of non-communicable diseases. Plant Foods for Human Nutrition.

[b0215] Demsie D.G., Yimer E.M., Berhe A.H., Altaye B.M., Berhe D.F. (2019). Anti-nociceptive and anti-inflammatory activities of crude root extract and solvent fractions of *Cucumis ficifolius* in mice model. Journal of Pain Research.

[b0220] Derradji F.B., Aoun S. (2022). Evaluation of the anticoagulant activities of *Cucumis melo* rind powder *in vitro*: Preliminary novel findings. Archives of Pharmacy Practice.

[b0225] Du Q.Z., Xiong X.P., Ito Y. (1995). Separation of cucurbitacin B and cucurbitacin E from fruit base of *Cucumis melo* L. by high-speed countercurrent chromatography. *Modern Countercurrent Chromatography*. American Chemical Society.

[b0230] Dunnill P.M., Fowden L. (1965). The amino acids of seeds of the Cucurbitaceae. Phytochemistry.

[b0235] Dwivedi N.K., Dhariwal O.P., Gopala Krishnan S., Bhandari D.C. (2010). Distribution and extent of diversity in *Cucumis* species in the Aravalli ranges of India. Genetic Resources and Crop Evolution.

[b0240] Egoumenides L., Gauthier A., Barial S., Saby M., Orechenkoff C., Simoneau G. (2018). A specific melon concentrate exhibits photoprotective effects from antioxidant activity in healthy adults. Nutrients.

[b0245] Eidi M., Ashjazadeh L. (2023). Anti-urolithiatic effect of *Cucumis melo* L. var *inodorous* in male rats with kidney stones. Urolithiasis.

[b0250] El-Sayed H.M., Rasheed D.M., Mahrous E.A., Abdel-Sattar E. (2025). C_13_-Norisoprenoid megastigmanes: Biosynthesis, classification, natural sources, biological activities, and structure-activity relationship − a comprehensive review. Fitoterapia.

[b0255] El-Sayed H.M., Rasheed D.M., Mahrous E.A., Eltanany B.M., Goda Z.M., Pont L. (2025). Metabolomics analysis of *Cucumis melo* var. *flexuosus* organs in correlation to its anti-inflammatory activity aided by chemometrics. Journal of Pharmaceutical and Biomedical Analysis.

[b0260] Emad A.M., Mahrous E.A., Rasheed D.M., Gomaa F.A.M., Hamdan A.M.E., Selim H.M.R.M. (2024). Wound healing efficacy of Cucurbitaceae seed oils in rats: Comprehensive phytochemical, pharmacological, and histological studies tackling AGE/RAGE and Nrf2/Ho-1 cue. Pharmaceuticals.

[b0265] Enslin P.R. (1954). Bitter principles of the Cucurbitaceae. I.—Observations on the chemistry of cucurbitacin A. Journal of the Science of Food and Agriculture.

[b0270] Ezzat S.M., Raslan M., Salama M.M., Menze E.T., El Hawary S.S. (2019). *In vivo* anti-inflammatory activity and UPLC-MS/MS profiling of the peels and pulps of *Cucumis melo* var. *cantalupensis* and *Cucumis melo* var. *reticulatus*. Journal of Ethnopharmacology.

[b0275] Fawole O.A., Ndhlala A.R., Amoo S.O., Finnie J.F., Van Staden J. (2009). Anti-inflammatory and phytochemical properties of twelve medicinal plants used for treating gastro-intestinal ailments in South Africa. Journal of Ethnopharmacology.

[b0280] Feyisa K., Feyisa W., Girma T., Kemal T. (2022). Traditional medicinal plants used for the treatment of urological and urogenital diseases in Ethiopia: A review. Pharmacognosy Journal.

[b0285] Figueredo E., Cuesta-Herranz J., De-Miguel J., Lázaro M., Sastre J., Quirce S. (2003). Clinical characteristics of melon (*Cucumis melo*) allergy. Annals of Allergy, Asthma & Immunology.

[b0290] Fiume M.M., Bergfeld W.F., Belsito D.V., Hill R.A., Klaassen C.D., Liebler D.C. (2014). Safety assessment of *Cucumis sativus* (cucumber)-derived ingredients as used in cosmetics. International Journal of Toxicology.

[b0295] Forss D.A., Dunstone E.A., Ramshaw E.H., Stark W. (1962). The flavor of cucumbers. Journal of Food Science.

[b0300] Galma W., Endale M., Getaneh E., Eswaramoorthy R., Assefa T., Melaku Y. (2021). Antibacterial and antioxidant activities of extracts and isolated compounds from the roots extract of *Cucumis prophetarum* and in silico study on DNA gyrase and human peroxiredoxin 5. BMC Chemistry.

[b0305] Gao Y., Cai R.L., Xie C., Lin Y.L., Zhang L., Qi Y. (2012). Pharmacological basis for the medicinal use of muskmelon base (*Pedicellus Melo*.) for abdominal distention and constipation. Journal of Ethnopharmacology.

[b0310] Garg V.K., Nes W.R. (1986). Occurrence of Δ^5^-sterols in plants producing predominantly Δ^7^-sterols: Studies on the sterol compositions of six Cucurbitaceae seeds. Phytochemistry.

[b0315] Gawli K., Lakshmidevi N. (2015). Antidiabetic and antioxidant potency evaluation of different fractions obtained from *Cucumis prophetarum* fruit. Pharmaceutical Biology.

[b0320] Gill N.S., Bali M. (2012). Evaluation of antioxidant, antiulcer activity of 9-beta-methyl-19-norlanosta-5-ene type glycosides from *Cucumis sativus* seeds. Research Journal of Medicinal Plant.

[b0325] Gill N.S., Bajwa J., Sharma P., Dhiman K., Sood S., Sharma P.D. (2010). Evaluation of antioxidant and antiulcer activity of traditionally consumed *Cucumis melo* seeds. Journal of Pharmacology and Toxicology.

[b0330] Gómez-García R., Campos D.A., Aguilar C.N., Madureira A.R., Pintado M. (2020). Valorization of melon fruit (*Cucumis melo* L.) by-products: Phytochemical and biofunctional properties with emphasis on recent trends and advances. Trends in Food Science & Technology.

[b0335] Gopalasatheeskumar K., Ariharasivakumar G., Kalaichelvan V.K., Sengottuvel T., Devan V.S., Srividhya V. (2020). Antihyperglycemic and antihyperlipidemic activities of wild musk melon (*Cucumis melo* var. *agrestis*) in streptozotocin-nicotinamide induced diabetic rats. Chinese Herbal Medicines.

[b0340] Gras A., Serrasolses G., Vallès J., Garnatje T. (2019). Traditional knowledge in semi-rural close to industrial areas: Ethnobotanical studies in Western Gironès (Catalonia, Iberian Peninsula). Journal of Ethnobiology and Ethnomedicine.

[b0345] Hemphill D.D., Baker L.R., Sell H.M. (1972). Isolation and identification of the gibberellins of *Cucumis sativus* and *Cucumis melo*. Planta.

[b0350] Hemphill D.D., Baker L.R., Sell H.M. (1973). Isolation of novel conjugated gibberellins from *Cucumis sativus* seed. Canadian Journal of Biochemistry.

[b0355] Hendrayana T., Yoana K., Adnyana I.K., Sukandar E.Y. (2023). Cucumber (*Cucumis sativus* L.) fruit and combination with losartan attenuate the elevation of blood pressure in hypertensive rats induced by angiotensin II. Journal of Pharmacopuncture.

[b0360] Hosoya T., Masuda Y., Ohba S., Kumazawa S. (2026). Component analysis of *Cucumis melo* L. leaves and their antioxidant activity. Natural Product Research.

[b0365] Ibitoye O.B., Uwazie J.N., Ajiboye T.O. (2018). Bioactivity-guided isolation of kaempferol as the antidiabetic principle from *Cucumis sativus* L. fruits. Journal of Food Biochemistry.

[b0370] Ibrahim D.S. (2017). Neuroprotective effect of *Cucumis melo* var. *flexuosus* leaf extract on the brains of rats with streptozotocin-induced diabetes. Metabolic Brain Disease.

[b0375] Ibrahim S.R.M. (2010). New 2-(2-phenylethyl)chromone derivatives from the seeds of *Cucumis melo* L var. *reticulatus*. Natural Product Communications.

[b0380] Ibrahim S.R.M. (2014). New chromone and triglyceride from *Cucumis melo* seeds. Natural Product Communications.

[b0385] Ibrahim S.R.M., Mohamed G.A. (2015). Cucumin S, a new phenylethyl chromone from *Cucumis melo* var. *reticulatus* seeds. Revista Brasileira de Farmacognosia.

[b0390] Ibrahim S.R.M., Khedr A.I.M., Mohamed G.A., Zayed M.F., El-Kholy A.A.S., Al Haidari R.A. (2019). Cucumol B, a new triterpene benzoate from *Cucumis melo* seeds with cytotoxic effect toward ovarian and human breast adenocarcinoma. Journal of Asian Natural Products Research.

[b0395] Ibrahim S., Al Haidari R., Mohamed G., Elkhayat E., Moustafa M. (2016). Cucumol A: A cytotoxic triterpenoid from *Cucumis melo* seeds. Revista Brasileira de Farmacognosia.

[b0400] Ibrahim T.A., El-Hefnawy H.M., El-Hela A.A. (2010). Antioxidant potential and phenolic acid content of certain cucurbitaceous plants cultivated in Egypt. Natural Product Research.

[b0405] Idemudia O.U., Enogieru A.B. (2024). Phytochemical and pharmacological activities of *Cucumis sativus*: An updated review. Tropical Journal of Natural Product Research.

[b0410] Insanu M., Rizaldy D., Silviani V., Fidrianny I. (2022). Chemical compounds and pharmacological activities of *Cucumis* genus. Biointerface Research in Applied Chemistry.

[b0415] Itoh T., Shigemoto T., Shimizu N., Tamura T., Matsumoto T. (1982). Triterpene alcohols in the seeds of two *Cucumis* species of Cucurbitaceae. Phytochemistry.

[b0420] Jevtić B., Djedović N., Stanisavljević S., Gašić U., Mišić D., Despotović J. (2017). Anti-encephalitogenic effects of cucumber leaf extract. Journal of Functional Foods.

[b0425] Ji L., Gao W., Wei J., Pu L., Yang J., Guo C. (2015). *In vivo* antioxidant properties of lotus root and cucumber: A pilot comparative study in aged subjects. The Journal of Nutrition, Health and Aging.

[b0430] Jordán M.J., Shaw P.E., Goodner K.L. (2001). Volatile components in aqueous essence and fresh fruit of *Cucumis melo* cv. Athena (muskmelon) by GC-MS and GC-O. Journal of Agricultural and Food Chemistry.

[b0435] Kamimura M., Sasaki A., Otani Y., Nakamura Y., Nakamura T., Kuramochi K. (2023). Methylthioacetic acid, a derivative of aroma compounds from *Cucumis melo* var. *conomon* dose-dependently triggers differentiation and apoptosis of RCM-1 human colorectal cancer cells. The Journal of Toxicological Sciences.

[b0440] Kasote D.M., Jagtap S.D., Thapa D., Khyade M.S., Russell W.R. (2017). Herbal remedies for urinary stones used in India and China: A review. Journal of Ethnopharmacology.

[b0445] Kavishankar G.B., Lakshmidevi N. (2014). Anti-diabetic effect of a novel *N*-trisaccharide isolated from *Cucumis prophetarum* on streptozotocin−nicotinamide induced type 2 diabetic rats. Phytomedicine.

[b0450] Kavishankar G.B., Moree S.S., Lakshmidevi N. (2014). Hepatoprotective and antioxidant activity of *N*-trisaccharide in different experimental rats. Phytomedicine.

[b0455] Kemp T.R. (1977). A C_15_ aldehyde from *Cucumis sativus*. Phytochemistry.

[b0460] Kemp T.R., Stoltz L.P., Knavel D.E. (1972). Volatile components of muskmelon fruit. Journal of Agricultural and Food Chemistry.

[b0465] Khan A., Mishra A., Hasan S.M., Usmani A., Ubaid M., Khan N. (2022). Biological and medicinal application of *Cucumis sativus* Linn. − Review of current status with future possibilities. Journal of Complementary and Integrative Medicine.

[b0470] Khanal M., Lekhak H.D., Kunwar R.M., Bussmann R.W., Paniagua-Zambrana N.Y. (2021). *hardwickii* (Royle) Alef Cucurbitaceae. *Ethnobotany of the Himalayas*.

[b0475] Khetkam P., Xie X.N., Kisugi T., Kim H.I., Yoneyama K., Uchida K. (2014). 7*α*- and 7*β*-Hydroxyorobanchyl acetate as germination stimulants for root parasitic weeds produced by cucumber. Journal of Pesticide Science.

[b0480] Kimathi P.K., Maitho T., Mbaria J., Moriasi G. (2022). Antidiarrheal, antimicrobial, and toxic effects of the aqueous and methanolic leaf and fruit extracts of *Cucumis dipsaceus* (Ehrenb. Ex Spach.). Journal of Herbmed Pharmacology.

[b0485] Kintia P.K., Wojciechowski Z.A. (1975). Pentacyclic triterpenes and typical sterol precursors in *Cucumis sativus* seedlings. Phytochemistry.

[b0490] Knights B.A., Smith A.R. (1977). Sterols of male and female flowers of *Cucumis sativus*. Planta.

[b0495] Kothari S., Saravana M., Muthusamy S., Mozingo A., Soni M. (2018). Safety assessment of a standardized cucumber extract (Q-Actin™): Oral repeat-dose toxicity and mutagenicity studies. Toxicology Reports.

[b0500] Krauze-Baranowska M., Cisowski W. (2001). Flavonoids from some species of the genus *Cucumis*. Biochemical Systematics and Ecology.

[b0505] Kumar V., Shivam S.N., Chandra P. (2020). A review on morphology, traditional uses and pharmacological activities and phytochemicals of *Cucumis trigonus*. International Journal of Pharmaceutical Sciences Review and Research.

[b0510] Lal D., Lata K. (1980). Plants used by the Bhat community for regulating fertility. Economic Botany.

[b0515] Lata S., Mittal S.K. (2017). Identification of isolated flavonoid glycoside from methanolic extract of *Cucumis dipsaceus* Ehrenb. (fruit). International Journal of Pharmacognosy and Phytochemical Research.

[b0520] Lata, S., & Mittal, S. K. (2017b). *In vitro* and *in vivo* hepatoprotective activity of flavonoids rich extracts on *Cucumis dipsaceus* Ehrenb. (fruit). *International Journal of Pharmacology*, *13*(6), 563−572.

[b0525] Lija M., Beevy S.S. (2021). A review on the diversity of melon. Plant Science Today.

[b0530] Liu Z.G., Wang R., Zhang C.H., Guo S.S., Chen P.J. (2019). A case of vitiligo cured with cucumber and sulfur. Phytotherapy Research.

[b0535] Ma Q.G., Wei R.R. (2021). A new anthraquinone-aurone adduct with hepatoprotective activity from the fruits of *Cucumis bisexualis*. Chemistry of Natural Compounds.

[b0540] Ma Q.G., Wei R.R. (2021). Isolation and characterization of hepatoprotective anthraquinone derivatives from *Cucumis bisexualis*. Chemistry of Natural Compounds.

[b0545] Ma Q.G., Wei R.R. (2023). Isolation and characterization of auronolignan derivatives with hepatoprotective activities from *Cucumis bisexualis*. Chemistry of Natural Compounds.

[b0550] Ma Q.G., Liu W.M., Wei R.R. (2024). Isolation and characterization of flavonolignan from *Cucumis bisexualis* and their hepatoprotective activities. Chemistry of Natural Compounds.

[b0555] Ma Q.G., Liu W.M., Sang Z.P., Wei R.R. (2025). Hepatoprotective biphenyl derivatives from *Cucumis bisexualis*. Chemistry of Natural Compounds.

[b0560] Ma Q.G., Wei R.R., Sang Z.P. (2020). Bioactivity-guided isolation of aurone derivatives with hepatoprotective activities from the fruits of *Cucumis bisexualis*. Zeitschrift Fur Naturforschung - C Journal of Biosciences.

[b0565] Ma Q.G., Wei R.R., Sang Z.P. (2020). Hepatoprotective homoisoflavonoids from the fruits of *Cucumis bisexualis*. Journal of Food Biochemistry.

[b0570] Ma Q.G., Wei R.R., Sang Z.P. (2020). Structural characterization and hepatoprotective activity of naphthoquinone from *Cucumis bisexualis*. Natural Product Communications.

[b0575] Ma Q.G., Wei R.R., Sang Z.P., Dong J.H. (2021). Structurally diverse coumarin-homoisoflavonoid derivatives with hepatoprotective activities from the fruits of *Cucumis bisexualis*. Fitoterapia.

[b0580] Ma Q.G., Wei R.R., Yang M., Huang X.Y., Wang F., Sang Z.P. (2018). Molecular characterization and bioactivity of coumarin derivatives from the fruits of *Cucumis bisexualis*. Journal of Agricultural and Food Chemistry.

[b0585] Mahjour M., Banihashemi M., Rakhshandeh H., Vakili V., Khoushabi A., Kakhki M.T. (2020). A triple-blind, randomized trial of a traditional compound as compared to 4% hydroquinone in melasma. Journal of Herbal Medicine.

[b0590] Maja D., Mavengahama S., Mashilo J. (2022). Cucurbitacin biosynthesis in cucurbit crops, their pharmaceutical value and agricultural application for management of biotic and abiotic stress: A review. South African Journal of Botany.

[b0595] Malik Z.A., Bhat J.A., Ballabha R., Bussmann R.W., Bhatt A.B. (2015). Ethnomedicinal plants traditionally used in health care practices by inhabitants of Western Himalaya. Journal of Ethnopharmacology.

[b0600] Manjunathagowda D.C., Pitchaimuthu M., Hiremata V., Sathisha G.C., Soni S., Dhananjaya M.V. (2023). Horny gourd (*Cucumis metuliferus* L.): A hidden vegetable boon for human nutrition. Genetic Resources and Crop Evolution.

[b0605] Marisol M.M., Celeste T.M., Laura M.M., Fernando E.G., José P.C., Alejandro Z. (2019). Effect of *Cucumis sativus* on dysfunctional 3T3-L1 adipocytes. Scientific Reports.

[b0610] Matsumoto T., Shigemoto T., Itoh T. (1983). (22*E*,24*S*)-5*α*-Ergosta-7,22-dien-3*β*-ol from the seeds of *Cucumis sativus*. Phytochemistry.

[b0615] Matsumoto T., Shigemoto T., Itoh T. (1983). Occurrence of 24-ethyl- Δ^5^- and 24-ethyl- Δ^7^-sterols as C-24 epimeric mixtures in seeds of *Cucumis sativus*. Phytochemistry.

[b0620] McNally D.J., Wurms K.V., Labbé C., Bélanger R.R. (2003). Synthesis of *C*-glycosyl flavonoid phytoalexins as a site-specific response to fungal penetration in cucumber. Physiological and Molecular Plant Pathology.

[b0625] Meragiaw M., Asfaw Z., Argaw M. (2016). The status of ethnobotanical knowledge of medicinal plants and the impacts of resettlement in Delanta, northwestern Wello, northern Ethiopia. Evidence-Based Complementary and Alternative Medicine.

[b0630] Milder I.E.J., Arts I.C.W., van de Putte B., Venema D.P., Hollman P.C.H. (2005). Lignan contents of dutch plant foods: A database including lariciresinol, pinoresinol, secoisolariciresinol and matairesinol. British Journal of Nutrition.

[b0635] Miró M. (1995). Cucurbitacins and their pharmacological effects. Phytotherapy Research.

[b0640] Moing A., Allwood J.W., Aharoni A., Baker J., Beale M.H., Ben-Dor S. (2020). Comparative metabolomics and molecular phylogenetics of melon (*Cucumis melo*, Cucurbitaceae) biodiversity. Metabolites.

[b0645] Mukherjee P.K., Nema N.K., Maity N., Sarkar B.K. (2013). Phytochemical and therapeutic potential of cucumber. Fitoterapia.

[b0650] Mukherjee P.K., Singha S., Kar A., Chanda J., Banerjee S., Dasgupta B. (2022). Therapeutic importance of Cucurbitaceae: A medicinally important family. Journal of Ethnopharmacology.

[b0655] Mukungu N., Abuga K., Okalebo F., Ingwela R., Mwangi J. (2016). Medicinal plants used for management of malaria among the Luhya community of Kakamega East sub-County, Kenya. Journal of Ethnopharmacology.

[b0660] Muth C.M., Glenz Y., Klaus M., Radermacher P., Speit G., Leverve X. (2004). Influence of an orally effective SOD on hyperbaric oxygen-related cell damage. Free Radical Research.

[b0665] Naik V.R., Agshikar N.V., Abraham G.J. (1980). Analgesic and anti-inflammatory activity in alcoholic extracts of *Cucumis trigonus* Roxburghii. A preliminary communication. Pharmacology.

[b0670] Naik V.R., Agshikar N.V., Abraham G.J.S. (1981). *Cucumis trigonus* Roxb II. Diuretic activity. Journal of Ethnopharmacology.

[b0675] Nash R.J., Azantsa B.K., Sharp H., Shanmugham V. (2018). Effectiveness of *Cucumis sativus* extract versus glucosamine-chondroitin in the management of moderate osteoarthritis: A randomized controlled trial. Clinical Interventions in Aging.

[b0680] Nash R.J., Bartholomew B., Penkova Y.B., Rotondo D., Yamasaka F., Stafford G.P. (2020). Iminosugar idoBR1 isolated from cucumber *Cucumis sativus* reduces inflammatory activity. ACS Omega.

[b0685] Negara C.K., Erna E.N., Anna A.N. (2018). The effect of cucumber juice (*Cucumis sativus*) toward hypertension of elderly at Tresna Werdha Budi Sejahtera Social Institution of Banjarbaru South Borneo 2017. Indonesian Journal of Nursing Practice.

[b0690] Nigussie G., Ashenef S. (2020). Isolation, characterization, structural elucidation and anti-bacterial activities of roots extracts of *Cucumis ficifolius*. Research Square.

[b0695] Njoroge G.N., Newton L.E. (1994). Edible and poisonous species of Cucurbitaceae in the central Highlands of Kenya. Journal of East African Natural History.

[b0700] Oboh G., Ademiluyi A.O., Ogunsuyi O.B., Oyeleye S.I., Dada A.F., Boligon A.A. (2017). Cabbage and cucumber extracts exhibited anticholinesterase, antimonoamine oxidase and antioxidant properties. Journal of Food Biochemistry.

[b0705] Ofoego U.C., Nweke E.O., Nzube O.M., Campus N., State A., State A. (2019). Ameliorative effect of ethanolic extract of *Cucumis sativus* (cucumber) pulp on alloxan-induced kidney toxicity in male adult Wistar rats. Journal of Natural Sciences Research.

[b0710] Olarewaju O.O., Fajinmi O.O., Arthur G.D., Coopoosamy R.M., Naidoo K.K. (2021). Food and medicinal relevance of Cucurbitaceae species in Eastern and Southern Africa. Bulletin of the National Research Centre.

[b0715] Olennikov D.N. (2023). Separation, characterization and mammal pancreatic lipase inhibitory potential of cucumber flower flavonoids. Separations.

[b0720] Olennikov D.N., Kashchenko N.I. (2023). Acylated flavonoids from *Cucumis sativus* inhibit the activity of human pancreatic lipase. Applied Biochemistry and Microbiology.

[b0725] Olennikov D.N., Kashchenko N.I. (2023). Green waste from cucumber (*Cucumis sativus* L.) cultivation as a source of bioactive flavonoids with hypolipidemic potential. Agronomy.

[b0730] Olennikov D.N., Kashchenko N.I. (2023). New flavonoids from *Cucumis sativus*. Chemistry of Natural Compounds.

[b0735] Olennikov D.N., Kashchenko N.I. (2024). New acylated *C*,*O*-glycosylflavones from *Cucumis sativus*. Chemistry of Natural Compounds.

[b0740] Olennikov D.N., Kashchenko N.I. (2024). Minor *C*,*O*-glycosylflavones from *Cucumis sativus*. Chemistry of Natural Compounds.

[b0745] Olisova O.Y., Snarskaya E.S., Gladko V.V., Burova E.P. (2018). Russian traditional medicine in dermatology. Clinics in Dermatology.

[b0750] Omokhua-Uyi A.G., Van Staden J. (2020). Phytomedicinal relevance of South African Cucurbitaceae species and their safety assessment: A review. Journal of Ethnopharmacology.

[b0755] Panda S.P., Jena B.R., Kalyani G., Panigrahy U.P. (2018). Overexpressed CYP450 mediated apoptosis evaluates cytotoxicity and teratotoxicity of *Cucumis callosus*. Oriental Pharmacy and Experimental Medicine.

[b0760] Parmar H.S., Kar A. (2009). Protective role of *Mangifera indica*, *Cucumis melo* and *Citrullus vulgaris* peel extracts in chemically induced hypothyroidism. Chemico-Biological Interactions.

[b0765] Parvinroo S., Naghibi F., Zahediasl S., Kamalinejad M., Sabetkasaei M. (2014). The effects of seeds with hot and cold temperaments on serum thyroid hormones, corticosterone and urine vanillylmandelic acid concentrations of healthy rats. Journal of Ethnopharmacology.

[b0770] Paul B.M., Jagadeesan G., Kannan G., Jegan Raj F., Annadurai Y., Piramanayagam S. (2024). Exploring the hypoglycaemic efficacy of bio-accessed antioxidative polyphenolics in thermally processed *Cucumis dipsaceus* fruits − an *in vitro* and *in silico* study. Food Chemistry.

[b0775] Pérez-Piñero S., Muñoz-Carrillo J.C., Victoria-Montesinos D., García-Muñoz A.M., Ávila-Gandía V., López-Román F.J. (2023). Effectiveness of a cucumber extract supplement on articular pain in patients with knee osteoarthritis: A randomized double-blind controlled clinical trial. Applied Sciences.

[b0780] Piao X.M., Gao F., Zhu J.X., Wang L.J., Zhao X., Li X. (2018). Cucurbitacin B inhibits tumor angiogenesis by triggering the mitochondrial signaling pathway in endothelial cells. International Journal of Molecular Medicine.

[b0785] Qing Z.X., Shi Y., Han L.D., Li P.K., Zha Z.O., Liu C. (2022). Identification of seven undescribed cucurbitacins in *Cucumis sativus* (cucumber) and their cytotoxic activity. Phytochemistry.

[b0790] Raja Soh R.S.S., Hapidin H., Kasiram M.Z. (2024). A scoping review on *Cucumis melo* and its anti-cancer properties. The Malaysian Journal of Medical Sciences.

[b0795] Rashid R., Rather S.A., Bhat S.A. (2024). Safety and efficacy of decoction obtained from *Asparagus officinalis* and *Cucumis melo* in the management of nephrolithiasis: Single blind, randomized, active controlled parallel arm study. International Journal of Community Medicine and Public Health.

[b0800] Rasouli H., Parvaneh S., Mahnam A., Rastegari-Pouyani M., Hoseinkhani Z., Mansouri K. (2017). Anti-angiogenic potential of trypsin inhibitor purified from *Cucumis melo* seeds: Homology modeling and molecular docking perspective. International Journal of Biological Macromolecules.

[b0805] Rehm S., Enslin P.R., Meeuse A.D.J., Wessels J.H. (1957). Bitter principles of the Cucurbitaceae. VII.—The distribution of bitter principles in this plant family. Journal of the Science of Food and Agriculture.

[b0810] Ribeiro, J. E. d. S., Nunes, E. N., Souza, R. S., da Cruz, D. D., & de Lucena, R. F. P. (2023). *Cucumis anguria* L. Cucurbitaceae. In *Ethnobotany of the Mountain Regions of Brazil*. Springer International Publishing, pp 329−335.

[b0815] Rimington C. (1933). The toxic principles of *Cucumis africanus* L. f., *Cucumis myriocarpus* (Naud.) emend. and of a new unnamed *Cucumis* species. South African Journal of Science.

[b0820] Rimington C. (1935). Isolation of the toxic principles of *Cucumis africanus* L. f. *Cucumis myriocarpus* Naud. emend. Schweikerdt and of *Cucumis leptodermis* Schweikerdt sp. nov. their characterization as trilactones belonging to the “Bitter Principle” class. Onderstepoort Journal of Veterinary Science.

[b0825] Roman-Ramos R., Flores-Saenz J.L., Alarcon-Aguilar F.J. (1995). Anti-hyperglycemic effect of some edible plants. Journal of Ethnopharmacology.

[b0830] Saby M., Gauthier A., Barial S., Egoumenides L., Jover B. (2020). Supplementation with a bioactive melon concentrate in humans and animals: Prevention of oxidative damages and fatigue in the context of a moderate or eccentric physical activity. International Journal of Environmental Research and Public Health.

[b0835] Salahuddin M., Jalalpure S.S. (2010). Antidiabetic activity of aqueous fruit extract of *Cucumis trigonus* Roxb. in streptozotocin-induced-diabetic rats. Journal of Ethnopharmacology.

[b0840] Salama A.M., Solano P.P. (2001). Antinociceptive, antimicrobial, antitumor activity of *Cucumis anguria*. Revista Colombiana de Ciencias Químico-Farmacéuticas.

[b0845] Sasaki A., Nakamura Y., Kobayashi Y., Aoi W., Nakamura T., Shirota K. (2020). Contribution of Katsura-uri (Japan’s heirloom pickling melon, *Cucumis melo* var. *conomon*) at the completely ripe stage to diabetes control. Journal of Nutritional Science and Vitaminology.

[b0850] Satoh J., Koshino H., Sekino K., Ito S., Katsuta R., Takeda K. (2016). *Cucumis sativus* secretes 4'-ketoriboflavin under iron-deficient conditions. Bioscience, Biotechnology, and Biochemistry.

[b0855] Semenya S.S., Potgieter M.J., Erasmus L.J.C. (2013). Exotic and indigenous problem plants species used, by the Bapedi, to treat sexually transmitted infections in Limpopo Province. South Africa. African Health Sciences.

[b0860] Šeregelj V., Šovljanski O., Tumbas Šaponjac V., Vulić J., Ćetković G., Markov S. (2022). Horned melon (*Cucumis metuliferus* E. Meyer ex. Naudin)—Current knowledge on its phytochemicals, biological benefits, and potential applications. Processes.

[b0865] Shabab S., Gholamnezhad Z., Mahmoudabady M. (2021). Protective effects of medicinal plant against diabetes induced cardiac disorder: A review. Journal of Ethnopharmacology.

[b0870] Shaik R.S., Burrows G.E., Urwin N.A.R., Gopurenko D., Lepschi B.J., Weston L.A. (2017). The biology and management of prickly paddy melon (*Cucumis myriocarpus* L.), an important summer annual weed in Australia. Crop Protection.

[b0875] Shang Y., Ma Y.S., Zhou Y., Zhang H.M., Duan L.X., Chen H.M. (2014). Biosynthesis, regulation, and domestication of bitterness in cucumber. Science.

[b0880] Shu C.K., Chung H.L., Lawrence B.M. (1995). Volatile components of pocket melon (*Cucumis melo* L. ssp. *dudaim* Naud.). Journal of Essential Oil Research.

[b0885] Silva M.A., Albuquerque T.G., Alves R.C., Oliveira M.B.P.P., Costa H.S. (2020). Melon (*Cucumis melo* L.) by-products: Potential food ingredients for novel functional foods?. Trends in Food Science & Technology.

[b0890] Singh V., Kaur R., Devashree Y., Kaur D., Gupta S. (2022). *In vitro* antimicrobial activity of *Cucumis* and *Momordica* L. against human pathogens. Doklady Biological Sciences.

[b0895] Sitoe E., Van Wyk B.E. (2024). An inventory and analysis of the medicinal plants of Mozambique. Journal of Ethnopharmacology.

[b0900] Smith V.A., Sponsel V.M., Knatt C., Gaskin P., MacMillan J. (1991). Immunochromatographic purification of gibberellins from vegetative tissues of *Cucumis sativus* L: Separation and identification of 13-hydroxy and 13-deoxy gibberellins. Planta.

[b0905] Soltani R., Hashemi M., Farazmand A., Asghari G., Heshmat-Ghahdarijani K., Kharazmkia A. (2017). Evaluation of the effects of *Cucumis sativus* seed extract on serum lipids in adult hyperlipidemic patients: A randomized double-blind placebo-controlled clinical trial. Journal of Food Science.

[b0910] Srivastava A.K., Mukerjee A., Tripathi A. (2020). Antidiabetic and antihyperlipidemic activities of *Cucumis melo* var. *momordica* fruit extract on experimental animals. Future Journal of Pharmaceutical Sciences.

[b0915] Stafford G.I., Pedersen M.E., van Staden J., Jäger A.K. (2008). Review on plants with CNS-effects used in traditional south african medicine against mental diseases. Journal of Ethnopharmacology.

[b0920] Subandi W.M., Sudarmo T.P.B., Suarsini E. (2018). Saponin isolates from cucumber (*Cucumis sativus* L.) fruit mesocarp and their activity as pancreatic lipase inhibitor. American Institute of Physics Conference Proceedings.

[b0925] Sundari T., Kavitha R. (2024). *In vitro* assessing of *Cucumis pubescens* Willd. fruit extract for phytochemical, antibacterial, antioxidants and toxicity assays. Journal of the Indian Chemical Society.

[b0930] Tamiru E., Temesegen A., Demise D. (2019). Phytochemical investigation on the root extraction of *Cucumis prophetarum* L. Chemistry Africa.

[b0935] Tang J., Meng X.J., Liu H., Zhao J.L., Zhou L.G., Qiu M.H. (2010). Antimicrobial activity of sphingolipids isolated from the stems of cucumber (*Cucumis sativus* L.). Molecules.

[b0940] Teklehaymanot T., Giday M. (2007). Ethnobotanical study of medicinal plants used by people in Zegie Peninsula, Northwestern Ethiopia. Journal of Ethnobiology and Ethnomedicine.

[b0945] Teklehaymanot T., Giday M., Medhin G., Mekonnen Y. (2007). Knowledge and use of medicinal plants by people around Debre Libanos monastery in Ethiopia. Journal of Ethnopharmacology.

[b0950] Thanthong S., Nanthong R., Kongwattanakul S., Laebua K., Trirussapanich P., Pitiporn S. (2020). Prophylaxis of radiation-induced dermatitis in patients with breast cancer using herbal creams: A prospective randomized controlled trial. Integrative Cancer Therapies.

[b0955] Trejo-Moreno C., Méndez-Martínez M., Zamilpa A., Jiménez-Ferrer E., Perez-Garcia M.D., Medina-Campos O.N. (2018). *Cucumis sativus* aqueous fraction inhibits angiotensin II-induced inflammation and oxidative stress *in vitro*. Nutrients.

[b0960] Tuseef Sayyar H., Afroz S., Khan A. (2023). Neuropharmacological evaluation of different species of Curcubitaceae seeds extract in experimental animals. Pakistan Journal of Pharmaceutical Sciences.

[b0965] Ul Haq F., Ali A., Khan M.N., Shah S.M.Z., Kandel R.C., Aziz N. (2019). Metabolite profiling and quantitation of cucurbitacins in Cucurbitaceae plants by liquid chromatography coupled to tandem mass spectrometry. Scientific Reports.

[b0970] Ulubelen A., Baytop T., Çubukcu B. (1976). Identification of steroidal and triterpenic compounds of *Cucumis trigonus*. Planta Medica.

[b0975] Umair M., Altaf M., Bussmann R.W., Abbasi A.M. (2019). Ethnomedicinal uses of the local flora in Chenab riverine area, Punjab province Pakistan. Journal of Ethnobiology and Ethnomedicine.

[b0980] Vouldoukis I., Lacan D., Kamate C., Coste P., Calenda A., Mazier D. (2004). Antioxidant and anti-inflammatory properties of a *Cucumis melo* LC. extract rich in superoxide dismutase activity. Journal of Ethnopharmacology.

[b0985] Wahid M., Saqib F., Abbas G., Shah S., Alshammari A., Albekairi T.H. (2024). Cardioprotective and hypotensive mechanistic insights of hydroethanolic extract of *Cucumis melo* L. kernels in isoprenaline-induced cardiotoxicity based on metabolomics and *in silico* electrophysiological models. Frontiers in Pharmacology.

[b0990] Wahid M., Saqib F., Chicea L., Ahmedah H.T., Sajer B.H., Marc (Vlaic) R.A. (2022). Metabolomics analysis delineates the therapeutic effects of hydroethanolic extract of *Cucumis sativus* L. seeds on hypertension and isoproterenol-induced myocardial infarction. Biomedicine & Pharmacotherapy.

[b0995] Wright C.I., Van-Buren L., Kroner C.I., Koning M.M.G. (2007). Herbal medicines as diuretics: A review of the scientific evidence. Journal of Ethnopharmacology.

[b1000] Xie Y., Zhu G.X., Yi J.L., Ji Y.Y., Xia Y., Zheng Y. (2022). A new product of multi-plant extracts improved skin photoaging: An oral intake *in vivo* study. Journal of Cosmetic Dermatology.

[b1005] Yabumoto K., Jennings W.G. (1977). Volatile constituents of cantaloupe, *Cucumis melo*, and their biogenesis. Journal of Food Science.

[b1010] Yadav J.P., Grishina M., Shahbaaz M., Mukerjee A., Singh S.K., Pathak P. (2022). *Cucumis melo* var. *momordica* as a potent antidiabetic, antioxidant and possible anticovid alternative: Investigation through experimental and computational methods. Chemistry & Biodiversity.

[b1015] Yang S.L., Walters T.W. (1992). Ethnobotany and the economic role of the Cucurbitaceae of China. Economic Botany.

[b1020] Yoon J.Y., Chung I.M., Thiruvengadam M. (2015). Evaluation of phenolic compounds, antioxidant and antimicrobial activities from transgenic hairy root cultures of gherkin (*Cucumis anguria* L.). South African Journal of Botany.

[b1025] Yuan R.Q., Qian L., Yun W.J., Cui X.H., Lv G.X., Tang W.Q. (2019). Cucurbitacins extracted from *Cucumis melo* L. (CuEC) exert a hypotensive effect via regulating vascular tone. Hypertension Research.

[b1030] Zhou X.J., Li X.S., Shen Y., Pei G., Wang J.F., Cheng Y.X. (2012). Steroids and triterpenoids from *Cucumis sativus* roots. Chemistry of Natural Compounds.

[b1035] Zhu M.Q., Huang R.M., Wen P., Song Y., He B.L., Tan J.L. (2021). Structural characterization and immunological activity of pectin polysaccharide from kiwano (*Cucumis metuliferus*) peels. Carbohydrate Polymers.

