# Supplementary material for: Genus Cucumis: Traditional uses, phytochemistry, pharmacology, clinical application, and toxicology
Source: Chin Herb Med. 2026 Feb 12;18(2):343–76. doi: 10.1016/j.chmed.2026.02.005 (PMC13069671; doi:10.1016/j.chmed.2026.02.005)
Supplement: Supplementary Data 1 [file mmc1.docx]

**Supplementary materials**

**Table S1** Secondary metabolites reported in genus *Cucumis*, comprising 428 compounds: steroids (**1**−**73**), triterpenoids (**74**−**175**), flavonoids (**176**−**265**), coumarins and other phenolics (**266**−**359**), and other compounds (**360**−**428**), excluding volatile oils.

| No. | Secondary metabolites | Species | Organs | References |
| --- | --- | --- | --- | --- |
| Steroids (73 compounds) | | | | |
| **1** | 24-Methylcholestanol; [24*ξ*-Methyl-5*α*-cholestan-3*β*-ol] | *C. sativus* | Seeds; Aerial parts | Akihisa, Thakur, Rosenstein, & Matsumoto, 1986 |
| **2** | 3-*O*-(6′-*O*-Palmitoyl-*β-D*-glucosyl)-stigmastanol | *C. sativus* | Fruits | Tunmann & Frank, 1972 |
| **3** | 3-*O*-(6′-*O*-Stearyl-*β-D*-glucosyl)-stigmastanol | *C. sativus* | Fruits | Tunmann & Frank, 1972 |
| **4** | 24*ξ*-Ethyl-cholestanol | *C. sativus* | Seeds; Aerial parts | Akihisa, Thakur, Rosenstein, & Matsumoto, 1986 |
| **5** | 24*ξ*-Ethyl-dehydrocholestanol | *C. sativus* | Seeds | Akihisa, Thakur, Rosenstein, & Matsumoto, 1986 |
| **6** | Desmosterol; [Cholesta-5,24(25)-dien-3*β*-ol]; [24-Dehydrocholesterol] | *C. sativus* | Seeds; Aerial parts | Akihisa (ne Itoh), Ghosh, Thakur, Rosentein, & Matsumoto, 1986; Akihisa, Thakur, Rosenstein, & Matsumoto, 1986 |
|  |  | *C. melo* | Seeds | Akihisa (ne Itoh), Ghosh, Thakur, Rosentein, & Matsumoto, 1986 |
| **7** | Cholesterol; [Cholest-5-en-3*β*-ol] | *C. sativus* | Seeds; Aerial parts; Flowers | Akihisa (ne Itoh), Ghosh, Thakur, Rosentein, & Matsumoto, 1986; Akihisa, Thakur, Rosenstein, & Matsumoto, 1986; Knights & Smith, 1977 |
| **8** | 24*α*-Methyl-22-dehydrocholesterol | *C. sativus* | Seeds; Aerial parts | Akihisa (ne Itoh), Ghosh, Thakur, Rosentein, & Matsumoto, 1986; Akihisa, Thakur, Rosenstein, & Matsumoto, 1986 |
| **9** | Brassicasterol; [24*β*-Methyl-22-dehydrocholesterol] | *C. sativus* | Seeds; Aerial parts | Akihisa (ne Itoh), Ghosh, Thakur, Rosentein, & Matsumoto, 1986; Akihisa, Thakur, Rosenstein, & Matsumoto, 1986 |
| **10** | Campesterol; [24*α*-Methyl-5*α*-cholest-5-en-3*β*-ol] | *C. sativus* | Seeds; Aerial parts | Akjhisa et al., 1987; Akihisa, Thakur, Rosenstein, & Matsumoto, 1986; Garg & Nes, 1986 |
|  |  | *C. melo* | Seeds | Garg & Nes, 1986 |
| **11** | Campesterol 3-*O*-*β-D*-glucopyranoside | *C. callosus* | Fruits | Abd El-Fattah, Zaghloul, Halim, & Waight, 1989 |
| **12** | 24*β*-Methylcholesterol; [24*β*-Methylcholest-5-en-3*β*-ol]; [24*β*-Methyl-5*α*-cholest-5-en-3*β*-ol]; [22-Dihydrobrassicasterol] | *C. sativus* | Seeds; Aerial parts | Akjhisa et al., 1987; Akihisa (ne Itoh), Ghosh, Thakur, Rosentein, & Matsumoto, 1986; Akihisa, Thakur, Rosenstein, & Matsumoto, 1986; Garg & Nes, 1986 |
|  |  | *C. melo* | Seeds | Akihisa (ne Itoh), Ghosh, Thakur, Rosentein, & Matsumoto, 1986; Garg & Nes, 1986 |
| **13** | 24*ξ*-Methylcholesterol | *C. sativus* | Flowers | Knights & Smith, 1977 |
| **14** | 24-Methylenecholesterol; [24-Methylene-25-methyl-5*α*-cholest-5-en-3*β*-ol]; [24-Methylenecholest-5-en-3*β*-ol] | *C. sativus* | Seeds; Aerial parts | Akihisa (ne Itoh), Ghosh, Thakur, Rosentein, & Matsumoto, 1986; Akihisa, Thakur, Rosenstein, & Matsumoto, 1986 |
|  |  | *C. melo* | Seeds | Akjhisa et al., 1987; Akihisa (ne Itoh), Ghosh, Thakur, Rosentein, & Matsumoto, 1986 |
| **15** | Codisterol | *C. sativus*; *C. melo* | Seeds | Garg & Nes, 1986 |
| **16** | Poriferasterol; [24*β*-Ethyl-5*α*-cholesta-5,22*E*-dien-3*β*-ol] | *C. sativus* | Seeds; Aerial parts | Akjhisa et al., 1987; Akihisa (ne Itoh), Ghosh, Thakur, Rosentein, & Matsumoto, 1986 |
|  |  | *C. melo* | Seeds | Akihisa (ne Itoh), Ghosh, Thakur, Rosentein, & Matsumoto, 1986 |
| **17** | Stigmasterol; [24*α*-Ethyl-5*α*-cholesta-5,22*E*-dien-3*β*-ol]; [24*α*-Ethyl-22-dehydrocholesterol] | *C. sativus* | Seeds; Aerial parts | Akjhisa et al., 1987; Akihisa (ne Itoh), Ghosh, Thakur, Rosentein, & Matsumoto, 1986; Akihisa, Thakur, Rosenstein, & Matsumoto, 1986; Garg & Nes, 1986; Mashchenko, Kintya, & Lazur’evskii, 1975 |
|  |  | *C. melo* | Seeds | Akihisa (ne Itoh), Ghosh, Thakur, Rosentein, & Matsumoto, 1986; Garg & Nes, 1986; Mashchenko, Kintya, & Lazur’evskii, 1975 |
| **18** | Stigmasterol 3-*O*-*β-D*-glucopyranoside | *C. callosus* | Fruits | Abd El-Fattah, Zaghloul, Halim, & Waight, 1989 |
| **19** | 3-*O*-(6′-*O*-Palmitoyl-*β-D*-glucosyl)-stigmasterol | *C. sativus* | Fruits | Tunmann & Frank, 1972 |
| **20** | 3-*O*-(6′-*O*-Stearyl-*β-D*-glucosyl)-stigmasterol | *C. sativus* | Fruits | Tunmann & Frank, 1972 |
| **21** | 22-Dehydroclerosterol; [24*β*-Ethyl-5*α*-cholesta-5,22*E*,25-trien-3*β*-ol]; [25(27)-Dehydroporiferasterol] | *C. sativus* | Seeds; Aerial parts | Akihisa (ne Itoh), Ghosh, Thakur, Rosentein, & Matsumoto, 1986; Akihisa, Thakur, Rosenstein, & Matsumoto, 1986; Garg & Nes, 1986 |
|  |  | *C. melo* | Seeds | Akihisa (ne Itoh), Ghosh, Thakur, Rosentein, & Matsumoto, 1986; Garg & Nes, 1986 |
| **22** | Isofucosterol; [24*Z*-Ethylidene-5*α*-cholest-5-en-3*β*-ol] | *C. sativus* | Seeds; Aerial parts | Akihisa (ne Itoh), Ghosh, Thakur, Rosentein, & Matsumoto, 1986; Akihisa, Thakur, Rosenstein, & Matsumoto, 1986; Garg & Nes, 1986 |
|  |  | *C. melo* | Seeds | Akihisa (ne Itoh), Ghosh, Thakur, Rosentein, & Matsumoto, 1986; Garg & Nes, 1986 |
| **23** | Clerosterol; [24*β*-Ethyl-25(27)-dehydrocholesterol]; [24*β*-Ethylcholesta-5,25-dien-3*β*-ol]; [(24*S*)-Ethylcholesta-5,25-dien-en-3*β*-ol] | *C. sativus* | Seeds; Aerial parts | Akihisa (ne Itoh), Ghosh, Thakur, Rosentein, & Matsumoto, 1986; Garg & Nes, 1986 |
|  |  | *C. melo* | Seeds | Akihisa (ne Itoh), Ghosh, Thakur, Rosentein, & Matsumoto, 1986; Garg & Nes, 1986 |
|  |  | *C. prophetarum* | Roots | Galma et al., 2021 |
| **24** | *β*-Sitosterol; [24*α*-Ethyl-5*α*-cholest-5-en-3*β*-ol]; [24*α*-Ethylcholest-5-en-3*β*-ol]; [24*α*-Ethyl-cholesterol] | *C. sativus* | Seeds; Aerial parts | Akjhisa et al., 1987; Akihisa (ne Itoh), Ghosh, Thakur, Rosentein, & Matsumoto, 1986; Akihisa, Thakur, Rosenstein, & Matsumoto, 1986; Garg & Nes, 1986; Mashchenko, Kintya, & Lazur’evskii, 1975; Matsumoto, Shigemoto, & Itoh, 1983b |
|  |  | *C. melo* | Seeds | Akihisa (ne Itoh), Ghosh, Thakur, Rosentein, & Matsumoto, 1986; Garg & Nes, 1986 |
|  |  | *C. melo* var. *reticulatus* | Seeds | Ibrahim, 2010 |
| **25** | *β*-Sitosterol 3-*O*-*β-D*-glucopyranoside | *C. melo* var. *reticulatus* | Seeds | Ibrahim, 2010 |
|  |  | *C. prophetarum* | Fruits | Afifi, Ross, Elsohly, Naeem, & Halaweish, 1999 |
|  |  | *C. callosus* | Fruits | Abd El-Fattah, Zaghloul, Halim, & Waight, 1989 |
| **26** | 3-*O*-(6′-*O*-Palmitoyl-*β-D*-glucosyl)-*β*-sitosterol | *C. sativus* | Fruits | Tunmann & Frank, 1972 |
| **27** | 3-*O*-(6′-*O*-Stearyl-*β-D*-glucosyl)-*β*-sitosterol | *C. sativus* | Fruits | Tunmann & Frank, 1972 |
| **28** | Clionasterol; [24*β*-Ethyl-cholesterol]; [24*β*-Ethyl-5*α*-cholest-5-en-3*β*-ol] | *C. sativus* | Seeds; Aerial parts | Akjhisa et al., 1987; Akihisa (ne Itoh), Ghosh, Thakur, Rosentein, & Matsumoto, 1986; Akihisa, Thakur, Rosenstein, & Matsumoto, 1986; Matsumoto, Shigemoto, & Itoh, 1983b |
|  |  | *C. melo* | Seeds | Akihisa (ne Itoh), Ghosh, Thakur, Rosentein, & Matsumoto, 1986 |
| **29** | 24*ξ*-Ethyl-cholesterol | *C. sativus* | Flowers | Knights & Smith, 1977 |
| **30** | Stellasterol; [24*α*-Methyl-5*α*-cholesta-7,22*E*-dien-3*β*-ol]; [24*α*-Methyl-22-dehydrolathosterol]; [(22*E*,24*S*)-5*α*-Ergosta-7,22-dien-3*β*-ol]; [24*α*-Methyl-5*α*-cholesta-7,22*E*-dien-3*β*-ol] | *C. sativus* | Seeds; Aerial parts | Akjhisa et al., 1987; Akihisa (ne Itoh), Ghosh, Thakur, Rosentein, & Matsumoto, 1986; Akihisa, Thakur, Rosenstein, & Matsumoto, 1986; Matsumoto, Shigemoto, & Itoh, 1983b, 1983a |
|  |  | *C. melo* | Seeds | Akjhisa et al., 1987; Akihisa (ne Itoh), Ghosh, Thakur, Rosentein, & Matsumoto, 1986 |
| **31** | 24*β*-Methyl-22-dehydrolathosterol; [24*β*-Methyl-5*α*-cholesta-7,22*E*-dien-3*β*-ol] | *C. sativus* | Seeds; Aerial parts | Akjhisa et al., 1987; Akihisa (ne Itoh), Ghosh, Thakur, Rosentein, & Matsumoto, 1986; Akihisa, Thakur, Rosenstein, & Matsumoto, 1986 |
|  |  | *C. melo* | Seeds | Akihisa (ne Itoh), Ghosh, Thakur, Rosentein, & Matsumoto, 1986 |
| **32** | 24*α*-Methyl-5*α*-cholest-7-en-3*β*-ol | *C. sativus* | Seeds; Aerial parts | Akihisa, Thakur, Rosenstein, & Matsumoto, 1986 |
| **33** | 24-Methyllathosterol; [24*β*-Methyl-5*α*-cholest-7-en-3*β*-ol]; [24*β*-Methyl-5*α*-cholest-7-en-3*β*-ol] | *C. sativus* | Seeds; Aerial parts | Akjhisa et al., 1987; Akihisa (ne Itoh), Ghosh, Thakur, Rosentein, & Matsumoto, 1986; Akihisa, Thakur, Rosenstein, & Matsumoto, 1986 |
|  |  | *C. melo* | Seeds | Akjhisa et al., 1987; Akihisa (ne Itoh), Ghosh, Thakur, Rosentein, & Matsumoto, 1986 |
| **34** | 24*ξ*-Methyllathosterol | *C. sativus*; *C. melo* | Seeds | Garg & Nes, 1986 |
| **35** | 24-Methylenelathosterol; [24-Methylene-5*α*-cholest-7-en-3*β*-ol] | *C. sativus* | Seeds; Aerial parts | Akihisa (ne Itoh), Ghosh, Thakur, Rosentein, & Matsumoto, 1986; Akihisa, Thakur, Rosenstein, & Matsumoto, 1986 |
|  |  | *C. melo* | Seeds | Akihisa (ne Itoh), Ghosh, Thakur, Rosentein, & Matsumoto, 1986 |
| **36** | *α*-Spinasterol; [24*α*-Ethyl-5*α*-cholesta-7,22*E*-dien-3*β*-ol] | *C. sativus* | Seeds; Aerial parts | Akjhisa et al., 1987; Akihisa (ne Itoh), Ghosh, Thakur, Rosentein, & Matsumoto, 1986; Akihisa, Thakur, Rosenstein, & Matsumoto, 1986; Garg & Nes, 1986; Matsumoto, Shigemoto, & Itoh, 1983b |
|  |  | *C. melo* | Seeds | Akihisa (ne Itoh), Ghosh, Thakur, Rosentein, & Matsumoto, 1986; Garg & Nes, 1986 |
|  |  | *C. melo* var. *reticulatus* | Seeds | Ibrahim, Al Haidari, Mohamed, Elkhayat, & Moustafa, 2016; Ibrahim, 2014 |
|  |  | *C. prophetarum* | Roots; Fruits | Aljohani, 2022; Galma et al., 2021 |
|  |  | *C. dipsaceus* | Fruits | Assefa et al., 2024 |
|  |  | *C. ficifolius* | Roots | Nigussie & Ashenef, 2020 |
| **37** | *α*-Spinasterol 3-*O*-*β-D*-glucopyranoside | *C. sativus* | Roots; Stems | Zhou et al., 2012 |
| **38** | Chondrillasterol; [24*β*-Ethyl-5*α*-cholesta-7,22*E*-dien-3*β*-ol] | *C. sativus* | Seeds; Aerial parts | Akjhisa et al., 1987; Akihisa (ne Itoh), Ghosh, Thakur, Rosentein, & Matsumoto, 1986; Akihisa, Thakur, Rosenstein, & Matsumoto, 1986; Matsumoto, Shigemoto, & Itoh, 1983b |
|  |  | *C. melo* | Seeds | Akihisa (ne Itoh), Ghosh, Thakur, Rosentein, & Matsumoto, 1986 |
|  |  | *C. callosus* | Fruits | Abd El-Fattah, Zaghloul, Halim, & Waight, 1989 |
| **39** | 25(27)-Dehydrochondrillasterol; [24*β*-Ethyl-22,25(27)-bisdehydrolathosterol]; [24*β*-Ethyl-5*α*-cholesta-7,22*E*,25-trien-3*β*-ol] | *C. sativus* | Seeds; Aerial parts | Akjhisa et al., 1987; Akihisa (ne Itoh), Ghosh, Thakur, Rosentein, & Matsumoto, 1986; Akihisa, Thakur, Rosenstein, & Matsumoto, 1986; Garg & Nes, 1986; Matsumoto, Shigemoto, & Itoh, 1983b |
|  |  | *C. melo* | Seeds | Akihisa (ne Itoh), Ghosh, Thakur, Rosentein, & Matsumoto, 1986; Garg & Nes, 1986 |
| **40** | 24*β*-Ethyl-5*α*-cholesta-7,22,25(27)-trien-3*β*-ol 3-*O*-*β-D*-glucopyranoside | *C. sativus* | Seeds | Mashchenko, Kintya, & Lazur’evskii, 1975 |
| **41** | 24*ξ*-Ethyl-5*α*-cholest-7,25-dien-3*β*-ol | *C. sativus* | Flowers | Knights & Smith, 1977 |
| **42** | 24*ξ*-Ethyl-5*α*-cholest-7,25-dien-3*β*-ol acetate | *C. sativus* | Seeds | Kintia & Wojciechowski, 1975 |
| **43** | ∆^7^-Avenasterol; [(24*Z*)-Ethylidenelathosterol]; [(24*Z*)-Ethylidene-5*α*-cholest-7-en-3*β*-ol]; [(24*Z*)-Ethyl-5*α*-cholesta-7,24(28)-dien-3*β*-ol] | *C. sativus* | Seeds; Aerial parts; Flowers | Akihisa (ne Itoh), Ghosh, Thakur, Rosentein, & Matsumoto, 1986; Akihisa, Thakur, Rosenstein, & Matsumoto, 1986; Garg & Nes, 1986; Knights & Smith, 1977 |
|  |  | *C. melo* | Seeds | Akihisa (ne Itoh), Ghosh, Thakur, Rosentein, & Matsumoto, 1986; Garg & Nes, 1986 |
| **44** | 24*β*-Ethyl-25(27)-dehydrolathosterol; [24*β*-Ethyl-5*α*-cholesta-7,25(27)-dien-3*β*-ol]; [24*β*-Ethyl-5*α*-cholesta-7,25-dien-3*β*-ol] | *C. sativus* | Seeds; Aerial parts | Akjhisa et al., 1987; Akihisa (ne Itoh), Ghosh, Thakur, Rosentein, & Matsumoto, 1986; Akihisa, Thakur, Rosenstein, & Matsumoto, 1986; Garg & Nes, 1986; Matsumoto, Shigemoto, & Itoh, 1983b |
|  |  | *C. melo* | Seeds | Akihisa (ne Itoh), Ghosh, Thakur, Rosentein, & Matsumoto, 1986; Garg & Nes, 1986 |
| **45** | 24*β*-Ethyllathosterol; [24*β*-Ethyl-5*α*-cholest-7-en-3*β*-ol]; [22-Dihydrochondrillasterol] | *C. sativus* | Seeds; Aerial parts | Akjhisa et al., 1987; Akihisa (ne Itoh), Ghosh, Thakur, Rosentein, & Matsumoto, 1986; Akihisa, Thakur, Rosenstein, & Matsumoto, 1986; Matsumoto, Shigemoto, & Itoh, 1983b |
|  |  | *C. melo* | Seeds | Akjhisa et al., 1987; Akihisa (ne Itoh), Ghosh, Thakur, Rosentein, & Matsumoto, 1986 |
| **46** | 22-Dihydrospinasterol; [24*α*-Ethyllathosterol]; [24*α*-Ethyl-5*α*-cholest-7-en-3*β*-ol]; [24*α*-Ethyl-5*α*-cholest-7-en-3*β*-ol]; [Stigma-7-en-3*β*-ol]; [Δ^7^-(3*β*)-Stigmastenol]; [Stigmast-7-en-3*β*-ol] | *C. sativus* | Seeds; Leaves; Aerial parts | Akjhisa et al., 1987; Akihisa (ne Itoh), Ghosh, Thakur, Rosentein, & Matsumoto, 1986; Akihisa, Thakur, Rosenstein, & Matsumoto, 1986; Garg & Nes, 1986; Matsumoto, Shigemoto, & Itoh, 1983b |
|  |  | *C. melo* | Seeds | Akjhisa et al., 1987; Akihisa (ne Itoh), Ghosh, Thakur, Rosentein, & Matsumoto, 1986; Garg & Nes, 1986 |
|  |  | *C. trigonus* | Fruits | Ulubelen, Baytop, & Çubukcu, 1976 |
| **47** | Stigmast-7-en 3-*O*-*β-D*-glucopyranoside | *C. sativus* | Stems | Akihisa (ne Itoh), Ghosh, Thakur, Rosentein, & Matsumoto, 1986 |
|  |  | *C. trigonus* | Fruits | Ulubelen, Baytop, & Çubukcu, 1976 |
| **48** | 24*ξ*-Ethyl-5*α*-cholest-7-en-3*β*-ol | *C. sativus* | Flowers | Knights & Smith, 1977 |
| **49** | 24*ξ*-Ethyl-5*α*-cholesta-7,22-dien-3*β*-ol | *C. sativus* | Flowers | Knights & Smith, 1977 |
| **50** | Peposterol; [24-Ethyl-24(25)-dehydrolathosterol]; [24-Ethyl-5*α*-cholesta-7,24-dien-3*β*-ol] | *C. sativus* | Seeds; Aerial parts | Akihisa (ne Itoh), Ghosh, Thakur, Rosentein, & Matsumoto, 1986; Akihisa, Thakur, Rosenstein, & Matsumoto, 1986 |
| **51** | 28-Isoavenasterol; [(24*E*)-Ethylidenelathosterol]; [24*E*-Ethylidene-5*α*-cholest-7-en-3*β*-ol] | *C. sativus* | Seeds; Aerial parts | Akihisa (ne Itoh), Ghosh, Thakur, Rosentein, & Matsumoto, 1986; Akihisa, Thakur, Rosenstein, & Matsumoto, 1986 |
|  |  | *C. melo* | Seeds | Akihisa (ne Itoh), Ghosh, Thakur, Rosentein, & Matsumoto, 1986 |
| **52** | Stigmasta-7,22,25-trien-3*β*-ol | *C. melo* var. *reticulatus* | Seeds | Ibrahim, 2014 |
| **53** | Stigmasta-7,22,25-trien-3*β*-ol 3-*O*-*β-D*-glucopyranoside; [Glycoside A] | *C. sativus* | Seeds; Fruit peels | Mashchenko, Kintya, & Lazur’evskii, 1975; Mashchenko et al., 1976 |
| **54** | Δ^7^-Stigmastenon; [Stigmast-7-en-3-one] | *C. sativus*; *C. melo* | Seeds | Akjhisa et al., 1987; Akihisa (ne Itoh), Ghosh, Thakur, Rosentein, & Matsumoto, 1986 |
| **55** | 24*α*-Ethyl-5*α*-cholesta-8,22*E*-dien-3*β*-ol | *C. sativus* | Seeds; Aerial parts | Akjhisa et al., 1987; Akihisa (ne Itoh), Ghosh, Thakur, Rosentein, & Matsumoto, 1986; Akihisa, Thakur, Rosenstein, & Matsumoto, 1986 |
|  |  | *C. melo* | Seeds | Akihisa (ne Itoh), Ghosh, Thakur, Rosentein, & Matsumoto, 1986 |
| **56** | 24*β*-Ethyl-5*α*-cholesta-8,22*E*-dien-3*β*-ol | *C. sativus* | Seeds; Aerial parts | Akjhisa et al., 1987; Akihisa, Thakur, Rosenstein, & Matsumoto, 1986 |
| **57** | 24*β*-Ethyl-5*α*-cholesta-8,22*E*,25-trien-3*β*-ol | *C. sativus* | Seeds; Aerial parts | Akihisa (ne Itoh), Ghosh, Thakur, Rosentein, & Matsumoto, 1986; Akihisa, Thakur, Rosenstein, & Matsumoto, 1986 |
|  |  | *C. melo* | Seeds | Akihisa (ne Itoh), Ghosh, Thakur, Rosentein, & Matsumoto, 1986 |
| **58** | 24*β*-Ethyl-5*α*-cholesta-8,25-dien-3*β*-ol | *C. sativus* | Seeds; Aerial parts | Akihisa (ne Itoh), Ghosh, Thakur, Rosentein, & Matsumoto, 1986; Akihisa, Thakur, Rosenstein, & Matsumoto, 1986 |
|  |  | *C. melo* | Seeds | Akihisa (ne Itoh), Ghosh, Thakur, Rosentein, & Matsumoto, 1986 |
| **59** | 24*α*-Ethyl-5*α*-cholesta-8(14),22*E*-dien-3*β*-ol | *C. sativus* | Seeds; Aerial parts | Akjhisa et al., 1987; Akihisa (ne Itoh), Ghosh, Thakur, Rosentein, & Matsumoto, 1986 |
| **60** | 24*α*-Ethyl-5*α*-cholest-8(14)-en-3*β*-ol | *C. sativus* | Seeds; Aerial parts | Akihisa (ne Itoh), Ghosh, Thakur, Rosentein, & Matsumoto, 1986 |
| **61** | Stigmasta-8(14),22-diene-7*α*-methoxy-3*β*-ol | *C. sativus* | Roots | Zhou et al., 2012 |
| **62** | (24*R*)-14*α*-Methyl-24-ethyl-5*α*-cholest-9(11)-en-3*β*-ol; [14*α*-Methyl-24*α*-ethyl-5*α*-cholest-9(11)-en-3*β*-ol] | *C. sativus* | Aerial parts | Akjhisa et al., 1987; Akihisa, Shimizu, Tamura, & Matsumoto, 1986 |
| **63** | Ergosterol | *C. sativus* | Roots | Zhou et al., 2012 |
| **64** | 24-Methylenepollinastanol; [14*α*-Methyl-24-methylene-9*β*,19-cyclo-5*α*-cholestan-3*β*-ol] | *C. sativus* | Aerial parts | Akjhisa et al., 1987; Akihisa, Shimizu, Tamura, & Matsumoto, 1986 |
| **65** | Stigmasta-4,6,8(14),22-tetraen-3-one | *C. sativus* | Roots | Zhou et al., 2012 |
| **66** | Cucumidisecosterol | *C. prophetarum* subsp. *dissectus* | Fruits | Al-Rehaily, Al-Yahya, Mirza, & Ahmed, 2002 |
| **67** | Cycloeucalenol; [4*α*,14*α*,24-Trimethyl-9*β*,19-cyclo-5*α*-cholest-24(28)-en-3*β*-ol] | *C. sativus* | Seeds | Kintia & Wojciechowski, 1975 |
| **68** | 24-Methylenelophenol | *C. sativus* | Seeds | Kintia & Wojciechowski, 1975 |
| **69** | 24*β*-Ethyl-25(27)-dehydrolophenol; [4*α*-Methyl-24*β*-ethyl-5*α*-cholesta-7,25(27)-dien-3*β*-ol] | *C. sativus* | Seeds | Itoh, Kikuchi, Shimizu, Tamura, & Matsumoto, 1981 |
| **70** | 24-Ethylidenelophenol | *C. sativus* | Seeds | Kintia & Wojciechowski, 1975 |
| **71** | 24*β*-Ethyl-31-norlanosta-8,25(27)-dien-3*β*-ol; [4*α*,14*α*-Dimethyl-24*β*-ethyl-5*α*-cholesta-8,25(27)-dien-3*β*-ol] | *C. sativus* | Seeds | Itoh, Kikuchi, Shimizu, Tamura, & Matsumoto, 1981 |
| **72** | Obtusifoliol; [4*α*,14*α*,24-Trimethyl-5*α*-cholesta-8,24(28)-dien-3*β*-ol] | *C. sativus* | Seeds | Kintia & Wojciechowski, 1975 |
| **73** | 4,4-Dimethyl-stigmast-5-22*E*,24-trien-3*β*-ol | *C. prophetarum* | Roots | Tamiru, Temesegen, & Demise, 2019 |
| Triterpenoids (102 compounds) | | | | |
| **74** | 10*α*-Cucurbita-5,24-dien-3*β*-ol | *C. sativus*; *C. melo* | Seeds | Shang et al., 2014 |
| **75** | Cucurbita-5,24-diene-3*β*,19-diol | *C. sativus* | Leaves | Shang et al., 2014 |
| **76** | Cucurbita-5,23-diene-3,19,25-triol | *C. sativus* | Leaves | Shang et al., 2014 |
| **77** | Cucurbitacin C | *C. sativus* | Leaves; Stems | Chen et al., 2022; Qing et al., 2022 |
|  |  | *C. prophetarum* | Fruits | Rao & Row, 1968 |
| **78** | Cucurbitacin C 3-*O*-*β-D*-glucopyranoside | *C. sativus* | Leaves | Chen et al., 2022 |
| **79** | 23,24-Dihydrocucurbitacin C | *C. sativus* | Leaves | Chen et al., 2022; Qing et al., 2022 |
| **80** | Deacetyl-cucurbitacin C | *C. sativus* | Leaves | Chen et al., 2022; Qing et al., 2022; Shang et al., 2014 |
| **81** | Deacetyl-cucurbitacin C 3-*O*-*β-D*-glucopyranoside | *C. sativus* | Leaves | Chen et al., 2022 |
| **82** | Isocucurbitacin C | *C. sativus* | Leaves | Chen et al., 2022 |
| **83** | 23,24-Dihydro-isocucurbitacin C | *C. sativus* | Leaves | Chen et al., 2022 |
| **84** | Cucurbitacin C_1_ | *C. sativus* | Leaves | Qing et al., 2022 |
| **85** | Cucurbitacin C_2_ | *C. sativus* | Leaves | Qing et al., 2022 |
| **86** | Cucurbitacin C_3_ | *C. sativus* | Leaves | Qing et al., 2022 |
| **87** | Cucurbitacin C_4_ | *C. sativus* | Leaves | Qing et al., 2022 |
| **88** | Cucurbitacin C_5_ | *C. sativus* | Leaves | Qing et al., 2022 |
| **89** | Cucurbitacin C_6_ | *C. sativus* | Leaves | Qing et al., 2022 |
| **90** | Cucurbitacin C_7_ | *C. sativus* | Leaves | Qing et al., 2022 |
| **91** | Cucurbitacin B^(*)^ | *C. sativus* | Leaves; Fruits | Kintya, Isaeva, Chirva, & Lazur’evskii, 1972 |
|  |  | *C. melo* | Stems; Fruits; Pedicels | Chen, Qiang, Lou, & Zhao, 2009; Chen et al., 2014; Du, Xiong, & Ito, 1995; Jianhua, Kaiyue, Huan, Lan, & Lijuan, 2013; Yuan et al., 2019 |
|  |  | *C. prophetarum* | Fruits | Afifi, Ross, Elsohly, Naeem, & Halaweish, 1999 |
|  |  | *C. prophetarum* subsp. *dissectus* | Fruits | Afifi, Ross, Elsohly, Naeem, & Halaweish, 1999 |
|  |  | *C. prophetarum* var. *prophetarum* | Fruits | Alsayari et al., 2018 |
|  |  | *C. callosus* | Fruits; Pericarp | Abd El-Fattah, Zaghloul, Halim, & Waight, 1989; Deepika et al., 2023 |
|  |  | *C. africanus* | Fruits | Rehm, Enslin, Meeuse, & Wessels, 1957 |
|  |  | *C. ficifolius* | Fruits; Roots | Nigussie & Ashenef, 2020; Rehm, Enslin, Meeuse, & Wessels, 1957 |
|  |  | *C. metuliferus* | Fruits | Rehm, Enslin, Meeuse, & Wessels, 1957 |
|  |  | *C. longipes* | Fruits | Rehm, Enslin, Meeuse, & Wessels, 1957 |
| **92** | Cucurbitacide E | *C. sativus* | Seeds | Kintya, Isaeva, Chirva, & Lazur’evskii, 1972 |
| **93** | Arvenin I; [Cucurbitacin B 2-*O*-*β-D*-glucopyranoside] | *C. melo* | Stems; Pedicels | Chen, Qiang, Lou, & Zhao, 2009; Chen et al., 2014; Jianhua, Kaiyue, Huan, Lan, & Lijuan, 2013 |
| **94** | Cucurbitacin F^(*)^ | *C. angolensis*; *C. dinteri* | Leaves | Rehm, Enslin, Meeuse, & Wessels, 1957 |
| **95** | Cucurbitacin E; [*α*-Elaterin] | *C. sativus* | Leaves; Stems; Seeds | Maja, Mavengahama, & Mashilo, 2022 |
|  |  | *C. melo* | Fruits; Pedicels | Du, Xiong, & Ito, 1995; Jianhua, Kaiyue, Huan, Lan, & Lijuan, 2013; Yuan et al., 2019 |
|  |  | *C. prophetarum* | Fruits | Afifi, Ross, Elsohly, Naeem, & Halaweish, 1999 |
|  |  | *C. prophetarum* var. *prophetarum* | Fruits | Alsayari et al., 2018 |
| **96** | Cucurbitacin E 2-*O*-*β-D*-glucopyranoside | *C. prophetarum* | Fruits | Aljohani, 2022 |
|  |  | *C. sativus*; *C. melo*; *C. melo* var. *agrestis*; *C. anguria* | Whole plant | Ul Haq et al., 2019 |
| **97** | Cucurbitacin I 2-*O*-*β-D*-glucopyranoside | *C. sativus*; *C. melo*; *C. melo* var. *flexuosus*; *C. melo* var. *agrestis*; *C. anguria* | Whole plant | Ul Haq et al., 2019 |
| **98** | Cucurbitacin Q | *C. sativus*; *C. melo*; *C. melo* var. *agrestis*; *C. anguria* | Whole plant | Ul Haq et al., 2019 |
| **99** | 22-Deoxocucurbitoside B; [20-Hydroxy-2*β*-{*O*-*α*-*L*-rhamnopyranosyl-(1″→2′)-*α-D*-glucopyranosyloxy}-16*α*,23*α*-epoxycucirbita-1,5,24-triene-3,11-dione] | *C. sativus*; *C. melo*; *C. melo* var. *flexuosus*; *C. melo* var. *agrestis*; *C. anguria* | Whole plant | Ul Haq et al., 2019 |
| **100** | Cucurbitacin A^(*)^ | *C. melo* | Stems | Chen, Qiang, Lou, & Zhao, 2009 |
|  |  | *C. prophetarum* subsp. *dissectus* | Fruits | Al-Rehaily, Al-Yahya, Mirza, & Ahmed, 2002 |
|  |  | *C. myriocarpus*; *C. leptodermis* | Fruits | Rehm, Enslin, Meeuse, & Wessels, 1957 |
| **101** | Cucurbitacin A 2-*O*-*β-D*-glucopyranoside | *C. melo* | Stems | Chen, Qiang, Lou, & Zhao, 2009 |
| **102** | 25-Deacetylcucurbitacin A | *C. melo* | Stems | Chen, Qiang, Lou, & Zhao, 2009 |
| **103** | 23,24-Dihydro-25-deacetylcucurbitacin A | *C. melo* | Stems | Chen, Qiang, Lou, & Zhao, 2009 |
| **104** | 7*β*-Hydroxycucurbitacin B | *C. melo* | Stems | Chen, Qiang, Lou, & Zhao, 2009 |
| **105** | 23,24-Dihydro-7*β*-hydroxycucurbitacin B | *C. melo* | Stems | Chen, Qiang, Lou, & Zhao, 2009 |
| **106** | 23,24-Dihydrocucurbitacin B | *C. melo* | Stems; Pedicels | Chen, Qiang, Lou, & Zhao, 2009; Jianhua, Kaiyue, Huan, Lan, & Lijuan, 2013 |
|  |  | *C. prophetarum* | Fruits | Afifi, Ross, Elsohly, Naeem, & Halaweish, 1999 |
| **107** | 23,24-Dihydro-isocucurbitacin B | *C. melo* | Stems; Pedicels | Chen, Qiang, Lou, & Zhao, 2009; Jianhua, Kaiyue, Huan, Lan, & Lijuan, 2013 |
|  |  | *C. prophetarum* | Fruits | Afifi, Ross, Elsohly, Naeem, & Halaweish, 1999 |
| **108** | Cucurbitacin D^(*)^; [Elatericin A] | *C. melo* | Pedicels | Chen et al., 2014; Jianhua, Kaiyue, Huan, Lan, & Lijuan, 2013; Yuan et al., 2019 |
|  |  | *C.* *prophetarum* | Fruits | Rehm, Enslin, Meeuse, & Wessels, 1957 |
|  |  | *C. prophetarum* var. *prophetarum* | Fruits | Alsayari et al., 2018 |
|  |  | *C. callosus* | Fruits | Abd El-Fattah, Zaghloul, Halim, & Waight, 1989 |
|  |  | *C. dipsaceus* | Fruits | Assefa et al., 2024 |
|  |  | *C. ficifolius* | Roots | Nigussie & Ashenef, 2020 |
|  |  | *C. angolensis*; *C. dinteri* | Fruits | Rehm, Enslin, Meeuse, & Wessels, 1957 |
|  |  | *C. hirsutus*; *C. longipes* | Roots | Rehm, Enslin, Meeuse, & Wessels, 1957 |
| **109** | Arvenin III; [Cucurbitacin D 2-*O*-*β*-D-glucopyranoside] | *C. melo* | Stems | Chen, Qiang, Lou, & Zhao, 2009 |
| **110** | Cucurbitacin G^(*)^ | *C. melo* | Stems | Chen, Qiang, Lou, & Zhao, 2009 |
|  |  | *C. prophetarum* | Fruits | Rehm, Enslin, Meeuse, & Wessels, 1957 |
|  |  | *C. hirsutus* | Roots | Rehm, Enslin, Meeuse, & Wessels, 1957 |
| **111** | Cucurbitacin H^(*)^ | *C. melo* | Stems | Chen, Qiang, Lou, & Zhao, 2009 |
|  |  | *C. prophetarum* | Fruits | Rehm, Enslin, Meeuse, & Wessels, 1957 |
|  |  | *C. hirsutus* | Roots | Rehm, Enslin, Meeuse, & Wessels, 1957 |
| **112** | Cucurbitacin R; [23,24-Dihydrocucurbitacin D] | *C. melo* | Stems | Chen, Qiang, Lou, & Zhao, 2009 |
|  |  | *C. prophetarum* | Fruits | Afifi, Ross, Elsohly, Naeem, & Halaweish, 1999; Alsayari et al., 2018 |
| **113** | Isocucurbitacin R; [23,24-Dihydro-isocucurbitacin D] | *C. melo* | Stems | Chen, Qiang, Lou, & Zhao, 2009 |
|  |  | *C. prophetarum* | Fruits | Afifi, Ross, Elsohly, Naeem, & Halaweish, 1999 |
|  |  | *C. dipsaceus* | Fruits | Assefa et al., 2024 |
| **114** | 19-Norlanosta-5,24-dien-11-one | *C. melo* | Stems | Chen, Qiang, Lou, & Zhao, 2009 |
| **115** | 16*α*,23*α*-Epoxy-2*β*,3*β*,7*β*,20*β*,26-pentahydroxy-10*α*,23*α*-cucurbit-5,24-(*E*)-dien-11-one | *C. melo* | Stems | Chen, Qiang, Lou, & Zhao, 2009 |
| **116** | 16*α*,23*α*-Epoxy-2*β*,3*β*,7*β*,20*β*,26-pentahydroxy-10*α*,23*α*-cucurbit-5,24-(*E*)-dien-11-one 2-*O*-*β-D*-glucopyranoside | *C. melo* | Stems | Chen, Qiang, Lou, & Zhao, 2009 |
| **117** | 2*β*,16*α*,20,23,26-Pentahydroxy-10*α*-cucurbit-5,24-(*E*)-diene-3,11-dione | *C. melo* | Stems | Chen, Qiang, Lou, & Zhao, 2009 |
| **118** | Hexanorcucurbitacin D 2-*O*-*β-D*-glucopyranoside | *C. melo* | Stems | Chen, Qiang, Lou, & Zhao, 2009 |
| **119** | Hexanorcucurbitacin D | *C. melo* | Stems | Chen, Qiang, Lou, & Zhao, 2009 |
|  |  | *C. prophetarum* var. *prophetarum* | Fruits | Alsayari et al., 2018 |
| **120** | Isocucurbitacin B | *C. melo* | Pedicels | Jianhua, Kaiyue, Huan, Lan, & Lijuan, 2013 |
|  |  | *C. prophetarum* | Fruits | Afifi, Ross, Elsohly, Naeem, & Halaweish, 1999 |
| **121** | Cucurbitacin I; [Elatericin B] | *C. melo* | Pedicels | Jianhua, Kaiyue, Huan, Lan, & Lijuan, 2013 |
|  |  | *C. prophetarum* | Fruits | Afifi, Ross, Elsohly, Naeem, & Halaweish, 1999 |
| **122** | Isocucurbitacin D | *C. prophetarum* | Fruits | Afifi, Ross, Elsohly, Naeem, & Halaweish, 1999 |
| **123** | 23,24-Dihydrocucurbitacin E | *C. prophetarum* | Fruits | Afifi, Ross, Elsohly, Naeem, & Halaweish, 1999 |
| **124** | 23,24-Dihydrocucurbitacin I | *C. prophetarum* | Fruits | Afifi, Ross, Elsohly, Naeem, & Halaweish, 1999 |
| **125** | Cucurbitacin Q_1_; [Cucurbitacin F 25-*O*-acetate] | *C. prophetarum* | Fruits; Leaves; Stems | Afifi, Ross, Elsohly, Naeem, & Halaweish, 1999; Atta-ur-Rahman, Ahmed, Khan, & Zehra, 1973 |
|  |  | *C. prophetarum* subsp. *dissectus* | Fruits | Afifi, Ross, Elsohly, Naeem, & Halaweish, 1999 |
|  |  | *C. prophetarum* var. *prophetarum* | Fruits | Alsayari et al., 2018 |
|  |  | *C. callosus* | Fruits | Abd El-Fattah, Zaghloul, Halim, & Waight, 1989 |
| **126** | 23,24-Dihydrocucurbitacin Q_1_; [23,24-Dihydrocucurbitacin F 25-*O*-acetate] | *C. prophetarum* | Fruits; Leaves; Stems | Afifi, Ross, Elsohly, Naeem, & Halaweish, 1999 |
|  |  | *C. prophetarum* subsp. *dissectus* | Fruits | Afifi, Ross, Elsohly, Naeem, & Halaweish, 1999 |
| **127** | Cucurbitacin O | *C. prophetarum* subsp. *dissectus* | Fruits | Afifi, Ross, Elsohly, Naeem, & Halaweish, 1999 |
| **128** | Cucurbitacin P; [Dihydrocucurbitacin O] | *C. prophetarum* subsp. *dissectus* | Fruits | Afifi, Ross, Elsohly, Naeem, & Halaweish, 1999 |
| **129** | Isocucurbitacin E | *C. prophetarum* subsp. *dissectus* | Fruits | Afifi, Ross, Elsohly, Naeem, & Halaweish, 1999 |
| **130** | 23,24-Dihydro-isocucurbitacin E | *C. prophetarum* subsp. *dissectus* | Fruits | Afifi, Ross, Elsohly, Naeem, & Halaweish, 1999 |
| **131** | 7,8,11,12,14,15,16,17-Octahydro-17-[(*E*)-6-hydroxy-6-methylhept-3-en-2-yl]-9,13-dimethyl-6*H*-cyclopenta[a]phenanthren-7-yl acetate | *C. prophetarum* | Roots | Tamiru, Temesegen, & Demise, 2019 |
| **132** | Cucurbitacin-1 | *C. prophetarum* | Roots | Galma et al., 2021 |
| **133** | Cucurbitacin-2 | *C. prophetarum* | Roots | Galma et al., 2021 |
| **134** | 16-Dehydroxycucurbitacin D | *C. dipsaceus* | Fruits | Assefa et al., 2024 |
| **135** | Cycloartenol | *C. sativus*; *C. melo* | Seeds | Akihisa et al., 1988; Itoh, Shigemoto, Shimizu, Tamura, & Matsumoto, 1982; Kintia & Wojciechowski, 1975 |
| **136** | Cycloartanol; [9*β*,19-Cyclolanostanol] | *C. sativus* | Seeds | Akihisa et al., 1988 |
| **137** | (23*Z*)-Cycloart-23-ene-3*β*,25-diol | *C. sativus* | Seeds | Akihisa et al., 1997 |
| **138** | 22-Methylene-9,19-cyclolanostan-3*β*-ol | *C. sativus* | Stems | Akihisa (ne Itoh), Ghosh, Thakur, Rosentein, & Matsumoto, 1986 |
| **139** | 24-Methylenecycloartanol | *C. sativus*; *C. melo* | Seeds | Akihisa et al., 1988; Itoh, Shigemoto, Shimizu, Tamura, & Matsumoto, 1982; Kintia & Wojciechowski, 1975 |
| **140** | 24-Methyl-25(27)-dehydrocycloartanol | *C. sativus*; *C. melo* | Seeds | Itoh, Shigemoto, Shimizu, Tamura, & Matsumoto, 1982 |
| **141** | 24-Methylene-24-dihydrolanosterol; [24-Methylenelanost-8-enol] | *C. sativus*; *C. melo* | Seeds | Akihisa et al., 1988; Itoh, Shigemoto, Shimizu, Tamura, & Matsumoto, 1982 |
| **142** | 24-Methylene-24-dihdyroparkeol; [24-Methylenelanost-9(11)-enol] | *C. sativus*; *C. melo* | Seeds | Akihisa et al., 1988; Itoh, Shigemoto, Shimizu, Tamura, & Matsumoto, 1982 |
| **143** | Dehydroeburicoic acid | *C. sativus* | Roots | Zhou et al., 2012 |
| **144** | Euphol; [Eupha-8,24-dienol] | *C. sativus*; *C. melo* | Seeds | Akihisa et al., 1988; Itoh, Shigemoto, Shimizu, Tamura, & Matsumoto, 1982 |
| **145** | Butyrospermol; [Eupha-7,24-dienol] | *C. sativus*; *C. melo* | Seeds | Akihisa et al., 1988 |
| **146** | Tirucallol; [Tirucalla-8,24-dienol] | *C. sativus*; *C. melo* | Seeds | Akihisa et al., 1988; Itoh, Shigemoto, Shimizu, Tamura, & Matsumoto, 1982 |
| **147** | 17-{5-Ethyl-2,6-dihydroxy-6-methylhept-3-en-2-yl}-9-(hydroxymethyl)-13-methylcyclopenta[*α*]phenanthren-3-ol | *C. dipsaceus* | Fruits | Assefa et al., 2024 |
| **148** | 3,29-*O*-Dibenzoylmultiflora-8-en-3*α*,7*β*,29-triol | *C. melo* var. *inodorus* | Seeds | De Marino, Festa, Zollo, & Iorizzi, 2009 |
| **149** | 3-*O*-*p*-Aminobenzoyl-29-*O*-benzoylmultiflora-8-ene-3*α*,7*β*,29-triol | *C. sativus* | Seeds | Appendino, Jakupovic, Belloro, & Marchesini, 1999 |
|  |  | *C. melo* var. *inodorus* | Seeds | De Marino, Festa, Zollo, & Iorizzi, 2009 |
| **150** | 7-Oxodihydrokarounidiol | *C. sativus*; *C. melo* | Seeds | Akihisa et al., 1997 |
| **151** | 7-Oxodihydrokarounidiol-3-benzoate | *C. sativus*; *C. melo* | Seeds | Akihisa et al., 1997 |
| **152** | Isokarounidiol | *C. sativus*; *C. melo* | Seeds | Akihisa et al., 1997 |
| **153** | 3*β*-Hydroxymultiflora-8-en-17-oic acid | *C. sativus* | Roots | Zhou et al., 2012 |
| **154** | Isomultiflorenol; [D:C-Friedoolean-8-enol] | *C. sativus*; *C. melo* | Seeds | Akihisa et al., 1988; Itoh, Shigemoto, Shimizu, Tamura, & Matsumoto, 1982 |
| **155** | Bryonolol | *C. sativus*; *C. melo* | Seeds | Akihisa et al., 1997 |
| **156** | Bryonolic acid | *C. sativus*; *C. melo* | Roots | Akiyama & Hayashi, 2002 |
| **157** | 2*β*-Hydroxybryonolic acid | *C. sativus* | Roots | Akiyama & Hayashi, 2002 |
| **158** | 3-*O*-*p*-Aminobenzoyl-29-*O*-benzoylmultiflora-7,9(11)-diene-3*α*,29-diol | *C. sativus* | Seeds | Appendino, Jakupovic, Belloro, & Marchesini, 1999 |
| **159** | Karounidiol; [Multiflora-7,9(11)-diene-3*α*,29-diol] | *C. sativus* | Roots; Seeds | Akihisa et al., 1997; Zhou et al., 2012 |
|  |  | *C. melo* | Seeds | Akihisa et al., 1997 |
| **160** | Karounidiol-3-benzoate | *C. sativus*; *C. melo* | Seeds | Akihisa et al., 1997 |
| **161** | 5-Dehydrokarounidiol | *C. sativus*; *C. melo* | Seeds | Akihisa et al., 1997 |
| **162** | 3*β*-Bryoferulic acid | *C. sativus* | Roots | Akiyama & Hayashi, 2002 |
| **163** | Multiflorenol; [D:C-Friedoolean-7-en-3*β*-ol] | *C. sativus*; *C. melo* | Seeds | Akihisa et al., 1988; Itoh, Shigemoto, Shimizu, Tamura, & Matsumoto, 1982 |
| **164** | Erythrodiol | *C. dipsaceus* | Fruits | Assefa et al., 2024 |
| **165** | Cucumol A | *C. melo* var. *reticulatus* | Seeds | Ibrahim, Al Haidari, Mohamed, Elkhayat, & Moustafa, 2016 |
| **166** | Cucumol B | *C. melo* var. *reticulatus* | Seeds | Ibrahim et al., 2019 |
| **167** | Taraxerol | *C. sativus*; *C. melo* | Seeds | Akihisa et al., 1988; Itoh, Shigemoto, Shimizu, Tamura, & Matsumoto, 1982 |
| **168** | *β*-Amyrin; [Olean-12-en-3*β*-ol] | *C. sativus*; *C. melo* | Seeds | Akihisa et al., 1988; Itoh, Shigemoto, Shimizu, Tamura, & Matsumoto, 1982; Kintia & Wojciechowski, 1975 |
|  |  | *C. melo* var. *reticulatus* | Seeds | Ibrahim, 2010 |
| **169** | Glutinol; [D:B-Friedoolean-5-en-3*β*-ol] | *C. melo* var. *reticulatus* | Seeds | Ibrahim, Al Haidari, Mohamed, Elkhayat, & Moustafa, 2016; Ibrahim, 2014 |
|  |  | *C. melo* | Pedicels | Chen et al., 2014 |
| **170** | Alnusenol | *C.* *trigonus* | Fruits | Ulubelen, Baytop, & Çubukcu, 1976 |
| **171** | Alnusenone | *C. trigonus* | Fruits | Ulubelen, Baytop, & Çubukcu, 1976 |
| **172** | Lupeol; [Lup-20(29)-enol] | *C. sativus*; *C. melo* | Seeds | Akihisa et al., 1988; Itoh, Shigemoto, Shimizu, Tamura, & Matsumoto, 1982 |
| **173** | Loranthol; [Lup-20(30)-ene-3*β*,7*β*-diol] | *C. melo* | Seeds | Akihisa et al., 1997 |
| **174** | *α*-Amyrin; [Urs-12-enol] | *C. sativus*; *C. melo* | Seeds | Akihisa et al., 1988; Itoh, Shigemoto, Shimizu, Tamura, & Matsumoto, 1982; Kintia & Wojciechowski, 1975 |
| **175** | Ursolic acid | *C. sativus* | Roots | Zhou et al., 2012 |
|  |  | *C.* *metuliferus* | Fruits | Busuioc, Costea, Botezatu, Furdui, & Dinica, 2023 |
| Flavonoids (90 compounds) | | | | |
| **176** | Apigenin | *C. sativus* | Leaves | Jevtić et al., 2017 |
|  |  | *C. melo* | Fruit peels | Gómez-García, Campos, Aguilar, Madureira, & Pintado, 2020 |
| **177** | Apigetrin | *C. sativus* | Leaves | Jevtić et al., 2017 |
|  |  | *C. melo* | Fruit peels | Gómez-García, Campos, Aguilar, Madureira, & Pintado, 2020 |
| **178** | Isovitexin | *C. sativus* | Leaves | Mukherjee, Nema, Maity, & Sarkar, 2013 |
|  |  | *C. myriocarpus* | ND | Krauze-Baranowska & Cisowski, 2001 |
| **179** | Saponarin | *C. sativus* | Leaves | Mukherjee, Nema, Maity, & Sarkar, 2013 |
| **180** | Saponarin 4′-*O*-glucoside | *C. sativus* | Leaves | Mukherjee, Nema, Maity, & Sarkar, 2013 |
| **181** | Isovitexin 2″-*O*-(6‴-*O*-(*E*)-feruloyl)-glucopyranoside | *C. sativus* | Leaves | Mukherjee, Nema, Maity, & Sarkar, 2013 |
|  |  | *C. melo* var. *reticulatus* | Leaves | Hosoya, Masuda, Ohba, & Kumazawa, 2024 |
| **182** | Vicenin-II | *C. sativus* | Leaves | Mukherjee, Nema, Maity, & Sarkar, 2013 |
| **183** | Apigenin 7-*O*-(6″-*O*-*p*-coumaroylglucoside) | *C. sativus* | Leaves | Mukherjee, Nema, Maity, & Sarkar, 2013 |
| **184** | Isovitexin 2″-*O*-(6‴-*O*-(*E*)-*p*-coumaroyl)-glucopyranoside | *C. sativus* | Leaves | Mukherjee, Nema, Maity, & Sarkar, 2013 |
|  |  | *C. melo* var. *reticulatus* | Leaves | Hosoya, Masuda, Ohba, & Kumazawa, 2024 |
| **185** | Isovitexin 2″-*O*-(6⁗-*O*-(*E*)-*p*-coumaroyl)-glucoside-4′-*O*-glucoside | *C. sativus* | Leaves | Mukherjee, Nema, Maity, & Sarkar, 2013 |
| **186** | Isovitexin 2″-*O*-(6⁗-*O*-(*E*)-feruloyl) glucoside-4′-*O*-glucoside | *C. sativus* | Leaves | Mukherjee, Nema, Maity, & Sarkar, 2013 |
| **187** | Vitexin | *C. sativus* | Leaves | Mukherjee, Nema, Maity, & Sarkar, 2013 |
| **188** | Cucumerin A | *C. sativus* | Leaves; Flowers | Mukherjee, Nema, Maity, & Sarkar, 2013; Olennikov, 2023 |
| **189** | Cucumerin B | *C. sativus* | Leaves; Flowers | Mukherjee, Nema, Maity, & Sarkar, 2013; Olennikov, 2023 |
| **190** | Meloside A; [Isovitexin 2″-*O*-*β*-glucopyranoside] | *C. sativus* | Leaves | Olennikov & Kashchenko, 2024b |
|  |  | *C. melo* var. *cantalupensis* | Leaves | Hosoya, Masuda, Ohba, & Kumazawa, 2024 |
|  |  | *C. melo* var. *reticulatus* | Leaves | Hosoya, Masuda, Ohba, & Kumazawa, 2024 |
|  |  | *C. metuliferus* | ND | Krauze-Baranowska & Cisowski, 2001 |
|  |  | *C. myriocarpus* | ND | Krauze-Baranowska & Cisowski, 2001 |
| **191** | Isovitexin 2″-*O*-rhamnoside | *C. sativus* | Leaves | Olennikov & Kashchenko, 2023c |
| **192** | Isovitexin 7,2″-di-*O*-glucoside | *C. sativus* | Leaves | Olennikov & Kashchenko, 2023c |
| **193** | Isovitexin 4′,2″-di-*O*-glucoside | *C. sativus* | Leaves | Olennikov & Kashchenko, 2024b |
| **194** | Cucumerin C | *C. sativus* | Leaves; Flowers | Olennikov, 2023; Olennikov & Kashchenko, 2023c |
| **195** | Cucumerin D | *C. sativus* | Leaves; Flowers | Olennikov, 2023; Olennikov & Kashchenko, 2023c |
| **196** | Isovitexin 2″-*O*-glucoside-6″-*O*-ferulate | *C. sativus* | Leaves | Olennikov & Kashchenko, 2023a |
| **197** | Isovitexin 2″-*O*-glucoside-6″-*O*-*p*-coumarate | *C. sativus* | Leaves | Olennikov & Kashchenko, 2024b |
| **198** | Isovitexin 2″-*O*-(6‴-*O*-feruloyl)-glucoside-6″-*O*-ferulate | *C. sativus* | Leaves | Olennikov & Kashchenko, 2023a |
| **199** | Meloside a; [Isovitexin 2″-*O*-(6‴-*O*-caffeoyl)-glucopyranoside] | *C. sativus* | Leaves | Olennikov & Kashchenko, 2024b |
|  |  | *C. melo* var. *reticulatus* | Leaves | Hosoya, Masuda, Ohba, & Kumazawa, 2024 |
| **200** | Cucumoside A | *C. sativus* | Leaves | Olennikov & Kashchenko, 2024a |
| **201** | Cucumoside B | *C. sativus* | Leaves | Olennikov & Kashchenko, 2024a |
| **202** | Cucumoside C | *C. sativus* | Leaves | Olennikov & Kashchenko, 2024a |
| **203** | Cucumoside D | *C. sativus* | Leaves | Olennikov & Kashchenko, 2024a |
| **204** | Cucumoside E | *C. sativus* | Leaves | Olennikov & Kashchenko, 2024a |
| **205** | Cucumoside F | *C. sativus* | Leaves | Olennikov & Kashchenko, 2024a |
| **206** | Cucumoside G | *C. sativus* | Leaves | Olennikov & Kashchenko, 2024a |
| **207** | Cucumoside H | *C. sativus* | Leaves | Olennikov & Kashchenko, 2024b |
| **208** | Cucumoside I | *C. sativus* | Leaves | Olennikov & Kashchenko, 2024b |
| **209** | Cucumoside J | *C. sativus* | Leaves | Olennikov & Kashchenko, 2024b |
| **210** | Cucumoside K | *C. sativus* | Leaves | Olennikov & Kashchenko, 2024b |
| **211** | Isovitexin 4′-X-*O*-diglucoside | *C. sativus* | Leaves | Krauze-Baranowska & Cisowski, 2001 |
| **212** | Isovitexin 4′-*O*-glucoside | *C. sativus* | Leaves | Olennikov & Kashchenko, 2023b |
| **213** | Isovitexin 2″-*O*-*p*-coumarate | *C. sativus* | Leaves | Olennikov & Kashchenko, 2023b |
| **214** | Isovitexin 6″-*O*-*p*-coumarate | *C. sativus* | Leaves | Olennikov & Kashchenko, 2023b |
| **215** | Isovitexin 7-*O*-(6″-*O*-glucosyl)-glucoside | *C. sativus* | Leaves | Olennikov & Kashchenko, 2023b |
| **216** | Saponarin 6″-*O*-ferulate | *C. sativus* | Leaves; Flowers | Olennikov, 2023; Olennikov & Kashchenko, 2023b |
| **217** | Isovitexin 2″-*O*-(6‴-*O*-sinapoyl)-glucopyranoside | *C. melo* var. *reticulatus* | Leaves | Hosoya, Masuda, Ohba, & Kumazawa, 2024 |
| **218** | Orientin | *C. sativus* | Leaves; Flowers | Mukherjee, Nema, Maity, & Sarkar, 2013; Olennikov, 2023 |
| **219** | Isoorientin | *C. sativus* | Leaves; Flowers | Mukherjee, Nema, Maity, & Sarkar, 2013; Olennikov, 2023 |
|  |  | *C. myriocarpus* | ND | Krauze-Baranowska & Cisowski, 2001 |
| **220** | Swertiajaponin 4′-X-*O*-diglucoside | *C. sativus* | Leaves | Krauze-Baranowska & Cisowski, 2001 |
| **221** | Meloside L; [Isoorientin 2″-*O*-glucopyranoside] | *C. sativus* | Leaves; Flowers | Olennikov, 2023 |
|  |  | *C. melo* var. *cantalupensis* | Leaves | Hosoya, Masuda, Ohba, & Kumazawa, 2024 |
|  |  | *C. melo* var. *reticulatus* | Leaves | Hosoya, Masuda, Ohba, & Kumazawa, 2024 |
| **222** | Lutonarin | *C. sativus* | Flowers | Olennikov, 2023 |
| **223** | Isoorientin 6″-*O*-glucoside | *C. sativus* | Flowers | Olennikov, 2023 |
| **224** | Isoorientin 4′-*O*-glucoside | *C. sativus* | Flowers | Olennikov, 2023 |
| **225** | Luteolin 6-*C*-arabinoside | *C. sativus* | Flowers | Olennikov, 2023 |
| **226** | Juncein | *C. sativus* | Flowers | Olennikov, 2023 |
| **227** | 5,7-Di-*O*-methyl luteolin-6-*C*-(3″-*O*-benzoyl)-*β-D*-xyloside | *C. sativus* | Flowers | Nigussie & Ashenef, 2020 |
| **228** | Meloside b; [Isoorientin 2″-*O*-(6‴-*O*-caffeoyl)-glucopyranoside] | *C. melo* var. *reticulatus* | Leaves | Hosoya, Masuda, Ohba, & Kumazawa, 2024 |
| **229** | Isoorientin 2″-*O*-(6‴-*O*-(*E*)-*p*-coumaroyl)-glucopyranoside | *C. melo* var. *reticulatus* | Leaves | Hosoya, Masuda, Ohba, & Kumazawa, 2024 |
| **230** | Luteolin | *C. melo* var. *reticulatus* | Seeds | Ibrahim et al., 2019 |
| **231** | Luteolin 7-*O*-*β-D*-glucopyranoside | *C. melo* | Fruit peels | Gómez-García, Campos, Aguilar, Madureira, & Pintado, 2020 |
| **232** | Isoscoparin 2″-*O*-(6‴-(*E*)-feruloyl) glucoside | *C. sativus* | Leaves | Mukherjee, Nema, Maity, & Sarkar, 2013 |
| **233** | Isoscoparin 2″-*O*-(6‴-(*E*)-*p*-coumaroyl) glucoside | *C. sativus* | Leaves | Mukherjee, Nema, Maity, & Sarkar, 2013 |
| **234** | Isoscoparin 2″-*O*-(6‴-(*E*)-feruloyl) glucoside-4′-*O*-glucoside | *C. sativus* | Leaves | Mukherjee, Nema, Maity, & Sarkar, 2013 |
| **235** | Isoscoparin 2″-*O*-glucoside | *C. sativus* | Flowers | Olennikov, 2023 |
| **236** | Isoscoparin 7-*O*-glucoside | *C. sativus* | Flowers | Olennikov, 2023 |
| **237** | Rhamnetin; [3,5,3′,4′-Tetrahydroxy-7-*O*-methoxyflavone] | *C. sativus* | Leaves | Mukherjee, Nema, Maity, & Sarkar, 2013 |
| **238** | Kaempferol | *C. sativus* | Fruits | Ibitoye, Uwazie, & Ajiboye, 2018 |
| **239** | Kaempferol 3-*O*-rhamnoside | *C. sativus* | Flowers | Mukherjee, Nema, Maity, & Sarkar, 2013 |
| **240** | Kaempferol 3-*O*-glucoside | *C. sativus* | Flowers | Mukherjee, Nema, Maity, & Sarkar, 2013 |
| **241** | Isorhamnetin 3-*O*-glucoside | *C. sativus* | Flowers | Mukherjee, Nema, Maity, & Sarkar, 2013 |
| **242** | Isoquercitrin; [Quercetin 3-*O*-glucoside] | *C. sativus* | Flowers | Mukherjee, Nema, Maity, & Sarkar, 2013 |
|  |  | *C. melo* var. *reticulatus* | Seeds | Ibrahim et al., 2019 |
| **243** | Quercetin | *C. sativus* | Fruit peels | Anjani, Srivastava, & Mathur, 2023 |
|  |  | *C. melo* var. *reticulatus* | Seeds | Ibrahim et al., 2019 |
| **244** | Rutin; [Quercetin 3-*O*-*β-D*-glucopyranosyl-(1→6)-*α*-*L*-rhamnopyranoside] | *C. melo* var. *reticulatus* | Seeds | Ibrahim et al., 2019 |
| **245** | Quercetin 3-*O*-rutinoside-7-*O*-rhamnoside | *C. dipsaceus* | Fruits | Lata & Mittal, 2017a |
| **246** | (3*S*)-3,4′,5,6-Tetrahydroxy-7-methoxyhomoisoflavanone | *C. bisexualis* | Fruits | Ma, Wei, Sang, & Dong, 2021 |
| **247** | (3*S*)-(5,7-Dihydroxy-(3′-hydroxy-4′-methoxybenzyl)-4-chromanone | *C. bisexualis* | Fruits | Ma, Wei, Sang, & Dong, 2021 |
| **248** | (3*S*)-5,7-Dihydroxy-(4′-hydroxy-3′-methoxybenzyl)-4-chromanone | *C. bisexualis* | Fruits | Ma, Wei, Sang, & Dong, 2021 |
| **249** | (3*R*)-5,7-Dihydroxy-3-(4-methoxybenzyl)-8-methyl chroman-4-one | *C. bisexualis* | Fruits | Ma, Wei, Sang, & Dong, 2021 |
| **250** | 7-*O*-Methylpunctatin | *C. bisexualis* | Fruits | Ma, Wei, & Sang, 2020b |
| **251** | 7-*O*-Methyl-3′-hydroxypunctatin | *C. bisexualis* | Fruits | Ma, Wei, & Sang, 2020b |
| **252** | Punctatin | *C. bisexualis* | Fruits | Ma, Wei, & Sang, 2020b |
| **253** | Isointricatinol | *C. bisexualis* | Fruits | Ma, Wei, & Sang, 2020b |
| **254** | 8-Methoxybonducellin | *C. bisexualis* | Fruits | Ma, Wei, & Sang, 2020b |
| **255** | 3-(4′-Methoxybenzylidene)-5,7-dihydroxy-6-methoxychroman-4-one | *C. bisexualis* | Fruits | Ma, Wei, & Sang, 2020b |
| **256** | 3-(4′-Hydroxybenzylidene)-8-(3″,3″-dimethyl-furan-2″-one)-6,7-dimethoxy-chroman-4-one | *C. bisexualis* | Fruits | Ma, Wei, & Sang, 2020b |
| **257** | 3-(3′-Methoxy-4′-hydroxybenzylidene)-8-(3″,3″-dimethyl-furan-2″-one)-7-methoxy-chroman-4-one | *C. bisexualis* | Fruits | Ma, Wei, & Sang, 2020b |
| **258** | 3-(Benzo-dioxol-10-ylmethylene)-8-(3″,3″-dimethyl-furan-2″-one)-6-hydroxy-chroman-4-one | *C. bisexualis* | Fruits | Ma, Wei, & Sang, 2020b |
| **259** | 3-(Benzo-dioxol-10-ylmethylene)-8-(3″,3″-dimethyl-furan-2″-one)-6-hydroxy-5,7-dimethoxy-chroman-4-one | *C. bisexualis* | Fruits | Ma, Wei, & Sang, 2020b |
| **260** | (3*E*)-3-(1,3-Benzodioxol-5-ylmethylene)-2,3-dihydro-7-hydroxy-4*H*-1-benzopyran-4-one | *C. bisexualis* | Fruits | Ma, Wei, & Sang, 2020b |
| **261** | (3*E*)-3-(1,3-Benzodioxol-5-ylmethylene)-2,3-dihydro-7-methoxy-4*H*-1-benzopyran-4-one | *C. bisexualis* | Fruits | Ma, Wei, & Sang, 2020b |
| **262** | 3-(4′-Hydroxybenzylidene)-5-hydroxy-8-(6″,7″-dimethyl-furan-2″-one)-7‴,8‴-(10‴-isopropanol-furan)-chromene-1‴,4-dione | *C. bisexualis* | Fruits | Ma, Wei, Sang, & Dong, 2021 |
| **263** | 3-(3′,5′-Dimethoxy-4′-hydroxybenzylidene)-5-hydroxy-8-prenyl-7‴,8‴-isopropylpentenone-chromene-1‴,4-dione | *C. bisexualis* | Fruits | Ma, Wei, Sang, & Dong, 2021 |
| **264** | 3-(5′-Methoxy-4′-hydroxybenzylidene)-5-hydroxy-8-(4″,5″-dimethylbutan-1″-one)-6‴,7‴-(12‴,13‴-dimethyl-furan-9‴-one)-chromene-1‴,4-dione | *C. bisexualis* | Fruits | Ma, Wei, Sang, & Dong, 2021 |
| **265** | 3-(3′,5′-dimethoxy-4′-hydroxybenzylidene)-5-hydroxy-8-(4″,5″-dimethylallyl)-6‴,7‴-dioxolane-chromene-1‴,4-dione | *C. bisexualis* | Fruits | Ma, Wei, Sang, & Dong, 2021 |
| Coumarins and other phenolic compounds (94 compounds) | | | | |
| **266** | Murpanitin B | *C. bisexualis* | Fruits | Ma, Wei, Sang, & Dong, 2021 |
| **267** | Murpanitin C | *C. bisexualis* | Fruits | Ma, Wei, Sang, & Dong, 2021 |
| **268** | Murpanitin D | *C. bisexualis* | Fruits | Ma, Wei, Sang, & Dong, 2021 |
| **269** | 7-Hydroxy-3-(4′,6′-dihydroxy-5′-isopropyl-3″,3″-dimethyl-2*H*-chromen)-6-prenyl-2*H*-chromen-2-one | *C. bisexualis* | Fruits | Ma et al., 2018 |
| **270** | 7-Hydroxy-3-(5′-prenyl-3″,3″-dimethyl-2*H*-chromen)-6-prenyl-2*H*-chromen-2-one | *C. bisexualis* | Fruits | Ma et al., 2018 |
| **271** | 3-(6′-Hydroxy-5′-prenyl-3″,3″-dimethyl-2*H*-chromen)-6-prenyl-2*H*-chromen-2-one | *C. bisexualis* | Fruits | Ma et al., 2018 |
| **272** | 3-(5′-Ethyl-3″,3″-dimethyl-2*H*-chromen)-6-prenyl-2*H*-chromen-2-one | *C. bisexualis* | Fruits | Ma et al., 2018 |
| **273** | 3-(4′,6′-Dihydroxy-5′-dimethylallyl-3″,3″-dimethyl-2*H*-chromen)-6-prenyl-2*H*-chromen-2-one | *C. bisexualis* | Fruits | Ma et al., 2018 |
| **274** | 3-[4′,6′-Dihydroxy-5′-(2-propenyl)-3″,3″-dimethyl-2*H*-chromen]-14,15-dimethyl-pyrano-chromen-2-one | *C. bisexualis* | Fruits | Ma et al., 2018 |
| **275** | 3-(6′-Dihydroxy-5′-isopropanol-3″,3″-dimethyl-2*H*-chromen)-14,15-dimethyl-pyrano-chromen-2-one | *C. bisexualis* | Fruits | Ma et al., 2018 |
| **276** | 3-(5′-Isopentenol-3″,3″-dimethyl-2*H*-chromen)-14,15-dimethyl-pyrano-chromen-2-one | *C. bisexualis* | Fruits | Ma et al., 2018 |
| **277** | 3-(4′,6′-Dihydroxy-5′-prenyl-3″,3″-dimethyl-2*H*-chromen)-14,15-dimethyl-pyrano-chromen-2-one | *C. bisexualis* | Fruits | Ma et al., 2018 |
| **278** | Xanthyletin | *C. bisexualis* | Fruits | Ma et al., 2018 |
| **279** | Xanthoxyletin | *C. bisexualis* | Fruits | Ma et al., 2018 |
| **280** | Clausarin | *C. bisexualis* | Fruits | Ma et al., 2018 |
| **281** | Nordentatin | *C. bisexualis* | Fruits | Ma et al., 2018 |
| **282** | 4-(1-Methylpropyl)-5,7-dihydroxy-8-(4-hydroxy-3-methylbutyryl)-6-(3-methylbut-2-enyl)chromen-2-one | *C. bisexualis* | Fruits | Ma et al., 2018 |
| **283** | 5,7-Di-hydroxy-8-(4-hydroxy-3-methylbutyryl)-6-(3-methylbut-2-enyl)-4-phenylchromen-2-one | *C. bisexualis* | Fruits | Ma et al., 2018 |
| **284** | Brasimarin A | *C. bisexualis* | Fruits | Ma et al., 2018 |
| **285** | Asphodelin A | *C. bisexualis* | Fruits | Ma et al., 2018 |
| **286** | Muralatin D | *C. bisexualis* | Fruits | Ma et al., 2018 |
| **287** | Mammea B/BB | *C. bisexualis* | Fruits | Ma et al., 2018 |
| **288** | Robustic acid | *C. bisexualis* | Fruits | Ma et al., 2018 |
| **289** | Marianin A | *C. bisexualis* | Fruits | Ma et al., 2018 |
| **290** | 5,7-Dihydroxy-2-[2-(4-hydroxyphenyl)ethyl]chromone | *C. melo* var. *reticulatus* | Seeds | Ibrahim, 2010 |
| **291** | 7-Glucosyloxy-5-hydroxy-2-[2-(4-hydroxyphenyl)ethyl]chromone | *C. melo* var. *reticulatus* | Seeds | Ibrahim, 2010 |
| **292** | 5,7-Dihydroxy-2-[2-(3,4-dihydroxyphenyl)ethyl]chromone | *C. melo* var. *reticulatus* | Seeds | Ibrahim, 2010 |
| **293** | 5,7-Dihydroxy-2-[2-(3-methoxy-4-hydroxyphenyl)ethyl]chromone | *C. melo* var. *reticulatus* | Seeds | Ibrahim, 2014 |
| **294** | Cucumin S; [(*R*)-5,7-Dihydroxy-2-[1-hydroxy-2-(4-hydroxy-3-methoxyphenyl)ethyl]chromone] | *C. melo* var. *reticulatus* | Seeds | Ibrahim & Mohamed, 2015 |
| **295** | 1,3-Dihydroxy-2-methylene-(1′-isopentyloxy)-6-methoxymethyl-8-(6″,7″,8″-trihydroxyphenylketonyl)-9,10-anthraquinone | *C. bisexualis* | Fruits | Ma & Wei, 2021b |
| **296** | 8-(4′-Hydroxy-3′,5′-dimethoxyphenyl)-12-hydroxy-7-(6″,7″-dimethylfuran-2″-one)-9,16-anthraquinone-furochromene-3,2″-dione | *C. bisexualis* | Fruits | Ma & Wei, 2021a |
| **297** | 1,3-Dihydroxy-5-methoxy-6-methoxymethyl-2-methyl-9,10-anthraquinone | *C. bisexualis* | Fruits | Ma & Wei, 2021b |
| **298** | 1,3-Dihydroxy-5-methoxy-2,6-bismethoxymethyl-9,10-anthraquinone | *C. bisexualis* | Fruits | Ma & Wei, 2021b |
| **299** | 3,6-Dihydroxy-1,2-dimethoxyanthraquinone | *C. bisexualis* | Fruits | Ma & Wei, 2021b |
| **300** | 1,6-Dihydroxy-2-hydroxymethyl-5-methoxyanthraquinone | *C. bisexualis* | Fruits | Ma & Wei, 2021b |
| **301** | Symploquinone B | *C. bisexualis* | Fruits | Ma & Wei, 2021b |
| **302** | Symploquinone C | *C. bisexualis* | Fruits | Ma & Wei, 2021b |
| **303** | 1-Hydroxy-2-((isopentyloxy)methyl)-3-methoxy-9,10-anthraquinone | *C. bisexualis* | Fruits | Ma & Wei, 2021b |
| **304** | 1-Methoxy-2′,2′-dimethyldioxine-(5′,6′:2,3)-anthraquinone | *C. bisexualis* | Fruits | Ma & Wei, 2021b |
| **305** | Averythrin | *C. bisexualis* | Fruits | Ma & Wei, 2021b |
| **306** | 5,8-Dihydroxy-7′-isopropyl-furan ring-1″-prenyl-naphthoquinone | *C. bisexualis* | Fruits | Ma, Wei, & Sang, 2020c |
| **307** | 5,8-Dihydroxy-7′-propenyl-furan ring-1″-isopropanol-naphthoquinone | *C. bisexualis* | Fruits | Ma, Wei, & Sang, 2020c |
| **308** | 5,8-Dihydroxy-7′-isopropanol-furan ring-1″-(5″-hydroxyphenyl)-naphthoquinone | *C. bisexualis* | Fruits | Ma, Wei, & Sang, 2020c |
| **309** | Shikometabolin G | *C. bisexualis* | Fruits | Ma, Wei, & Sang, 2020c |
| **310** | Naphthofuranin A | *C. bisexualis* | Fruits | Ma, Wei, & Sang, 2020c |
| **311** | (2*R*)-6-Hydroxy-7-methoxy-dehydroiso-*α*-lapachone | *C. bisexualis* | Fruits | Ma, Wei, & Sang, 2020c |
| **312** | 2-(1,4-Dioxo-1,4-dihydronaphthalen-2-yloxy)-3-hydroxynaphthalene-1,4-dione | *C. bisexualis* | Fruits | Ma, Wei, & Sang, 2020c |
| **313** | Zeylanone | *C. bisexualis* | Fruits | Ma, Wei, & Sang, 2020c |
| **314** | 1-Methyl-2,4-dimethoxy-3-hydroxyanthraquinone | *C. bisexualis* | Fruits | Ma, Wei, & Sang, 2020c |
| **315** | Javanicin | *C. bisexualis* | Fruits | Ma, Wei, & Sang, 2020c |
| **316** | Anhydrofusarubin | *C. bisexualis* | Fruits | Ma, Wei, & Sang, 2020c |
| **317** | Shikonin | *C. bisexualis* | Fruits | Ma, Wei, & Sang, 2020c |
| **318** | Lapachol | *C. bisexualis* | Fruits | Ma, Wei, & Sang, 2020c |
| **319** | Sulfuretin | *C. bisexualis* | Fruits | Ma, Wei, & Sang, 2020a |
| **320** | (*Z*)-4-Methoxy-6,4′-dihydroxyaurone | *C. bisexualis* | Fruits | Ma, Wei, & Sang, 2020a |
| **321** | Aureusidin | *C. bisexualis* | Fruits | Ma, Wei, & Sang, 2020a |
| **322** | Altilisin I | *C. bisexualis* | Fruits | Ma, Wei, & Sang, 2020a |
| **323** | Altilisin J | *C. bisexualis* | Fruits | Ma, Wei, & Sang, 2020a |
| **324** | (*Z*)-2-(3′-Methoxy-4′-hydroxybenzylidene)-5-hydroxy-6-(6″,7″-dimethyl-furan-2″-one)-furo-chromene-3,7-dione | *C. bisexualis* | Fruits | Ma, Wei, & Sang, 2020a |
| **325** | (*Z*)-2-(4′-Hydroxybenzylidene)-5-hydroxy-6-(4″,5″-dimethyl-1″-phenylbutanone)-9-prenyl-furochromene-3,7-dione | *C. bisexualis* | Fruits | Ma, Wei, & Sang, 2020a |
| **326** | (*Z*)-2-(3′,4′,5′-Trimethoxybenzylidene)-5-hydroxy-6-iso-propanol-furochromene-3,7-dione | *C. bisexualis* | Fruits | Ma, Wei, & Sang, 2020a |
| **327** | Astrernestin | *C. bisexualis* | Fruits | Ma & Wei, 2023 |
| **328** | Cotinignan A | *C. bisexualis* | Fruits | Ma & Wei, 2023 |
| **329** | 6-Hydroxy-7′-(4″-hydroxyphenyl)-10′-acetylauronolignan | *C. bisexualis* | Fruits | Ma & Wei, 2023 |
| **330** | (+)-(1*R*,2*S*,5*R*,6*S*)-2,6-Di-(4′-hydroxyphenyl)-3,7-dioxabicyclo(3,3,0)octane | *C. sativus* | Leaves | Mukherjee, Nema, Maity, & Sarkar, 2013 |
| **331** | Sisymbrifolin | *C. sativus* | Whole plant | Kato-Noguchi, Le Thi, Sasaki, & Suenaga, 2012 |
| **332** | Lariciresinol | *C. sativus*; *C. melo* | Fruits | Milder, Arts, van de Putte, Venema, & Hollman, 2005 |
| **333** | Pinoresinol | *C. sativus*; *C. melo* | Fruits | Milder, Arts, van de Putte, Venema, & Hollman, 2005 |
| **334** | Secoisolariciresinol | *C. sativus*; *C. melo* | Fruits | Milder, Arts, van de Putte, Venema, & Hollman, 2005 |
| **335** | 2-(3′-Methoxyphenyl)-(4′→1″)-*O*-*β-D*-glucopyranosyl-7‴-(4‴-methoxyphenyl)-pyrano-chromene-4,9‴-dione | *C. bisexualis* | Fruits | Ma, Liu, & Wei, 2024 |
| **336** | (7″S,8″S)-3′-Hydroxyl-4′,5″-dimethoxyl rhodiolin | *C. bisexualis* | Fruits | Ma, Liu, & Wei, 2024 |
| **337** | Stachyol C | *C. bisexualis* | Fruits | Ma, Liu, & Wei, 2024 |
| **338** | Sonyamandin | *C. bisexualis* | Fruits | Ma, Liu, & Wei, 2024 |
| **339** | Silybin A | *C. bisexualis* | Fruits | Ma, Liu, & Wei, 2024 |
| **340** | Silychristin A | *C. bisexualis* | Fruits | Ma, Liu, & Wei, 2024 |
| **341** | 6-(3′,4′-Dihydroxy-5-methyl-[1,1′-biphenyl]-3-yl)-2,5-dihydroxybenzofuran-7-yl 2,4-dihydroxy-3-(3-methylbut-2-en-1-yl)benzoate | *C. bisexualis* | Fruits | Ma, Liu, Sang, & Wei, 2025 |
| **342** | Tetralatabiphenyl B | *C. bisexualis* | Fruits | Ma, Liu, Sang, & Wei, 2025 |
| **343** | Tetralatabiphenyl C | *C. bisexualis* | Fruits | Ma, Liu, Sang, & Wei, 2025 |
| **344** | [1,1′-Biphenyl]-2-(3-methyl-2-butenyl)-3-methoxy-4,4′,5,6-tetraol | *C. bisexualis* | Fruits | Ma, Liu, Sang, & Wei, 2025 |
| **345** | Tababiphenyl A | *C. bisexualis* | Fruits | Ma, Liu, Sang, & Wei, 2025 |
| **346** | Tababiphenyl C | *C. bisexualis* | Fruits | Ma, Liu, Sang, & Wei, 2025 |
| **347** | Berbekorin A | *C. bisexualis* | Fruits | Ma, Liu, Sang, & Wei, 2025 |
| **348** | Fortuneanoside F | *C. bisexualis* | Fruits | Ma, Liu, Sang, & Wei, 2025 |
| **349** | Gallic acid | *C. sativus* | Leaves; Herbs | Gopalasatheeskumar et al., 2020; Ibrahim, El-Hefnawy, & El-Hela, 2010 |
| **350** | Syringic acid | *C. sativus* | Herbs | Ibrahim, El-Hefnawy, & El-Hela, 2010 |
| **351** | *p*-Coumaric acid | *C. sativus* | Leaves; Roots; Herbs | Ibrahim, El-Hefnawy, & El-Hela, 2010; Mukherjee, Nema, Maity, & Sarkar, 2013; Zhou et al., 2012 |
| **352** | *p*-Coumaric acid methyl ester | *C. sativus* | Leaves | McNally, Wurms, Labbé, & Bélanger, 2003 |
| **353** | Ferulic acid | *C. sativus* | Leaves; Herbs | Ibrahim, El-Hefnawy, & El-Hela, 2010; McNally, Wurms, Labbé, & Bélanger, 2003 |
| **354** | Caffeic acid | *C. sativus* | Leaves; Herbs | Ibrahim, El-Hefnawy, & El-Hela, 2010; McNally, Wurms, Labbé, & Bélanger, 2003 |
| **355** | Sinapic acid | *C. sativus* | Leaves | McNally, Wurms, Labbé, & Bélanger, 2003 |
| **356** | Salicylic acid | *C. sativus* | Leaves | Segarra, Jáuregui, Casanova, & Trillas, 2006 |
| **357** | Gentisic acid 5-*O*-*β-D*-xylopyranoside | *C. sativus* | Leaves | Fayos, Bellés, López-Gresa, Primo, & Conejero, 2006 |
| **358** | Chlorogenic acid | *C. sativus* | Herbs | Ibrahim, El-Hefnawy, & El-Hela, 2010 |
| **359** | (*E*)-4-Hydroxycinnamyl alcohol 4-*O*-(2′-*O*-*β-D*-apiofuranosyl)(1″→2′)-*β-D*-glucopyranoside | *C. melo* var. *inodorus* | Seeds | De Marino, Festa, Zollo, & Iorizzi, 2009 |
| Other compounds (69 compounds) | | | | |
| **360** | CMPP-1 | *C. metuliferus* | Fruit peels | Zhu et al., 2021 |
| **361** | CMPP-2 | *C. metuliferus* | Fruit peels | Zhu et al., 2021 |
| **362** | 7*α*-Hydroxyorobanchyl acetate | *C. sativus* | Root exudates | Khetkam et al., 2014 |
| **363** | 7*α*-Hydroxyorobanchol | *C. sativus* | Root exudates | Khetkam et al., 2014 |
| **364** | 7*β*-Hydroxyorobanchyl acetate | *C. sativus* | Root exudates | Khetkam et al., 2014 |
| **365** | 7*β*-Hydroxyorobanchol | *C. sativus* | Root exudates | Khetkam et al., 2014 |
| **366** | 7-Oxoorobanchyl acetate | *C. sativus* | Root exudates | Khetkam et al., 2014 |
| **367** | 7-Oxoorobanchol | *C. sativus* | Root exudates | Khetkam et al., 2014 |
| **368** | Orobanchyl acetate | *C. sativus* | Root exudates | Khetkam et al., 2014 |
| **369** | Orobanchol | *C. sativus* | Root exudates | Khetkam et al., 2014 |
| **370** | 4-Deoxyorobanchol | *C. sativus* | Root exudates | Khetkam et al., 2014 |
| **371** | Cucumegastigmane I | *C. sativus* | Leaves | Mukherjee, Nema, Maity, & Sarkar, 2013 |
|  |  | *C. melo* var. *reticulatus* | Leaves | Hosoya, Masuda, Ohba, & Kumazawa, 2024 |
| **372** | Cucumegastigmane II | *C. sativus* | Leaves | Mukherjee, Nema, Maity, & Sarkar, 2013 |
|  |  | *C. melo* var. *reticulatus* | Leaves | Hosoya, Masuda, Ohba, & Kumazawa, 2024 |
| **373** | 9-Hydroxy-4,7-megastigmadien-9-one | *C. sativus* | Stems; Leaves; Roots | Kato-Noguchi, Le Thi, Sasaki, & Suenaga, 2012 |
| **374** | (+)-Dehydrovomifoliol | *C. sativus* | Leaves | Mukherjee, Nema, Maity, & Sarkar, 2013 |
| **375** | (6*S*,9*S*)-6-Hydroxyl-3-oxo-*α*-ionol-9-*O*-*β-D*-glucopyranoside | *C. melo* | Pedicels | Chen et al., 2014 |
| **376** | Staphylionoside D | *C. melo* var. *reticulatus* | Leaves | Hosoya, Masuda, Ohba, & Kumazawa, 2024 |
| **377** | Gibberellin A_1_ | *C. sativus* | Seeds; Stems; Leaves | Hemphill Jr, Baker, & Sell, 1972; Smith, Sponsel, Knatt, Gaskin, & MacMillan, 1991 |
|  |  | *C. melo* | Seeds | Hemphill Jr, Baker, & Sell, 1972 |
| **378** | Gibberellin A_3_ | *C. sativus*; *C. melo* | Seeds | Hemphill Jr, Baker, & Sell, 1972 |
| **379** | Gibberellin A_4_ | *C. sativus* | Seeds; Stems; Leaves | Hemphill Jr, Baker, & Sell, 1972; Smith, Sponsel, Knatt, Gaskin, & MacMillan, 1991 |
| **380** | Gibberellin A_7_ | *C. sativus* | Seeds | Hemphill Jr, Baker, & Sell, 1972 |
| **381** | Gibberellin A_1_-*β-D*-glucoside | *C. sativus* | Seeds | Hemphill Jr, Baker, & Sell, 1973 |
| **382** | Gibberellin A_1_-*n*-propyl ester | *C. sativus* | Seeds | Hemphill Jr, Baker, & Sell, 1973 |
| **383** | Gibberellin A_3_-*n*-propyl ester | *C. sativus* | Seeds | Hemphill Jr, Baker, & Sell, 1973 |
| **384** | Gibberellin A_5_ | *C. sativus* | Stems; Leaves | Smith, Sponsel, Knatt, Gaskin, & MacMillan, 1991 |
|  |  | *C. melo* | Seeds | Hemphill Jr, Baker, & Sell, 1972 |
| **385** | Gibberellin A_34_ | *C. sativus* | Stems; Leaves | Smith, Sponsel, Knatt, Gaskin, & MacMillan, 1991 |
| **386** | Gibberellin A_20_ | *C. sativus* | Stems; Leaves | Smith, Sponsel, Knatt, Gaskin, & MacMillan, 1991 |
| **387** | Methyl-1*H*-indole-3-acetate | *C. sativus* | Roots | Zhou et al., 2012 |
| **388** | 1*H*-Indole-3-aldehyde | *C. sativus* | Roots; Leaves | Mukherjee, Nema, Maity, & Sarkar, 2013; Zhou et al., 2012 |
| **389** | Methyl-1*H*-indole-3-carboxylate | *C. sativus* | Roots | Zhou et al., 2012 |
| **390** | Indole-3-carboxylic acid | *C. sativus* | Leaves | Mukherjee, Nema, Maity, & Sarkar, 2013 |
| **391** | Tryptophol | *C. sativus* | Shoots | Rayle & Purves, 1967 |
| **392** | Riboflavin | *C. sativus* | ND | Satoh et al., 2016 |
| **393** | 4′-Ketoriboflavin | *C. sativus* | ND | Satoh et al., 2016 |
| **394** | *β*-Pyrazol-1-yl-*L*-alanine | *C. sativus*; *C. melo*; *C. ficifolius* | Seeds | Dunnill & Fowden, 1965 |
| **395** | *γ*-*L*-Glutamyl-*β*-pyrazol-1-yl-*L*-alanine | *C. sativus*; *C. melo*; *C. ficifolius* | Seeds | Dunnill & Fowden, 1965 |
| **396** | Citrulline | *C. sativus*; *C. melo*; *C. ficifolius* | Seeds | Dunnill & Fowden, 1965 |
| **397** | IdoBR1 | *C. sativus* | Fruits | Nash et al., 2020 |
| **398** | N-Trisaccharide | *C. prophetarum* | Fruits | Kavishankar & Lakshmidevi, 2014 |
| **399** | (2*S*,3*S*,4*R*,10*E*)-2-[(2′*R*)-2-Hydroxytetracosanoylamino]-1,3,4-octadecanetriol-10-ene | *C. sativus* | Stems | Tang et al., 2010 |
| **400** | 1-*O*-*β-D*-Glucopyranosyl-(2*S*,3*S*,4*R*,10*E*)-2-[(2′*R*)-2-hydroxytetracosanoylamino]-1,3,4-octadecane-triol-10-ene | *C. sativus* | Stems | Tang et al., 2010 |
| **401** | Soya-cerebroside I | *C. sativus* | Stems | Tang et al., 2010 |
| **402** | (2*S*,3*S*,4*R*,10*E*)-2-(2′,3′-Dihydroxytetracosanoylamino)-10-octadecene-1,3,4-triol | *C. sativus* | Stems | Tang et al., 2010 |
| **403** | *L*-(+)-Lactic acid | *C. sativus* | Fruits | Mukherjee, Nema, Maity, & Sarkar, 2013 |
| **404** | *t*-Cinnamic acid | *C. sativus* | Leaves | McNally, Wurms, Labbé, & Bélanger, 2003 |
| **405** | Lutein | *C. sativus* | Leaves | Mukherjee, Nema, Maity, & Sarkar, 2013 |
| **406** | Benzyl *O*-*β-D*-glucopyranoside | *C. melo* var. *inodorus* | Seeds | De Marino, Festa, Zollo, & Iorizzi, 2009 |
| **407** | Ethyl *O*-*β-D*-glucopyranoside | *C. melo* | Pedicels | Chen et al., 2014 |
| **408** | *p*-Hydroxybenzaldehyde | *C. sativus* | Roots | Zhou et al., 2012 |
| **409** | Isovanillin | *C. sativus* | Roots | Zhou et al., 2012 |
| **410** | 2-Hydroxybenzyl alcohol | *C. sativus* | Roots | Zhou et al., 2012 |
| **411** | 1,3-Di-(6*Z*,9*Z*)-docosa-6,9-dienoyl-2-(6*Z*) hexacos-6-enoylglycerol | *C. melo* var. *reticulatus* | Seeds | Ibrahim, 2014 |
| **412** | (*S*)-2-Benzoyloxy-3-phenyl-1-propanol | *C. sativus* | ND | Kato-Noguchi, Le Thi, Sasaki, & Suenaga, 2012 |
| **413** | 2,3-Dihydro-3,5-dihydroxy-6-methyl-4*H*-pyran-4-one | *C. sativus* | Fruits | Idemudia & Enogieru, 2024 |
| **414** | 5-(Hydroxylmethyl)-2-furancarboxaldehyde | *C. sativus* | Fruits | Idemudia & Enogieru, 2024 |
| **415** | 4-Hydroxy-3-methyl-2-butenyl-acetate | *C. sativus* | Fruits | Idemudia & Enogieru, 2024 |
| **416** | 1,2-Benzenedicarboxylic acid-diisooctylester | *C. sativus* | Fruits | Idemudia & Enogieru, 2024 |
| **417** | Galactinol | *C. sativus* | Leaves | Pharr, Hendrix, Robbins, Gross, & Sox, 1987 |
| **418** | Jasmonic acid | *C. sativus* | ND | Segarra, Jáuregui, Casanova, & Trillas, 2006 |
| **419** | (9*Z*,12*Z*,14*E*,16*S*)-16-Hydroxy-9,12,14-octadecatrienoic acid methyl ester | *C. sativus* | Leaves; Fruit peels; Flowers | Gorina, Egorova, Lantsova, Toporkova, & Grechkin, 2023 |
| **420** | (9*Z*,12*Z*,14*E*,16*S*)-16-Hydroperoxy-9,12,14-octadecatrienoic acid methyl ester | *C. sativus* | Leaves; Fruit peels; Flowers | Gorina, Egorova, Lantsova, Toporkova, & Grechkin, 2023 |
| **421** | *cis*-8-Pentadecenal | *C. sativus* | Fruits | Kemp, 1977 |
| **422** | 8,16-Dihydroxyhexadecanoic acid | *C. sativus* | Fruits | Gérard, Pfeffer, & Osman, 1994 |
| **423** | Myristic acid | *C. prophetarum* | Roots | Galma et al., 2021 |
|  |  | *C. ficifolius* | Fruits | Ayele, Gurmessa, Abdissa, & Abdissa, 2021 |
| **424** | Pentadecanoic acid | *C. ficifolius* | Fruits | Ayele, Gurmessa, Abdissa, & Abdissa, 2021 |
| **425** | 2′,3′-Dihydroxypropyl pentadecanoate | *C. ficifolius* | Fruits | Ayele, Gurmessa, Abdissa, & Abdissa, 2021 |
| **426** | Propylicosa-9,12-dienoate | *C. dipsaceus* | Fruits | Assefa et al., 2024 |
| **427** | Hexacosane | *C. dipsaceus* | Fruits | Assefa et al., 2024 |
| **428** | Octadecane | *C. dipsaceus* | Fruits | Assefa et al., 2024 |
| **^(*)^**, Additional sources of cucurbitacins (depicted in **Table S2**) adapted from reference (Rehm, Enslin, Meeuse, & Wessels, 1957).  Note: The words between “square brackets [ ]” refer to the other names of metabolites mentioned in the references cited; “ND”, The specific organ was not determined (more likely to be fruits). | | | | |

**Table S2** High content of bitter principles “cucurbitacins” in selected Cucumis species.

| *Cucumis* species | Author | Plant part used | Cucurbitacins^(a)^ | | | | | | | |
| --- | --- | --- | --- | --- | --- | --- | --- | --- | --- | --- |
|  |  |  | A (**100**) | B (**91**) | C (**77**) | D (**108**) | E (**95**) | F (**94**) | G (**110**) | H (**111**) |
| *C. africanus* | L.f. | Fruits | - | ** | - | traces | - | - | - | - |
| *C. anguria* | L. | Fruits | - | ND | - | - | - | - | - | - |
| *C. angolensis* | Hook.f. | Fruits | - | * | - | *** | - | traces | * | * |
|  |  | Leaves | - | * | - | ** | - | ** | - | - |
|  |  | Roots | - | * | - | ** | - | * | * | * |
| *C. dinteri* | Cogn. | Fruits | - | * | - | *** | - | traces | * | * |
|  |  | Leaves | - | * | - | ** | - | ** | - | - |
|  |  | Roots | - | * | - | ** | - | * | * | * |
| *C. dipsaceus* | Ehrenb. ex Spach | Fruits | - | ** | - | * | - | - | - | - |
| *C. heptadactylus* | Naudin | Fruits | - | ** | - | * | - | - | - | - |
|  |  | Roots | - | ** | - | ** | - | - | ** | ** |
| *C. hirsutus* | Sond. | Roots | - | * | - | ** | - | - | ** | ** |
| *C. hookeri* | Naudin | Fruits | ** | ** | - | ** | - | - | traces | traces |
| *C. humofructus* | Stent | Fruits | - | * | - | traces | - | - | - | - |
| *C. myriocarpus* | Naudin | Fruits | *** | * | - | * | - | - | - | - |
| *C. leptodermis* | Schweick. | Fruits | *** | trace | - | traces | - | - | - | - |
| *C. ficifolius* | A.Rich. | Fruits | - | *** | - | * | - | - | traces | traces |
|  |  | Roots^(b)^ | - | *** | - | *** | - | - | - | - |
| *C. longipes* | Hook.f. | Fruits | - | *** | - | * | - | - | traces | traces |
| *C. metuliferus* | E.Mey. ex Naudin | Fruits | - | traces | - |  | - | - | - | - |
| *C. pustulatus* | Hook.f. | Fruits | - | ** | - | * | - | - | - | - |
| *C. kalahariensis* | A.Meeuse | Fruits | - | ND | - | ND | - | - | - | - |
| *C. zeyheri* | Sond. | Fruits^(c)^ | - | ND | - | ND | - | - | - | - |
| Note: Data were adapted from references ^(a)^: (Miró, 1995; Rehm, Enslin, Meeuse, & Wessels, 1957); ^(b)^: (Nigussie & Ashenef, 2020), ^(c)^:([https://tropical.theferns.info](https://tropical.theferns.info/)); ND: not determined; “***”: the amount of bitter principle = 0.1% or higher; “**”: the amount of bitter principle = 0.01% to 0.09%, “*”: the amount of bitter principle = 0.001% to 0.009%, “traces”: the bitter principle is < 0.001%; “-”: absent; “ ”: the bitter principle has been isolated and identified from the corresponding plant. | | | | | | | | | | |

# **References**

Abd El-Fattah, H., Zaghloul, A. M., Halim, A. F., & Waight, E. S. (1989). Cucurbitacins and steroids from *Cucumis callosus* (Rottl) Cong. *Acta Pharmaceutica*, *39*(2), 137−141.

Afifi, M. S., Ross, S. A., Elsohly, M. A., Naeem, Z. E., & Halaweish, F. T. (1999). Cucurbitacins of *Cucumis prophetarum* and *Cucumis prophetarum*. *Journal of Chemical Ecology*, *25*(4), 847−859.

Akihisa (ne Itoh), T., Ghosh, P., Thakur, S., Rosentein, F. U., & Matsumoto, T. (1986). Sterol compositions of seeds and mature plants of family Cucurbitaceae. *Journal of the American Oil Chemists’ Society*, *63*(5), 653−658.

Akihisa, T., Inada, Y., Ghosh, P., Thakur, S., Rosenstein, F. U., Tamura, T., et al. (1988). Compositions of triterpene alcohols of seeds and mature plants of family Cucurbitaceae. *Journal of the American Oil Chemists’ Society*, *65*(4), 607−610.

Akihisa, T., Kimura, Y., Kasahara, Y., Kumaki, K., Thakur, S., & Tamura, T. (1997). 7-Oxodihydrokarounidol-3-benzoate and other triterpenes from the seeds of Cucurbitaceae. *Phytochemistry*, *46*(7), 1261−1266.

Akihisa, T., Shimizu, N., Tamura, T., & Matsumoto, T. (1986). (24*R*‐14*α*‐Methyl‐24‐ethyl‐5*α*‐cholest‐9(11)‐en‐3*β*‐ol: A new 14*α*‐methylsterol from *Cucumis sativus*. *Lipids*, *21*(8), 491−493.

Akihisa, T., Thakur, S., Rosenstein, F. U., & Matsumoto, T. (1986). Sterols of Cucurbitaceae: The configurations at C‐24 of 24‐alkyl‐Δ^5^‐, Δ^7^‐ and Δ^8^‐ sterols. *Lipids*, *21*(1), 39−47.

Akiyama, K., & Hayashi, H. (2002). Arbuscular mycorrhizal fungus-promoted accumulation of two new triterpenoids in cucumber roots. *Bioscience, Biotechnology, and Biochemistry*, *66*(4), 762−769.

Akjhisa, T., Shimizu, N., Ghosh, P., Thakur, S., Rosenstein, F. U., Tamura, T., et al. (1987). Sterols of the Cucurbitaceae. *Phytochemistry*, *26*(6), 1693−1700.

Aljohani, O. S. (2022). Phytochemical evaluation of *Cucumis prophetarum*: Protective effects against carrageenan-induced prostatitis in rats. *Drug and Chemical Toxicology*, *45*(4), 1461−1469.

Al-Rehaily, A. J., Al-Yahya, M. A., Mirza, H. H., & Ahmed, B. (2002). Cucumidisecosterol: A new diseco-sterol from *Cucumis prophetarum*. *Pharmaceutical Biology*, *40*(2), 154−159.

Alsayari, A., Kopel, L., Ahmed, M. S., Soliman, H. S. M., Annadurai, S., & Halaweish, F. T. (2018). Isolation of anticancer constituents from *Cucumis prophetarum* var. *prophetarum* through bioassay-guided fractionation. *BMC Complementary and Alternative Medicine*, *18*(1), 274.

Anjani, Srivastava, N., & Mathur, J. (2023). Isolation, purification and characterization of quercetin from *Cucumis sativus* peels; its antimicrobial, antioxidant and cytotoxicity evaluations. *3 Biotech*, *13*(2), 46.

Appendino, G., Jakupovic, J., Belloro, E., & Marchesini, A. (1999). Multiflorane triterpenoid esters from pumpkin. An unexpected extrafolic source of PABA. *Phytochemistry*, *51*(8), 1021−1026.

Assefa, T., Tesso, H., Ramachandran, V. P., Guta, L., Demissie, T. B., Ombito, J. O., et al. (2024). *In silico* molecular docking analysis, cytotoxicity, and antibacterial activities of constituents of fruits of *Cucumis dipsaceus*. *ACS Omega*, *9*(1), 1945−1955.

Atta-ur-Rahman, Ahmed, V. U., Khan, M. A., & Zehra, F. (1973). Isolation and structure of cucurbitacin Q_1_. *Phytochemistry*, *12*(11), 2741−2743.

Ayele, T. T., Gurmessa, G. T., Abdissa, Z., & Abdissa, N. (2021). Chemical constituents of the fruits of *Cucumis ficifolius* and evaluation for antibacterial activity. *Organic Chemistry: Current Research*, *10*(8), 1000P483.

Busuioc, A. C., Costea, G. V., Botezatu, A. V. D., Furdui, B., & Dinica, R. M. (2023). *Cucumis metuliferus* L. fruits extract with antioxidant, anti-inflammatory, and antidiabetic properties as source of ursolic acid. *Separations*, *10*(5), 274.

Chen, C., Qiang, S. G., Lou, L. G., & Zhao, W. M. (2009). Cucurbitane-type triterpenoids from the stems of *Cucumis melo*. *Journal of Natural Products*, *72*(5), 824−829.

Chen, S. Y., Zhou, Q. Y., Chen, L., Li, J. Y., Xie, T., & Zhang, S. H. (2022). Screening and identifying cucurbitacins and cucurbitacin glycosides in *Cucumis sativus* using high-performance liquid chromatography/quadrupole-time-of-flight mass spectrometry combined with in-source fragmentation and alkali adduct ions. *Rapid Communications in Mass Spectrometry*, *36*(14), e9323.

Chen, W. J., Sun, Y., Yuan, Z. K., Li, Y. J., Wang, L. B., & Wu, L. J. (2014). Isolation and identification of the chemical constituents of *Pedicellus Melo*. *Journal of Shenyang Pharmaceutical University*, *31*, 351−354.

De Marino, S., Festa, C., Zollo, F., & Iorizzi, M. (2009). Phenolic glycosides from *Cucumis melo* var. *inodorus* seeds. *Phytochemistry Letters*, *2*(3), 130−133.

Deepika, Kumari, A., Prajapati, P., Sarita, Kumar, S., Aluko, R. E., et al. (2023). Pharmacological and therapeutic potential of *Cucumis callosus*: A novel nutritional powerhouse for the management of non-communicable diseases. *Plant Foods for Human Nutrition*, *78*(4), 630−642.

Du, Q. Z., Xiong, X. P., & Ito, Y. (1995). Separation of cucurbitacin B and cucurbitacin E from fruit base of *Cucumis melo* L. by high-speed countercurrent chromatography. *Modern Countercurrent Chromatography*. American Chemical Society, pp 107−110.

Dunnill, P. M., & Fowden, L. (1965). The amino acids of seeds of the Cucurbitaceae. *Phytochemistry*, *4*(6), 933−944.

Fayos, J., Bellés, J. M., López-Gresa, M. P., Primo, J., & Conejero, V. (2006). Induction of gentisic acid 5-*O*-*β-D*-xylopyranoside in tomato and cucumber plants infected by different pathogens. *Phytochemistry*, *67*(2), 142−148.

Galma, W., Endale, M., Getaneh, E., Eswaramoorthy, R., Assefa, T., & Melaku, Y. (2021). Antibacterial and antioxidant activities of extracts and isolated compounds from the roots extract of *Cucumis prophetarum* and in silico study on DNA gyrase and human peroxiredoxin 5. *BMC Chemistry*, *15*(1), 32.

Garg, V. K., & Nes, W. R. (1986). Occurrence of Δ^5^-sterols in plants producing predominantly Δ^7^-sterols: Studies on the sterol compositions of six Cucurbitaceae seeds. *Phytochemistry*, *25*(11), 2591−2597.

Gérard, H. C., Pfeffer, P. E., & Osman, S. F. (1994). 8,16-Dihydroxyhexadecanoic acid, a major component from cucumber cutin. *Phytochemistry*, *35*(3), 818−819.

Gómez-García, R., Campos, D. A., Aguilar, C. N., Madureira, A. R., & Pintado, M. (2020). Valorization of melon fruit (*Cucumis melo* L.) by-products: Phytochemical and biofunctional properties with emphasis on recent trends and advances. *Trends in Food Science & Technology*, *99*, 507−519.

Gopalasatheeskumar, K., Ariharasivakumar, G., Kalaichelvan, V. K., Sengottuvel, T., Devan, V. S., & Srividhya, V. (2020). Antihyperglycemic and antihyperlipidemic activities of wild musk melon (*Cucumis melo* var. *agrestis*) in streptozotocin-nicotinamide induced diabetic rats. *Chinese Herbal Medicines*, *12*(4), 399−405.

Gorina, S. S., Egorova, A. M., Lantsova, N. V., Toporkova, Y. Y., & Grechkin, A. N. (2023). Discovery of *α*-linolenic acid 16(*S*)-lipoxygenase: Cucumber (*Cucumis sativus* L.) vegetative lipoxygenase 3. *International Journal of Molecular Sciences*, *24*(16), 12977.

Hemphill Jr, D. D., Baker, L. R., & Sell, H. M. (1972). Isolation and identification of the gibberellins of *Cucumis sativus* and *Cucumis melo*. *Planta*, *103*(3), 241−248.

Hemphill Jr, D. D., Baker, L. R., & Sell, H. M. (1973). Isolation of novel conjugated gibberellins from *Cucumis sativus* seed. *Canadian Journal of Biochemistry*, *51*(12), 1647−1653.

Hosoya, T., Masuda, Y., Ohba, S., & Kumazawa, S. (2024). Component analysis of *Cucumis melo* L. leaves and their antioxidant activity. *Natural Product Research*, 1−8.

Ibitoye, O. B., Uwazie, J. N., & Ajiboye, T. O. (2018). Bioactivity-guided isolation of kaempferol as the antidiabetic principle from *Cucumis sativus* L. fruits. *Journal of Food Biochemistry*, *42*(4), e12479.

Ibrahim, S. R. M. (2010). New 2-(2-phenylethyl)chromone derivatives from the seeds of *Cucumis melo* L var. *reticulatus*. *Natural Product Communications*, *5*(3), 403−406.

Ibrahim, S. R. M. (2014). New chromone and triglyceride from *Cucumis melo* seeds. *Natural Product Communications*, *9*(2), 205−208.

Ibrahim, S. R. M., & Mohamed, G. A. (2015). Cucumin S, a new phenylethyl chromone from *Cucumis melo* var. *reticulatus* seeds. *Revista Brasileira de Farmacognosia*, *25*(5), 462−464.

Ibrahim, S. R. M., Khedr, A. I. M., Mohamed, G. A., Zayed, M. F., El-Kholy, A. A. S., & Al Haidari, R. A. (2019). Cucumol B, a new triterpene benzoate from *Cucumis melo* seeds with cytotoxic effect toward ovarian and human breast adenocarcinoma. *Journal of Asian Natural Products Research*, *21*(11), 1112−1118.

Ibrahim, S., Al Haidari, R., Mohamed, G., Elkhayat, E., & Moustafa, M. (2016). Cucumol A: A cytotoxic triterpenoid from *Cucumis melo* seeds. *Revista Brasileira de Farmacognosia*, *26*(6), 701−704.

Ibrahim, T. A., El-Hefnawy, H. M., & El-Hela, A. A. (2010). Antioxidant potential and phenolic acid content of certain cucurbitaceous plants cultivated in Egypt. *Natural Product Research*, *24*(16), 1537−1545.

Idemudia, O. U., & Enogieru, A. B. (2024). Phytochemical and pharmacological activities of *Cucumis sativus*: An updated review. *Tropical Journal of Natural Product Research*, *8*(7), 7612−7623.

Itoh, T., Kikuchi, Y., Shimizu, N., Tamura, T., & Matsumoto, T. (1981). 24*β*-Ethyl-31-norlanosta-8,25(27)-dien-3*β*-ol and 24*β*-ethyl-25(27)-dehydrolophenol in seeds of three Cucurbitaceae species. *Phytochemistry*, *20*(8), 1929−1933.

Itoh, T., Shigemoto, T., Shimizu, N., Tamura, T., & Matsumoto, T. (1982). Triterpene alcohols in the seeds of two *Cucumis* species of Cucurbitaceae. *Phytochemistry*, *21*(9), 2414−2415.

Jevtić, B., Djedović, N., Stanisavljević, S., Gašić, U., Mišić, D., Despotović, J., et al. (2017). Anti-encephalitogenic effects of cucumber leaf extract. *Journal of Functional Foods*, *37*, 249−262.

Jianhua, Q., Kaiyue, S., Huan, C., Lan, X., & Lijuan, G. (2013). *Preparation and application of Pedicellus Melo. tetracyclic triterpenoid cucurbitacin type compound* (CN103360452A).

Kato-Noguchi, H., Le Thi, H., Sasaki, H., & Suenaga, K. (2012). A potent allelopathic substance in cucumber plants and allelopathy of cucumber. *Acta Physiologiae Plantarum*, *34*(5), 2045−2049.

Kavishankar, G. B., & Lakshmidevi, N. (2014). Anti-diabetic effect of a novel N-trisaccharide isolated from *Cucumis prophetarum* on streptozotocin−nicotinamide induced type 2 diabetic rats. *Phytomedicine*, *21*(5), 624−630.

Kemp, T. R. (1977). A C_15_ aldehyde from *Cucumis sativus*. *Phytochemistry*, *16*(11), 1831−1832.

Khetkam, P., Xie, X. N., Kisugi, T., Kim, H. I., Yoneyama, K., Uchida, K., et al. (2014). 7*α*- and 7*β*-Hydroxyorobanchyl acetate as germination stimulants for root parasitic weeds produced by cucumber. *Journal of Pesticide Science*, *39*(3), 121−126.

Kintia, P. K., & Wojciechowski, Z. A. (1975). Pentacyclic triterpenes and typical sterol precursors in *Cucumis sativus* seedlings. *Phytochemistry*, *14*(1), 296−297.

Kintya, P. K., Isaeva, N. E., Chirva, V. Y., & Lazur’evskii, G. V. (1972). Bitter substances of cucumbers. *Chemistry of Natural Compounds*, *8*(3), 303−305.

Knights, B. A., & Smith, A. R. (1977). Sterols of male and female flowers of *Cucumis sativus*. *Planta*, *134*(2), 115−117.

Krauze-Baranowska, M., & Cisowski, W. (2001). Flavonoids from some species of the genus *Cucumis*. *Biochemical Systematics and Ecology*, *29*(3), 321−324.

Lata, S., & Mittal, S. K. (2017a). Identification of isolated flavonoid glycoside from methanolic extract of *Cucumis dipsaceus* Ehrenb. (fruit). *International Journal of Pharmacognosy and Phytochemical Research*, *9*(7), 1051−1059.

Ma, Q. G., & Wei, R. R. (2021a). A new anthraquinone-aurone adduct with hepatoprotective activity from the fruits of *Cucumis bisexualis*. *Chemistry of Natural Compounds*, *57*(5), 828−831.

Ma, Q. G., & Wei, R. R. (2021b). Isolation and characterization of hepatoprotective anthraquinone derivatives from *Cucumis bisexualis*. *Chemistry of Natural Compounds*, *57*(4), 627−630.

Ma, Q. G., & Wei, R. R. (2023). Isolation and characterization of auronolignan derivatives with hepatoprotective activities from *Cucumis bisexualis*. *Chemistry of Natural Compounds*, *59*(2), 230−233.

Ma, Q. G., Liu, W. M., & Wei, R. R. (2024). Isolation and characterization of flavonolignan from *Cucumis bisexualis* and their hepatoprotective activities. *Chemistry of Natural Compounds*, *60*(6), 1016−1020.

Ma, Q. G., Liu, W. M., Sang, Z. P., & Wei, R. R. (2025). Hepatoprotective biphenyl derivatives from *Cucumis bisexualis*. *Chemistry of Natural Compounds*, *61*(1), 75–79.

Ma, Q. G., Wei, R. R., & Sang, Z. P. (2020a). Bioactivity-guided isolation of aurone derivatives with hepatoprotective activities from the fruits of *Cucumis bisexualis*. *Zeitschrift Fur Naturforschung - C Journal of Biosciences*, *75*(9−10), 327−332.

Ma, Q. G., Wei, R. R., & Sang, Z. P. (2020b). Hepatoprotective homoisoflavonoids from the fruits of *Cucumis bisexualis*. *Journal of Food Biochemistry*, *44*(7), e13264.

Ma, Q. G., Wei, R. R., & Sang, Z. P. (2020c). Structural characterization and hepatoprotective activity of naphthoquinone from *Cucumis bisexualis*. *Natural Product Communications*, *15*(1).

Ma, Q. G., Wei, R. R., Sang, Z. P., & Dong, J. H. (2021). Structurally diverse coumarin-homoisoflavonoid derivatives with hepatoprotective activities from the fruits of *Cucumis bisexualis*. *Fitoterapia*, *149*, 104812.

Ma, Q. G., Wei, R. R., Yang, M., Huang, X. Y., Wang, F., Sang, Z. P., et al. (2018). Molecular characterization and bioactivity of coumarin derivatives from the fruits of *Cucumis bisexualis*. *Journal of Agricultural and Food Chemistry*, *66*(22), 5540−5548.

Maja, D., Mavengahama, S., & Mashilo, J. (2022). Cucurbitacin biosynthesis in cucurbit crops, their pharmaceutical value and agricultural application for management of biotic and abiotic stress: A review. *South African Journal of Botany*, *145*, 3−12.

Mashchenko, N. E., Kintya, P. K., & Lazur’evskii, G. V. (1975). Sterols of various organs of *Cucumis sativus*. *Chemistry of Natural Compounds*, *11*(5), 697.

Mashchenko, N. E., Kintya, P. K., Dragalin, I. P., Lazur’evskii, G. V., Demakova, T. V., & Guseva, L. I. (1976). Methods of analyzing cucurbitacins in cucumbers. *Chemistry of Natural Compounds*, *12*(2), 239−240.

Matsumoto, T., Shigemoto, T., & Itoh, T. (1983a). (22*E*,24*S*)-5*α*-Ergosta-7,22-dien-3*β*-ol from the seeds of *Cucumis sativus*. *Phytochemistry*, *22*(5), 1300−1301.

Matsumoto, T., Shigemoto, T., & Itoh, T. (1983b). Occurrence of 24-ethyl-∆^5^- and 24-ethyl-∆^7^-sterols as C-24 epimeric mixtures in seeds of *Cucumis sativus*. *Phytochemistry*, *22*(11), 2622−2624.

McNally, D. J., Wurms, K. V., Labbé, C., & Bélanger, R. R. (2003). Synthesis of *C*-glycosyl flavonoid phytoalexins as a site-specific response to fungal penetration in cucumber. *Physiological and Molecular Plant Pathology*, *63*(6), 293−303.

Milder, I. E. J., Arts, I. C. W., van de Putte, B., Venema, D. P., & Hollman, P. C. H. (2005). Lignan contents of Dutch plant foods: A database including lariciresinol, pinoresinol, secoisolariciresinol and matairesinol. *British Journal of Nutrition*, *93*(3), 393−402.

Miró, M. (1995). Cucurbitacins and their pharmacological effects. *Phytotherapy Research*, *9*(3), 159−168.

Mukherjee, P. K., Nema, N. K., Maity, N., & Sarkar, B. K. (2013). Phytochemical and therapeutic potential of cucumber. *Fitoterapia*, *84*(1), 227−236.

Nash, R. J., Bartholomew, B., Penkova, Y. B., Rotondo, D., Yamasaka, F., Stafford, G. P., et al. (2020). Iminosugar idoBR1 isolated from cucumber *Cucumis sativus* reduces inflammatory activity. *ACS Omega*, *5*(26), 16263−16271.

Nigussie, G., & Ashenef, S. (2020). Isolation, characterization, structural elucidation and anti-bacterial activities of roots extracts of *Cucumis ficifolius*. *Research Square*, 1−29.

Olennikov, D. N. (2023). Separation, characterization and mammal pancreatic lipase inhibitory potential of cucumber flower flavonoids. *Separations*, *10*(4), 255.

Olennikov, D. N., & Kashchenko, N. I. (2023a). Acylated flavonoids from *Cucumis sativus* inhibit the activity of human pancreatic lipase. *Applied Biochemistry and Microbiology*, *59*(4), 530−538.

Olennikov, D. N., & Kashchenko, N. I. (2023b). Green waste from cucumber (*Cucumis sativus* L.) cultivation as a source of bioactive flavonoids with hypolipidemic potential. *Agronomy*, *13*(9), 2410.

Olennikov, D. N., & Kashchenko, N. I. (2023c). New flavonoids from *Cucumis sativus*. *Chemistry of Natural Compounds*, *59*(4), 651−654.

Olennikov, D. N., & Kashchenko, N. I. (2024a). New acylated *C*,*O*-glycosylflavones from *Cucumis sativus*. *Chemistry of Natural Compounds*, *60*(2), 235−240.

Olennikov, D. N., & Kashchenko, N. I. (2024b). Minor *C*,*O*-glycosylflavones from *Cucumis sativus*. *Chemistry of Natural Compounds*, *60*(5), 823−827.

Pharr, D. M., Hendrix, D. L., Robbins, N. S., Gross, K. C., & Sox, H. N. (1987). Isolation of galactinol from leaves of *Cucumis sativus*. *Plant Science*, *50*(1), 21−26.

Qing, Z. X., Shi, Y., Han, L. D., Li, P. K., Zha, Z. O., Liu, C., et al. (2022). Identification of seven undescribed cucurbitacins in *Cucumis sativus* (cucumber) and their cytotoxic activity. *Phytochemistry*, *197*, 113123.

Rao, M. G., & Row, L. R. (1968). Isolation of cucurbitacin C from *Cucumis prophetarum* Linn. *Current Science*, *37*(13), 361−363.

Rayle, D. L., & Purves, W. K. (1967). Isolation and identification of indole-3-ethanol (tryptophol) from cucumber seedlings. *Plant Physiology*, *42*(4), 520−524.

Rehm, S., Enslin, P. R., Meeuse, A. D. J., & Wessels, J. H. (1957). Bitter principles of the Cucurbitaceae. VII.—The distribution of bitter principles in this plant family. *Journal of the Science of Food and Agriculture*, *8*(12), 679−686.

Satoh, J., Koshino, H., Sekino, K., Ito, S., Katsuta, R., Takeda, K., et al. (2016). *Cucumis sativus* secretes 4'-ketoriboflavin under iron-deficient conditions. *Bioscience, Biotechnology, and Biochemistry*, *80*(2), 363−367.

Segarra, G., Jáuregui, O., Casanova, E., & Trillas, I. (2006). Simultaneous quantitative LC−ESI-MS/MS analyses of salicylic acid and jasmonic acid in crude extracts of *Cucumis sativus* under biotic stress. *Phytochemistry*, *67*(4), 395−401.

Shang, Y., Ma, Y. S., Zhou, Y., Zhang, H. M., Duan, L. X., Chen, H. M., et al. (2014). Biosynthesis, regulation, and domestication of bitterness in cucumber. *Science*, *346*(6213), 1084−1088.

Smith, V. A., Sponsel, V. M., Knatt, C., Gaskin, P., & MacMillan, J. (1991). Immunochromatographic purification of gibberellins from vegetative tissues of *Cucumis sativus* L: Separation and identification of 13-hydroxy and 13-deoxy gibberellins. *Planta*, *185*(4), 583−586.

Tamiru, E., Temesegen, A., & Demise, D. (2019). Phytochemical investigation on the root extraction of *Cucumis prophetarum* L. *Chemistry Africa*, *2*(3), 351−360.

Tang, J., Meng, X. J., Liu, H., Zhao, J. L., Zhou, L. G., Qiu, M. H., et al. (2010). Antimicrobial activity of sphingolipids isolated from the stems of cucumber (*Cucumis sativus* L.). *Molecules*, *15*(12), 9288−9297.

Tunmann, P., & Frank, W. (1972). Steringlucosidfettsäureester in der frucht von *Cucumis sativus* L. *Archiv Der Pharmazie*, *305*(6), 469−471.

Ul Haq, F., Ali, A., Khan, M. N., Shah, S. M. Z., Kandel, R. C., Aziz, N., et al. (2019). Metabolite profiling and quantitation of cucurbitacins in Cucurbitaceae plants by liquid chromatography coupled to tandem mass spectrometry. *Scientific Reports*, *9*(1), 15992.

Ulubelen, A., Baytop, T., & Çubukcu, B. (1976). Identification of steroidal and triterpenic compounds of *Cucumis trigonus*. *Planta Medica*, *30*(6), 144−145.

Yuan, R. Q., Qian, L., Yun, W. J., Cui, X. H., Lv, G. X., Tang, W. Q., et al. (2019). Cucurbitacins extracted from *Cucumis melo* L. (CuEC) exert a hypotensive effect via regulating vascular tone. *Hypertension Research*, *42*(8), 1152−1161.

Zhou, X. J., Li, X. S., Shen, Y., Pei, G., Wang, J. F., & Cheng, Y. X. (2012). Steroids and triterpenoids from *Cucumis sativus* roots. *Chemistry of Natural Compounds*, *48*(3), 419−422.

Zhu, M. Q., Huang, R. M., Wen, P., Song, Y., He, B. L., Tan, J. L., et al. (2021). Structural characterization and immunological activity of pectin polysaccharide from kiwano (*Cucumis metuliferus*) peels. *Carbohydrate Polymers*, *254*, 117371.
